# Human papillomavirus self-sampling versus provider-sampling in low- and middle-income countries: a scoping review of accuracy, acceptability, cost, uptake, and equity

**DOI:** 10.3389/fpubh.2024.1439164

**Published:** 2024-11-29

**Authors:** Jenifer Akoth Otieno, Lisa Were, Moriasi Nyanchoka, Easter Olwanda, Mercy Mulaku, Xiaohui Sem, Mikashmi Kohli, Jessica Markby, Angela Muriuki, Eleanor Ochodo

**Affiliations:** ^1^Center for Global Health Research, Kenya Medical Research Institute (KEMRI), Kisumu, Kenya; ^2^Health Economics Research Unit, KEMRI-Wellcome Trust Research Programme, Nairobi, Kenya; ^3^Department of Pharmacology, Clinical Pharmacy, and Pharmacy Practice, Faculty of Health Sciences, University of Nairobi, Nairobi, Kenya; ^4^FIND, Geneva, Switzerland; ^5^Center for Evidence-Based Health Care, Department of Epidemiology and Biostatistics, Faculty of Medicine and Health Sciences, Stellenbosch University, Stellenbosch, South Africa

**Keywords:** human papillomavirus (HPV), self-sampling, provider-sampling, cervical cancer, accuracy, acceptability, cost, uptake

## Abstract

**Introduction:**

HPV self-sampling is a relatively new, cost-effective and widely accepted method, however, uptake in LMICs remains limited. We aimed to map out the evidence and identify gaps in accuracy, acceptability, cost, equity and uptake of self-sampling vs. provider-sampling in LMICs.

**Methods:**

We searched: MEDLINE, EMBASE, CINAHL, SCOPUS, Web of Science, and Global Index Medicus, from 1946 to July 2023. Inclusion criteria entailed studies focusing on self-sampling alone or compared to provider-sampling for HPV testing and reporting on at least one outcome of interest (accuracy, acceptability, cost, equity, or uptake). Two authors independently screened titles, abstracts, and full texts, resolving disagreements through discussion. Data was extracted by one reviewer independently, with quality checks by senior authors, and results were synthesised narratively.

**Results:**

Our search yielded 3,739 records, with 124 studies conducted on 164,165 women aged 15–88 years between 2000 and 2023 included. Most studies were from the African region (*n* = 61, 49.2%). Designs included cross-sectional (*n* = 90, 81.1%), randomised (*n* = 5, 4.5%), modelling (*n* = 4, 3.6%), micro-costing (*n* = 2, 1.8%), and non-randomised crossover (*n* = 1, 0.9%) studies. Outcomes included; acceptability (*n* = 79, 63.7%), accuracy (*n* = 51, 41.1%), cost (*n* = 7, 5.6%), and uptake (*n* = 7, 5.6%). Most studies reported that participants preferred self-sampling, with only a few studies (*n* = 7, 8.9%) studies favouring provider-sampling. The sensitivity and specificity of self-sampling ranged from 37.5–96.8% and 41.6–100.0%, respectively. One study directly compared the sensitivity and specificity of dry self-collected vs. wet provider-collected sample transportation. Laboratory costs were similar, but overall costs were lower for self-sampling. Uptake was higher for self-sampling in five of the seven studies. Most studies (*n* = 106) mentioned equity factors like age (*n* = 69, 65.1%), education (*n* = 68, 64.2%) and place of residence (*n* = 59, 55.6%) but no analysis of their impact was provided.

**Conclusion:**

HPV self-sampling is acceptable and cost-effective but, evidence of its accuracy shows varying sensitivity and specificity. Evidence on the accuracy of dry self-collected vs. wet provider-collected sample transportation is limited. Research evaluating HPV self-sampling’s accuracy, including comparisons of transportation modes, uptake, the impact of equity factors in LMICs and comparisons with high-income countries is essential to inform cervical cancer screening uptake.

**Systematic review registration:**

https://doi.org/10.17605/OSF.IO/34TUY.

## 1 Introduction

Globally, cervical cancer is the fourth most prevalent cancer in women, with 604,127 incidences and 341,843 deaths recorded in 2020, and 84–90% of cases occurring in low- and middle-income countries (LMICs) (1–5). High-risk (hr) strains of human papillomavirus (HPV), particularly HPV-16 and HPV-18, are responsible for nearly 70% of cervical cancer cases, which can be detected through nucleic acid testing (6–12). Although prevention methods like vaccination and screening are available, the uptake of HPV screening in LMICs remains low (approximately 27%) due to various personal, social, and systemic challenges (13–16). This low rate of screening uptake is alarming, given that 84–90% of cervical cancer cases occur in these regions, underscoring a profound public health challenge (1–5). Despite the availability of preventive methods, several factors contribute to this gap in screening.

Personal, social, and systemic barriers significantly hinder HPV screening uptake in LMICs, where cervical cancer rates are disproportionately high (13–16). Many women in LMICs lack awareness of the link between HPV and cervical cancer, and fear, embarrassment, and stigma surrounding the screening process further reduce participation (15). Social and cultural factors, such as taboos around reproductive health and gender dynamics limiting women’s autonomy, also play a role (15). Additionally, programmatic challenges, including shortages of trained healthcare workers, limited access to facilities, and the high costs of provider-screening, exacerbate the problem (15). Considering these barriers, HPV self-sampling presents a promising alternative. This method offers a private, non-invasive, and cost-effective approach that reduces reliance on healthcare professionals and infrastructure, while empowering women to take control of the sample collection process (17). It thereby addresses personal, social, and systemic challenges to screening.

Traditional cytology testing has been the standard method for cervical cancer screening; however, it relies heavily on trained professionals for sample collection, which can pose challenges in resource-limited settings and reduce cancer screening uptake (18, 19). In contrast, HPV self-sampling has emerged as a promising alternative, offering women a convenient and private method for collecting samples (19, 20). Recent studies have indicated that self-sampled HPV tests demonstrate higher detection rates for cervical intraepithelial neoplasia grade 2 and above (CIN2+) compared to cytology (19). Notably, self-sampling has shown detection rates comparable to those of HPV and cytology co-testing, as evidenced by one non-randomised study and a randomised controlled trial (RCT) (19). Furthermore, findings from recent research revealed that repeated self-sampled HPV tests were associated with a two-fold increase in CIN2+ detection rates compared to cytology (19). HPV self-sampling provides conclusive results that enable the development of a streamlined protocol with well-defined management options (21).

HPV self-sampling not only demonstrates higher detection rates for CIN2+ compared to traditional cytology, but it also offers a more accessible and private alternative for women in various settings. This accessibility is largely due to the simplicity of the self-sampling process, which allows participants to collect samples with minimal clinical support. HPV self-sampling typically involves participants receiving a kit, collecting the sample, and sending it to a laboratory for testing, whether the sampling occurs at home, in a clinic, or another healthcare setting (17). Participants are provided with instructions for use, which may be verbal or written (17). The self-sampling process usually involves the participant collecting a vaginal swab, either with or without supervision (17). The swab is inserted into the vagina, and the participant collects the sample by moving the swab in a circular motion (17). After collection, the swab is placed into a sample collection tube, which may or may not contain a transportation liquid (17). Variations exist regarding sample transportation across different contexts including use of postal services, delivery companies, or local health services (16, 17).

Provider-sampling has been the conventional strategy for HPV testing and considered more accurate as it is collected by trained medical professionals (22, 23). However, concerns have been reported because of the sample collection’s invasive nature, cost and access limitations (24, 25). Self-sampling, a relatively newer approach, has been reported to be widely accepted compared to provider-sampling, particularly in LMICs (17). This method addresses many acceptability concerns associated with provider-sampling, including invasiveness, discomfort, cultural sensitivities and embarrassment (26–30). It may also be preferable in resource-limited regions because of its cost-effectiveness, relative feasibility and sustainability (31). It provides an opportunity for increased screening coverage and early detection of HPV, especially in LMIC settings where access to healthcare facilities and trained health workers is limited (16).

Globally, current research examining the accuracy of HPV self-sampling vs. provider-sampling presents conflicting findings (4, 16, 22, 32, 33). A primary study by Feng et al., 2022 demonstrated benefits in the accuracy of HPV self-sampling; however, another meta-analysis reported that provider-sampling was superior in accuracy (4, 32). Some studies state that self and provider-sampling have similar accuracy effects (16, 22, 33). Most of these studies were carried out in high-income countries, covering approximately 63%, which results in knowledge gaps in LMICs (15). Staley et al. (34), conducted a Cochrane review and meta-analysis in 2021 on interventions targeting women to encourage the uptake of cervical cancer screening. They included 69 trials in the analysis and found that most evidence (97%) was reported from high-income countries (34). Therefore, evidence on the uptake of cervical cancer screening through self-sampling in LMIC remains unclear.

Mekuria et al. (30) is a systematic review and meta-analysis that included six studies from Uganda, Nigeria, Ethiopia, Mexico, Brazil and Argentina (30). It reported that HPV self-sampling increased uptake of cervical cancer screening, particularly in low-income countries (30). While Mekuria et al. (30) focused on examining the effect of HPV self-sampling on screening uptake and estimating associated costs in LMICs, their review did not address other critical measures such as accuracy, acceptability, and equity. Additionally, their analysis included fewer studies—six, leaving a gap in the comprehensive evaluation of HPV self-sampling compared to provider-sampling (30). While HPV self-sampling is a promising solution, there is still a lack of comprehensive data on its accuracy, acceptability, cost, and the impact of equity factors such as socioeconomic factors on cervical cancer screening uptake in LMICs. This scoping review aimed to address these gaps by providing a comprehensive evaluation of HPV self-sampling compared to provider-sampling in LMICs. It focused on critical dimensions such as test acceptability, accuracy, cost, and the impact of health equity factors on the test’s uptake. By doing so, this study sought to inform public health strategies to improve cervical cancer screening in resource-limited settings.

## 2 Methods

This scoping review was conducted according to the framework by Arksey and O’Malley (35). Our review followed the guidance by the Preferred Reporting Items for Systematic Reviews and Meta-Analyses for Scoping Reviews (PRISMA-Scry) (36). See Supplementary Annex 1 for the PRISMA-ScR checklist (36). Our protocol was submitted to the OSF registries.^1^

### 2.1 Eligibility criteria

Eligibility was defined by the population, concept, context (PCC) framework as follows:

*Population and setting:* Studies conducted on female participants at risk of HPV, regardless of age, gender, HIV status or cervical cancer screening status within LMICs were included. Studies involving populations confirmed to have invasive cancer were excluded from our review because HPV self-sampling is primarily designed for early detection and screening of HPV infection, rather than for individuals already diagnosed with invasive cancer.

*Concept:* Studies reporting on the outcomes of cost, acceptability, health equity, and uptake of HPV self-sampling were included regardless of whether they compared self-sampling to provider sampling. However, studies focusing on accuracy were restricted to those that specifically compared HPV self-sampling to provider sampling. Accuracy was defined by both; test sensitivity—the ability of the self-sampling method to correctly identify individuals who have an HPV infection and test specificity— ability of the self-sampling method to correctly identify individuals who do not have an HPV infection (37). Cost was defined as the estimated expense (in US dollars for HPV testing using self-sampling or provider-sampling (20). Acceptability was defined by how well HPV self-sampling was received by the target population, including their willingness, comfort and overall satisfaction using the test (38). Uptake was defined as how many eligible individuals chose to participate in the self-sampling process, as a percentage of those that were eligible to participate (39). Health equity was defined as fair access to HPV self-sampling regardless of socioeconomic or demographic factors to reduce disparities in access to testing (40). Studies reporting alternative cervical cancer screening or testing strategies to self-sampling were excluded.

*Context:* Studies conducted in at least one of the LMICs were included based on the World Bank classification (41). Those conducted in high-income countries were excluded for falling outside the scope of this review (Supplementary Annex 2).

*Types of studies:* Published studies written in English or those with accessible English translations, regardless of their randomisation unit, the presence of a control or multiple comparators were identified. Quantitative (experimental and observational), qualitative and mixed-methods studies were considered. Studies reporting on accuracy data or two-by-two tables were limited to those evaluating self-sampling compared to provider-sampling, regardless of the reference test option. Modelling studies reporting on cost and conference abstracts published in conference proceedings with their corresponding full-text papers were also considered. Conference abstracts only, traditional literature reviews, editorials with insufficient information, case reports and opinion papers were excluded because of the increased risk of bias within these article types.

### 2.2 Study identification

Cochrane’s information specialist (VL) performed the electronic searches. The following databases were searched from 1946 to 10 July 2023: MEDLINE (via OVID), EMBASE (via OVID), CINAHL (via EBSCOhost), SCOPUS, Web of Science and Global Index Medicus. The following clinical trial registries were also searched: CENTRAL (Cochrane Central Register of Controlled Trials), ClinicalTrials.gov, WHO Trials Register—International Clinical Trials Registry Platform (ICTRP) and the pre-print server for health sciences-MedRxiv.

Other searches were conducted through the reference lists of the relevant secondary studies and grey literature from the following websites: WHO,^2^ International AIDS Society (IAS)^3^ and the International Agency for Research on Cancer (IARC).^4^ The following search terms alongside their synonyms were applied during the search: “human papillomavirus (HPV),” “self-collected sample,” “provider-collected sample” “cost benefit analysis,” “healthcare access,” “health equity,” “socioeconomic parameters,” “patient acceptance of health care,” and “preferences.” See Supplementary Annex 3 for the full search strategy.

### 2.3 Study selection

Two independent reviewers (any pair from JO, LW, EEO, and MN) screened the titles and abstracts of all eligible studies using Covidence—a systematic review management software to identify relevant studies (42). After that, two independent reviewers (any pair from JO, LW, EEO, and MN) retrieved and screened full texts of the remaining studies against our eligibility criteria. All disagreements were resolved by discussion between the reviewers (JO, LW, EEO, and MN) or by consulting senior reviewers (MM and EO) for consensus. To minimise bias, senior reviewers (MM and EO) checked a random sample (20%) of the included studies.

### 2.4 Data charting

Two independent reviewers (any pair from JO, LW, EEO, and MN) piloted the data extraction form of five eligible studies. However, data extraction was done by at most one reviewer (either JO, LW, EEO, or MN) using a pre-designed data extraction form in Covidence (42). A senior reviewer (MM) conducted quality checks on a random sample (10%) of the extracted data. Discrepancies were resolved by discussion between the reviewers (JO, LW, EEO, and MN) or by consulting a senior reviewer (MM and EO) for a consensus.

Data was charted on the following aspects:

General study details: title, objectives, lead author’s surname, country, WHO region (Supplementary Annex 2)—and year of publication.

General study characteristics: study type, design, participant description (number and age), target disease, index test (HPV self-sampling) and comparator/reference test (HPV provider-sampling) particulars (assay, sample type, diagnostic test, collection device, sample transportation mode and manufacturer), outcomes (accuracy—sensitivity and specificity, acceptability, cost-effectiveness, financial costs and uptake), health facility setting and facility ownership.

Relevant PROGRESS-Plus factors across all studies, as guided by the PROGRESS-Plus framework for Health Equity, that is, Place of residence, Race/ethnicity/culture/language, Occupation, Gender identity, Religion, Education, Socioeconomic status and Social capital (40). The Plus factors included age, disability and comorbidity. See Supplementary Annex 4 for a detailed definition of the PROGRESS-Plus equity factors.

### 2.5 Quality appraisal

The methodological quality or risk of bias in all included studies was not assessed, as the scoping review guidance does not recommend it and our review solely aimed to map the existing literature (35). Nevertheless, two independent reviewers (any pair from JO, LW, EEO, and MN) appraised the quality of included diagnostic accuracy studies to guide the interpretation of findings during narrative synthesis. The appraisal was based on the reporting of the 10 items across the four domains of the QUADAS-2 tool (43).

### 2.6 Data synthesis

STATA version 17 (Stata Corp LLC, College Station, TX) was utilised to descriptively summarise quantitative data on diagnostic accuracy, acceptability, cost, uptake, and health equity factors. Qualitative data was synthesised using thematic analysis and reported in narrative form. Findings were presented using tables and graphs. Recommendations for future research and implications for policy and practice were provided based on the identified evidence gaps.

## 3 Results

### 3.1 Study selection

**Figure 1:**
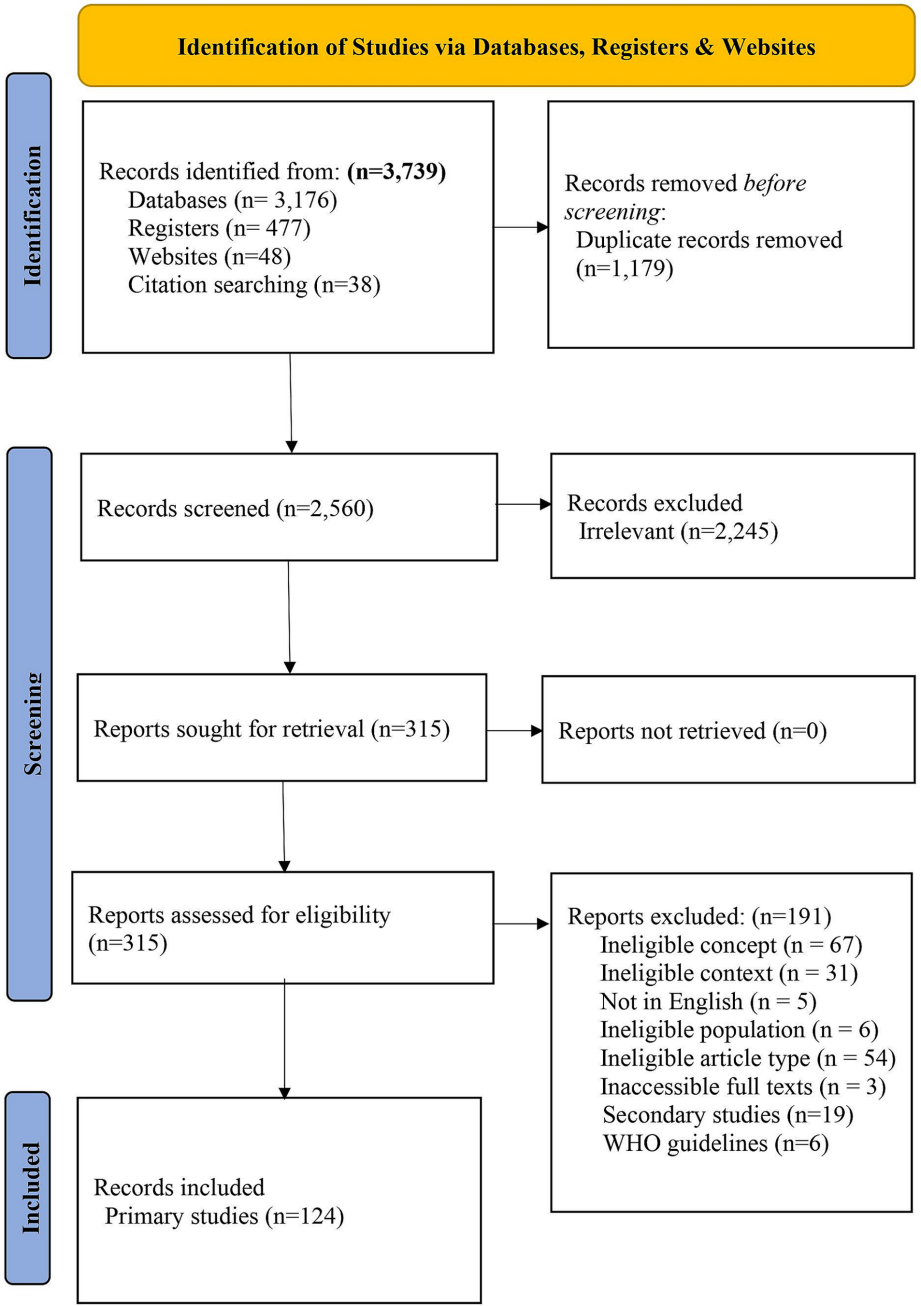
The PRISMA 2020 flow chart showing study selection process.

Our search yielded 3,739 articles, of which 1,179 duplicates were excluded. Screening involved 2,560 titles and abstracts, resulting in the exclusion of 2,245 studies. We screened 315 full-text articles from which we excluded 191 (Figure 1 and Supplementary Annex 5) and included 124 primary studies.

### 3.2 General characteristics of included studies and guidelines

The included studies (*n* = 124, 100%) were published between 2000 and 2023 (Supplementary Annex 6). Most studies (*n* = 123, 99.2%) were done on women aged between 15 and 88 years, except for one on transgender men—individuals identifying as male who were assigned female sex at birth (44). Many studies were conducted within the African region (*n* = 61, 49.2%). No studies were identified from the European or Eastern Mediterranean regions. By country, the highest number of studies were conducted in China (*n* = 13, 10.5%) and South Africa (*n* = 12, 9.7%), both of which are considered upper-middle-income countries.

A large proportion of studies were conducted in healthcare facilities (*n* = 97, 78.2%), with some taking place in community health posts (*n* = 31, 25.0%) and fewer in-home settings (*n* = 8, 6.5%). The majority were quantitative studies (*n* = 111, 89.9%), followed by qualitative (*n* = 7, 5.6%) and mixed methods (*n* = 6, 4.8%). Among the quantitative studies, cross-sectional designs were the most common (*n* = 90, 81.1%), followed by cohort studies (*n* = 9, 8.1%), randomised trials (*n* = 5, 4.5%), modelling (*n* = 4, 3.6%), micro-costing (*n* = 2, 1.8%), and non-randomised crossover trials (*n* = 1, 0.9%).

Studies primarily reported on acceptability (*n* = 79, 63.7%, 71,418 participants), followed by accuracy (*n* = 51, 41.1%, 73,618 participants), cost (*n* = 7, 5.6%, 11,593 participants), and uptake (*n* = 7, 5.6%, 17,784 participants). Although no studies assessed equity as an outcome, the majority (*n* = 106, 85.5%) mentioned at least one equity factor. Nearly all studies (*n* = 118, 95.2%) were conducted in single-country settings, with a few (*n* = 6, 4.8%) involving multiple countries (Mexico, Peru, Malawi, South Africa, Argentina, and Brazil).

All studies (*n* = 124, 100.0%) conducted HPV DNA for the self-collected samples. For HPV provider-sampling, most studies (*n* = 58, 46.8%) assessed HPV DNA testing, with few studies (*n* = 6, 4.8%) reported on HPV messenger ribonucleic acid (mRNA) testing. The remaining 60 studies did not report on diagnostic tests for provider-sampling. The Digene HC2 (*n* = 23, 33.3%), careHPV and Xpert (each *n* = 11, 15.9%) and Aptima (*n* = 5, 7.2%) assays were the most frequently reported tests used for both self-sampling and provider-sampling. Most studies reported using brushes for specimen collection (*n* = 71, 57.3%), followed by swabs (*n* = 44, 35.5%) and nine did not report on the collection device. Of all studies included (*n* = 62, 50.0%) used wet and (*n* = 17, 13.7%) used dry self-collected sample transportation. Forty-five studies did not report on the mode of sample transportation. Only one study directly compared the sensitivity and specificity of dry vs. wet self- and provider-collected sample transportation modes, respectively.

### 3.3 Summary of findings

#### 3.3.1 Acceptability

Most studies (*n* = 66, 84.6%) reported quantitative outcomes on HPV self-sampling (Table 1) of which (*n* = 53, 80.3%) reported that more than half of the participants were willing to obtain the samples themselves (57.7–100.0%). A few quantitative studies (*n* = 12, 18.2%) reported that participants preferred provider-sampling (54.8–83.8%). One study reported that the acceptability of self-sampling was 95.6% in rural areas and 79% in urban areas (45). Studies reporting qualitatively on HPV self-sampling acceptability were (*n* = 11, 13.9%). The central theme of these qualitative studies revolved around the acceptability of HPV self-sampling, giving rise to eight sub-themes as follows: (Supplementary Annex 7).

**Table 1:** Acceptability of HPV self-sampling in LMICs.

Author, year (Sample size, *N*)	Country	Population	Self-sampling acceptability; %, *p*-value	Provider-sampling acceptability; %, *p*-value
Abdullah 2018 (62) (*N* = 164)	Malaysia	Eligible women consenting to self-collection during the initial visit to the health facility	93.2%, *p*-value not reported	Not reported
Adamson 2015 (98) (*N* = 325)	South Africa	WLHIV aged 25 years or older without a cervical cytology test in the last three years in a healthcare facility	10.1%, *p*-value not reported	54.8%, *p* < 0.001
Afzal 2020 (99) (*N* = 120)	Liberia	Women presenting for cervical cancer screening in health care facility	66.7%, *p*-value not reported	31.1%, *P*-value not reported
Ahmad 2021 (100) (*N* = 220)	Malaysia	Women in reproductive age group in health care facility	84.0%, *P*-value not reported	Not reported
Allende 2019 (101) (*N* = 222)	Bolivia	Women from urban, peri-urban and rural areas with an age range between 25 and 64 years	71.6%, p > 0.05	40.5%, *p* > 0.05
Anand 2022 (102) (*N* = 485)	India	Sexually active non-pregnant women aged 30–55 years with no history of cervical cancer undergoing home-based testing	97.0%, *P*-value not reported	Not reported
Arrossi 2016 (51) (*N* = 3,049)	Argentina	Women present at home during cervical cancer and HPV screening visit	86.0%, *P*-value not reported	Not reported
Awua 2017 (71) (*N* = 228)	Ghana	Healthy women (self-report) between ages 15 to 65 years in the health facility	22.6%, *P*-value not reported	56.2%, *P*-value not reported
Bansil 2014 (52) (*N* = 3,863)	India; Nicaragua; Uganda	Female staff members in health facilities also for home-based testing	77.5%, *P*-value not reported	22.5%, *P*-value not reported
Behnke 2020 (54) (*N* = 52)	Ghana	Women aged 30 to 65 years in health facility	98.1%, *P*-value not reported	Not reported
Berner 2013 (103) (*N* = 217)	Cameroon	Non-pregnant women with no history of hysterectomy in a health facility	29.0%, *P* < 0.001	62%, *p* < 0.001
Broquet 2015 (104) (*N* = 300)	Madagascar	Women aged 30 to 65 years in health care facility	100.0%, *P*-value not reported	Not reported
Castle 2019 (105) (*N* = 483)	Brazil	Women aged between 25 and 64 years in health care facility	99.4%, *P*-value not reported	Not reported
Dareng 2015 (106) (*N* = 581)	Nigeria	Women over 18 years with no obvious physical ailments in community-based screening	19.0%, *P*-value not reported	81.0%, *P*-value not reported
De Melo Kuil 2017 (107) (*N* = 171)	Brazil	Women aged 18 to 64 years referred for colposcopy in a health facility	58.8%, *P*-value not reported	28.8%, *P*-value not reported
Dzuba 2002 (108) (*N* = 1,069)	Mexico	Women aged 20 years and above in health facility	65.6%, *P*-value not reported	Not reported
Eamratsameekool 2023 (109) (*N* = 535)	Thailand	Women aged 30 to 59 years in health care facility	95.0%, *P*-value not reported	Not reported
Eche 2022 (110) (*N* = 386)	South Africa	Female students aged 18 to 65 years and in the College of Humanities screening at the university	57.70%, *P*-value not reported	Not reported
Esber 2017 (111) (*N* = 824)	Malawi	Women aged 19–39 years in community-based screening	67%, *P*-value not reported	Not reported
Esber 2018 (112) (*N* = 199)	Malawi	Women aged 18–49 years in health care facilities who had at least one genitourinary symptom	61.0%, *P*-value not reported	39.0%, *P*-value not reported
Flores 2003 (113) (*N* = 7,732)	Mexico	Women aged 20–80 years in health facilities with no prior diagnosis of CIN2/3, hysterectomy or treatment.	68%, *P*-value not reported	32%, *P*-value not reported
Flores 2021 (22) (*N* = 505)	Mexico; Peru	Sexually active women aged 30–65 years in a health facility with no history of medical or surgical treatment for cervical cancer	96.6%, *P*-value not reported	Not reported
Goldstein 2020 (114) (*N* = 600)	China	Women aged 35–65 years in health care facility	65.0%, *p* < 0.05	35.0%, *p* < 0.05
Gottschlich 2017 (115) (*N* = 202)	Guatemala	Women aged 25–54 years for community-based screening	80.0%, *p*-value not reported	Not reported
Gottschlich 2019 (116) (*N* = 267)	Thailand	Women attending a clinic aged 25–60 years	100.0%, *p*-value not reported	Not reported
Guan 2012 (117) (*N* = 174)	China	Non-pregnant women without a hysterectomy in the health facility	26.0%, *P*-value not reported	74.0%, *P*-value not reported
Haile 2019 (118) (*N* = 83)	Ethiopia	Women aged 20 years and older in a health facility; mentally competent with no history of cervical cancer	85.0%, *P*-value not reported	4.9%, *P*-value not reported
He 2020 (119) (*N* = 810)	China	Literate women in the health facility	42.76%, *P*-value not reported	Not reported
Hood 2020 (120) (*N* = 1,034)	Malawi	Community screening for women in the catchment area of a community hospital	66.0%, *P*-value not reported	Not reported
Huchko 2018 (121) (*N* = 2,898)	Kenya	Women aged 25–65 years for community screening	99.1%, *P*-value not reported	Not reported
Islam 2020 (58) (*N* = 400)	Kenya	Women aged 18 years and above in a health facility self-identifying as exchanging sex for payment	36.1%, *P*-value not reported	63.9%, *P*-value not reported
Katanga 2021 (122) (*N* = 464)	Tanzania	Women aged 25–60 years attending routine cervical cancer screening services in health facility	79.8%, *P*-value not reported	16.5%, *P*-value not reported
Khoo 2021 (123) (*N* = 725)	Malaysia	Women aged 35–45 years for community-based screening	83.0%, *P*-value not reported	3.3%, *P*-value not reported
Kohler 2019 (28) (*N* = 104)	Botswana	WLHIV aged 25 years or older attending routine healthcare appointments	95.2%, *P*-value not reported	Not reported
Laskow 2017 (124) (*N* = 60)	El Salvador	Women aged 30–59 years for community-based screening	68.0%, *P*-value not reported	Not reported
Li 2022 (125) (*N* = 20,103)	China	Women aged 30–59 years sexually exposed and non-pregnant in the health facility	62.4%, *P*-value not reported	Not reported
Madhivanan 2021 (126) (*N* = 120)	India	Women 30 years or older in a health facility with no history of cervical cancer screening in the last 3 years	59.3%, *P*-value not reported	28.0%, *P*-value not reported
Mahande 2021 (127) (*N* = 350)	Tanzania	Women aged 25–55 years in a health care facility and from community-based screening	99.4%, *P*-value not reported	Not reported
Mahomed 2014 (128) (*N* = 106)	South Africa	WLHIV aged 20–65 years in a healthcare facility	94.0%, *p* = 0.94	Not reported
Mandigo 2015 (129) (*N* = 493)	Haiti	Pre-menopausal women aged 30 to 50 years in community-based screening	96.5%, *P*-value not reported	3.50%, *P*-value not reported
Manguro 2018 (130) (*N* = 199)	Kenya	Women aged 18–45 years in a health care facility	32.0%, *P* < 0.001	68.0%, *P* < 0.001
Maza 2018 (131) (*N* = 1,989)	El Salvador	Women aged 30–59 years in community-based screening	98.6%, *P*-value not reported	Not reported
Maza 2020 (72) (*N* = 24)	El Salvador	Transgender men aged 18 years or older in a healthcare facility	95.8%, *P*-value not reported	Not reported
Mitchell 2011 (132) (*N* = 300)	Uganda	Women aged 30–65 years in community-based screening	80.6%, *P*-value not reported	Not reported
Mitchell 2017 (133) (*N* = 84)	Uganda	WLHIV aged 30 to 69 years attending routine HIV clinic	98.8%, *P*-value not reported	Not reported
Modibbo 2017 (67) (*N* = 400)	Nigeria	Married women aged 28–60 years in health facilities or for community screening	83.2%, *P*-value not reported	9.2%, *P*-value not reported
Mremi 2021 (134) (*N* = 1,108)	Tanzania	Women aged 25–60 years in a health care facility	94.0%, *p* = 0.063	Not reported
Murchland 2019 (135) (*N* = 956)	Guatemala	Women aged 18–60 years in community-based screening	Santiago: 91.1%, Livingston: 41.6%, *p* < 0.0001	Not reported
Oberlin 2018 (136) (*N* = 298)	South Africa	Women aged 18 years or older in a healthcare facility	Not reported	83.8%, *P* < 0.001
Obiri-Yeboah 2017 (137) (*N* = 194)	Ghana	Every 5th woman aged 18 years or older was systematically selected from a list of daily attendants in the clinic	57.70%, *P*-value not reported	Not reported
Oneko 2022 (138) (*N* = 706)	Tanzania	Women aged 18–55 years in outpatient clinics	69.7%, *P*-value not reported	Not reported
Oranratanaphan 2014 (139) (*N* = 100)	Thailand	Women aged 30–65 years in a healthcare facility	90.0%, *P*-value not reported	Not reported
Peedicayil 2014 (140) (*N* = 809)	India	Women aged 30–50 years in a health care facility and community-based screening	71.0%, *P*-value not reported	Not reported
Phoolcharoen 2018 (141) (*N* = 248)	Thailand	Women aged 30–70 years in a health care facility	80.8%, *P*-value not reported	Not reported
Possati-Resende 2020 (142) (*N* = 386)	Brazil	Rural dwellers women screened at home	95.6%, *P*-value not reported	Not reported
Qu 2023 (143) (*N* = 862)	China	Literate women over 25 years in a healthcare facility	33.0%, *P*-value not reported	27.0%, *P*-value not reported
Quincy 2012 (144) (*N* = 245)	Nicaragua	Non-pregnant women aged 25 to 60 years in a healthcare facility	81.1%, *P*-value not reported	Not reported
Rawat 2021 (57) (*N* = 64)	Uganda	Women 18 years and older in a health facility	86.0%, *P*-value not reported	Not reported
Rodrigues 2018 (145) (*N* = 153)	Brazil	Non –indigenous HIV- infected and un-infected women in a healthcare facility	87.0%, *P*-value not reported	Not reported
Rosenbaum 2014 (146) (*N* = 518)	El Salvador	Non-pregnant women aged 30–49 years in a healthcare facility and in community-based screening	38.8%, *P*-value not reported	31.9%, *P*-value not reported
Rositch 2012 (147) (*N* = 409)	Kenya	HIV-discordant couples reporting sex acts in the previous 3 months in a healthcare facility	84.0%	Not reported
Saidu 2019 (50) (*N* = 863)	Malawi, South Africa	Women from the general population seeking primary screening and a referral population for colposcopy because of abnormal screening results	33.8%, *P*-value not reported	45.1%, *P*-value not reported
Sormani 2022 (148) (*N* = 2,201)	Cameroon	Women aged 30–49 years in a health care facility	76.9%, *P*-value not reported	23.1%, *P*-value not reported
Taku 2020 (149) (*N* = 737)	South Africa	Women aged 30 years or older attending community health clinics for cervical cancer screening	Community-based clinic: 77.2%, Referral clinic: 83.0%, *P*-value not reported	Not reported
Tiiti 2021 (150) (*N* = 527)	South Africa	Women aged 18 years or older in a health facility	87.1%, *P*-value not reported	76.4%, *P*-value not reported
Vallely 2022 (151) (*N* = 4,285)	Papua New Guinea	Women aged 30–59 years attending cervical cancer screening services in health facility	99·9%, *P*-value not reported	Not reported
Van De Wijgert 2006 (53) (*N* = 450)	South Africa	Sexually active women aged 18 years or older in health facility	63.3%, *P*-value not reported	Not reported
Varun 2023 (45) (*N* = 374)	Rwanda	Women 18 years or older in a healthcare facility	Urban: 79.9%, Rural: 95.6%, *P* < 0.001	Not reported
Vega Crespo 2022 (152) (*N* = 120)	Ecuador	Women aged 18–70 years in a health facility	84.2%, *P*-value not reported	51.7%, *P*-value not reported
Wong 2018 (153) (*N* = 68)	China	Non-pregnant women aged 18 years or older in a health facility with no abnormal pap smear results or symptoms	65.6%, *P*-value not reported	34.4%, *P*-value not reported
Yoshida 2013 (154) (*N* = 290)	Lao PDR	Women working in provincial hospitals and district health offices screened at health facility	62.0%, *P*-value not reported	36.0%, *P*-value not reported

*Preference:* Eight studies presented this sub-theme, of which seven studies showed evidence supporting participants’ preferences of self-sampling to provider-sampling (46–52). Many women expressed a strong preference for self-sampling over provider-sampling due to the increased privacy and the ability to avoid the discomfort of being examined by a healthcare worker (46–52). The convenience and autonomy that self-sampling provided at home were key motivators for women, allowing them to perform the procedure on their terms, in their own time, and without needing to visit a healthcare facility (46–52). In another study, women preferred provider- to self-sampling because healthcare workers could examine and see any other abnormalities that would instead go unnoticed (50).

*Provider’s role in self-sampling:* Five studies reported under this sub-theme (46, 48, 52–54). Two of them demonstrated a lack of trust among the participants in their capacities to perform self-sampling correctly (46, 53). Additionally, the role of the providers in offering guidance and assurances was highlighted by the participants in two other studies (52, 54). In one study, women expressed a preference for having a healthcare provider present while they were performing self-sampling for medical tests (48).

*Privacy:* Five studies reported on this sub-theme (46, 48, 50, 53, 55). They noted that the participants felt self-sampling was private and less embarrassing than provider-sampling.

*Barriers to self-sampling acceptance:* Four studies reported barriers to the acceptability of self-sampling, amongst which one demonstrated participants’ fear of hurting themselves while obtaining the self-samples (52). In another study, self-sampling conducted at clinics was found to be more convenient than at-home settings for the participants (50). In the other two studies, participants reported the lack of need to screen for HPV due to a lack of symptoms and lack of immediate treatment initiation, assuming the sample was taken at home (29, 56).

*Social influence:* Four studies were on social impact, two of which showcased the challenges related to social stigmatisation that individuals face when attending health screenings (48, 56). Those attending the screenings feared that the community members perceived them as being ill (48, 56). Additionally, these studies report that the participants often require approval from their partners before taking self-samples (57). Issues regarding lack of confidentiality among the local healthcare providers who would openly discuss the test results were also reported (51).

*Perception:* Two studies demonstrated that participants had limited understanding and poor perception regarding self-sampling which contributed to low prioritization of cervical (55, 56). Cultural obligations regarding modesty impacted some women’s perceptions of self-sampling (56).

*Motivation for provider-sampling:* A study reported that participants were motivated to choose provider-sampling because they valued the expertise of healthcare workers, believing that these professionals were more capable of identifying complications and knowing where to refer more complex cases (55). The study highlighted that provider’s engagement in the sampling process, offered better guidance and reassurance, ultimately fostering greater acceptance and understanding among patients (55).

*Religious influence:* In one study, women, particularly wives to religious leaders, argued that self-sampling interfered with their religious beliefs and practices (56).

#### 3.3.2 Accuracy

Studies reported a wide range of sensitivity (37.5 to 96.8%) and specificity (41.6 to 100.0%) for HPV self-sampling compared to provider sampling (Table 2). Only one study directly reported the sensitivity and specificity of dry self-sampling^5^ vs. wet provider-sampling^6^ (58). In wet transportation, self-sampling had 85.0% sensitivity (95% CI: 66.0–96.0%) and 66.0% specificity (95% CI: 61–71%), while in dry transportation, sensitivity was 78.0% (95% CI: 58.0–91.0%) and specificity was 71.0% (95% CI: 66–76%) (58).

**Table 2:** Accuracy of HPV self-sampling in LMICs across included quantitative studies stratified by sample transportation mode comparisons between the index and the comparator test.

Author, year (design)	Setting	Population	Sample size (*N*)	Self-sampling Test assay (manufacturer) Sample type Collection device	Provider-sampling Test assay (manufacturer) Sample type Collection device	Reference test assay	Sensitivity (95% CI) of the self-sampling	Specificity (95% CI) of the self-sampling	Target condition
Islam 2020 (cross-sectional) (58)	Kenya (public hospital)	High-risk WLHIV aged 18 years and above; residing in Mombasa; self-identifying as exchanging sex for payment in cash or in-kind at the time of enrolment and able to provide informed consent	400	Aptima HPV mRNA assay (Hologic) Vaginal sample Evalyn and viba cytobrush (dry and wet^*^ collection tube, respectively)	Aptima HPV mRNA assay (Hologic) Cervical sample Cytobrush (wet collection tube)	Not reported	Wet: 85.0% (66.0–96.0%) Dry: 78.0% (58.0–91.0%)	Wet: 66% (61–71%) Dry: 71% (66–76%)	High-grade cervical lesion
Qin 2016 (cross-sectional) (155)	China (public hospital)	Women from the general population who had provided self-collected swabs during the follow-up visits were identified and targeted for this sub-study	606 of 2,500	Real-time PCR hrHPV DNA assay (Abbott Molecular) Cervicovaginal sample Brush (dry collection tube)	Real-time PCR hrHPV DNA assay (Abbott Molecular) Cervicovaginal sample Brush (wet collection tube)	Biopsy	87.10%	88.60%	hrHPV infection
Viviano 2018 (Cross-sectional) (156)	Cameroon (hospital; ownership not reported)	Women from the general population aged 30 to 49 years were recruited in a CC screening campaign	188	Xpert HPV DNA assay (Cepheid) Vaginal sample Swab (dry collection tube)	Xpert HPV DNA assay (Cepheid) Cervical sample Cervex-brush (wet collection tube)	Xpert HPV DNA assay	84.6% (49.0–96.9%)	95% (59.9–75.3%)	HSIL and LSIL
Chen 2016 (cross-sectional) (157)	China (public hospital)	High-risk WLHIV aged 18 years and older were recruited from an HIV/AIDS treatment clinic in Yunnan, China, from 2019 to 2020.	409	Real-time PCR hrHPV DNA assay (Abbott Molecular) Cervico-vaginal sample Evalyn brush (dry collection tube)	Real-time PCR hrHPV DNA assay (Abbott Molecular) Cervical sample Swab (wet collection tube)	Real-time PCR hrHPV DNA assay	96.80%	98.10%	hrHPV, CIN1, CIN2+, CIN3
Nilyanimit 2014 (cross-sectional) (158)	Thailand (public hospital)	Women from the general population were solicited during colposcopy clinic and routine clinic	101	LAMP HPV DNA assay (Toshiba) Vaginal sample Flocked swab (wet collection tube)	LAMP HPV DNA assay (Toshiba) Cervical sample Flocked swab (wet collection tube)	LAMP HPV DNA assay	90.20%	93.30%	hrHPV infection
Salmeron 2003 (cross-sectional) (159)	Mexico (public health centre)	Women from the general population attending CC screening services at any one of the 23 health units that make up the Morelos Cervical Cancer Screening Program.	7,868	Digene HC2 HPV DNA assay (Qiagen) Vaginal sample Dacron swab (wet collection tube)	Digene HC2 HPV DNA assay (Qiagen) Cervical sample Cytobrush (wet collection tube)	Colposcopy; Cytology; Digene HC2 HPV DNA assay	71.3% (61.3–79.6%)	89.2% (88.5–89.9%)	CIN 2 and CIN 3
Adamson 2015 (cross-sectional) (98)	South Africa (public health centre)	High-risk WLHIV, 25 years or older, who did not have a cervical cytology test result documented in their chart within the past three years	325	Aptima HPV mRNA assay (Hologic) Vaginal sample Mini-tampon (wet collection tube)	Aptima HPV mRNA assay (Hologic) Cervical sample Cytobrush (wet collection tube)	Biopsy	77.4% (69.8–85.0%)	77.7% (71.9–83.6%)	Cervical cancer
Adedimeji 2020 (cross-sectional) (160)	Cameroon (public hospital)	High-risk WLHIV, aged 15 to 65 years residing within Akuse community, who were not pregnant at the time of study recruitment.	253	Xpert HPV DNA assay (Cepheid) Vaginal sample Viba-brush (wet collection tube)	Xpert HPV DNA assay (Cepheid) Cervical sample Cervix-brush (wet collection tube)	Not reported	90.70%	87.10%	HPV infection
Awua 2020 (cross-sectional) (161)	Ghana (public hospital and community health posts)	Women from the general population between 25 and 60 years	1,321	Nested L1 PCR HPV DNA assay (Roche) Vaginal sample Viba-brush (wet collection tube)	L1 nested PCR HPV DNA assay (Roche) Cervical sample Cytobrush (wet collection tube)	Cytology	77.20%	63.90%	Cervical cancer
Belinson 2003 (cross-sectional) (162)	China (public hospital)	Women from the general population aged 35 to 50 years	9,183	Digene HC2 HPV DNA assay (Qiagen) Vaginal sample Cytobrush (wet collection tube)	Digene HC2 HPV DNA assay (Qiagen) Endocervical sample Cytobrush (wet collection tube)	Not reported	87.5% (84.2–90.8%)	72.2% (76.2–78.2%)	hrHPV infection, CIN2 and CIN2+
Bhatla 2009 (cross-sectional) (163)	India (hospital, ownership not reporter)	High-risk women presenting with complaints of persistent vaginal discharge, irregular menstrual bleeding, post-coital bleeding, or those found on examination to have an unhealthy cervix	546	Digene HC2 HPV DNA assay (Qiagen) Cervicovaginal sample Brush (wet collection tube)	Digene HC2 HPV DNA assay (Qiagen) Cervical sample Brush (wet collection tube)	Cervical biopsy or conization products	85.00%	98.70%	CIN2+
Bogale 2022 (cross-sectional) (23)	Ethiopia (public hospital)	High-risk WLHIV patients who had an ART follow-up and aged older than 24 years, who volunteered to participate in the study, and signed consent	497	Real-time PCR hrHPV DNA assay (Abbott Molecular) Cervicovaginal sample Cytobrush (wet collection tube)	Real-time PCR hrHPV DNA assay (Abbott Molecular) Cervicovaginal sample Speculum and brush (wet collection tube)	Biopsy	84%	88.4%	hrHPV infection
De Melo Kuil 2017 (cross-sectional) (107)	Brazil (public hospital)	High-risk women aged 18–64 years who were referred to the Colposcopy Ambulatory of the Prevention Department at Barretos Cancer Hospital (Brazil) due to abnormal (atypical squamous cells of uncertain significance or worse) cervical cytology test results (Pap smear)	171	Digene HC2 HPV DNA assay (Qiagen) Vaginal sample Brush (wet collection tube)	Digene HC2 HPV DNA assay (Qiagen) Cervical sample Brush (wet collection tube)	Biopsy	50.0% (27.2–72.8%)	71.7% (61.4–80.6%)	HPV infection and high-grade CIN
Duan 2021 (cross-sectional) (164)	China (public health centre)	Married women from the general population aged 25 to 60 years without a history of CIN or cervical cancer, a hysterectomy that removed the cervix, and any condition that would pose a health risk to the participant	193,490	Sansure real-time PCR HPV DNA assay (Sansure Biotech); Cobas HPV DNA assay (Roche); Digene HC2 HPV DNA assay (Qiagen) Vaginal and cervical sample Brush (wet collection tube)	Sansure real-time PCR HPV DNA assay (Sansure Biotech); Cobas HPV DNA assay (Roche); Digene HC2 HPV DNA assay (Qiagen) Cervical sample Brush (wet collection tube)	Cytology	94.7% (74.0–99.9%)	77.3% (72.6–81.6%)	CIN2+
Eamratsameekool 2023 (cross-sectional) (109)	Thailand (hospital, ownership not reported)	Women from the general population aged 30 to 59 years	535	Cobas PCR HPV DNA assay (Roche) Cervical sample Swab (wet collection tube)	Cobas PCR HPV DNA assay (Roche) Cervical sample Cytobrush (wet collection tube)	Biopsy	81.50%	100%	hrHPV infection
Elliott 2019 (cross-sectional) (165)	Botswana (public hospital)	High-risk WLHIV aged 25 or over presenting for routine appointments at the hospital between March and April 2017	103	Xpert HPV DNA assay (Cepheid) Vaginal sample Flocked swab (wet collection tube)	Xpert HPV DNA assay (Cepheid) Cervical sample Speculum and brush (wet collection tube)	Xpert HPV DNA assay	95.80%	89.90%	hrHPV infection
Esber 2018 (cross-sectional) (112)	Malawi (public health centre)	High-risk women 18 to 49 years of age, spoke Chichewa, had at least one genitourinary symptom, consented to be examined and given biological specimens for testing, and resided in Lilongwe District	199	Xpert HPV DNA assay (Cepheid) Vaginal sample Swab (wet collection tube)	Xpert HPV DNA assay (Cepheid) Cervical sample Brush (wet collection tube)	Biopsy	79% (57.0–93.0%)	99% (95–100%)	hrHPV infection
Flores 2021 (cross-sectional) (22)	Mexico (public health centre)	Sexually active women from the general population aged 30 to 65 with no history of medical or surgical treatment (radiotherapy, chemotherapy, hysterectomy, cone biopsy) for cervical cancer	505	Real-time PCR hrHPV DNA assay; PreTect HPV-proofer mRNA assay (NorChip) Cervicovaginal samples XytoTest device (wet collection tube)	Real-time PCR hrHPV DNA assay; PreTect HPV-proofer mRNA assay (NorChip) Cervical samples Brush (wet collection tube)	Real-time PCR hrHPV DNA assay	46.90%	95.60%	HPV infection
Garcia 2003 (cross-sectional) (166)	Mexico; Peru (public hospital)	High-risk women aged 18 years or older, scheduled for colposcopy examination, and able to provide written informed consent. Women with a history of hysterectomy, current pregnancy, or a history of vaginal trauma or laceration were excluded.	334	Consensus L1 PCR-based RLB HPV DNA assay (Roche) Cervical sample Cytobrush (wet collection tube)	Consensus L1 PCR-based RLB HPV DNA assay (Roche) Cervical Cytobrush (wet collection tube)	Consensus L1 PCR-based RLB HPV DNA assay	49%0.0 (39.2%-58.85)	73% (51.7–79.1%)	High-grade CIN
Haile 2019 (cross-scetional) (118)	Ethiopia (private for-profit hospital)	High-risk WLHIV aged 20 years or older, had an intact uterus, had no history of cervical cancer, were mentally competent, and able and willing to provide informed consent	83	Quantitative PCR HPV DNA assay (RIATOL) Vaginal sample Cytobrush (wet collection tube)	Quantitative PCR HPV DNA assay (RIATOL) Ecto- and endo-cervical sample Cytobrush (wet collection tube)	Quantitative PCR HPV DNA assay	62.50%	87.50%	hrHPV and lrHPV infection
Holanda 2006 (cross-sectional) (167)	Brazil (home-based sampling, testing facility and ownership not reported)	Sexually active women from the general population, aged 15 to 70 years and living in rural districts	878	Digene HC2 HPV DNA assay (Qiagen) Vaginal sample Brush (wet collection tube)	Digene HC2 HPV DNA assay (Qiagen) Ectocervical sample Brush (wet collection tube)	Biopsy	66.7% (55.1–72.3%)	68.7% (65.5–71.9%)	High-risk HPV infection
Jeronimo 2014 (cross-sectional) (168)	India; Nicaragua; Uganda (public health centre and community health posts)	High-risk WLHIV aged 30 to 49 years attending the pilot facilities for routine appointments.	280	careHPV DNA assay (Qiagen) Vaginal and cervical sample Swab (wet collection tube)	careHPV DNA assay (Qiagen) Cervical sample Brush (wet collection tube)	Biopsy	69.6% (63.9–74.9%)	90.0% (90.5–91.4%)	CIN, CIN2+ and CIN3+
Joseph 2021 (cross-sectional) (169)	Zimbabwe (public hospital)	Women from the general population presenting for screening including pregnant women without age restriction, who were randomly selected to participate	253	Aptima HPV mRNA assay (Hologic) Vaginal and cervical sample Flocked swab (wet collection tube)	Aptima HPV mRNA assay (Hologic) Cervical sample Cytobrush (wet collection tube)	Aptima HPV mRNA assay	82.1% (73.9–88.5%)	93.0% (87.1–96.7%)	HPV infection
Kamal 2014 (cross-sectional) (170)	Egypt, Arab Rep. (health centre, ownership not reported)	Sexually active women from the general population 25 to 65 years old, not pregnant, with an intact uterus, and had no history of cervical intraepithelial neoplasia grade 2 or more severe (CIN2+) disease or pelvic radiation	1,601	Digene HC2 HPV DNA assay (Qiagen) Cervical sample Swab (wet collection tube)	Digene HC2 HPV DNA assay (Qiagen) Cervical sample Cytobrush (wet collection tube)	Not reported	89.20%	84.00%	hrHPV infection
Katanga 2021 (cross-sectional) (122)	Tanzania (public and private for-profit hospitals)	High-risk WLHIV in the age group 25–60 years who attended routine cervical cancer screening	464	CareHPV DNA assay (Qiagen) Cervicovaginal sample Evalyn brush (wet collection tube)	CareHPV DNA assay (Qiagen) Cervical sample Brush (wet collection tube)	Biopsy	61.4% (50.4–71.6%)	97.3% (95.2–98.7%)	hrHPV infection
Lack 2005 (cross-sectional) (171)	Gambia, The (facility and ownership not reported)	Women from the general population who had previously been screened for HPV in a reproductive morbidity survey were recruited from a rural area of the Gambia.	377	HPV PCR DNA assay (Qiagen) Vaginal sample Dacron swab tampon (wet collection tube)	HPV PCR DNA assay (Qiagen) Cervical sample Cytobrush (wet collection tube)	Not reported	Swab: 63.9% (51.9–83.3%). Tampon: 72.2% (57.6–86.8%)-	Swab: 93.7% Tampon: 92.5%	HPV infection
Lorenzato 2002 (cross-sectional) (172)	Brazil (public hospital)	Women from the general population aged over 18 years, not pregnant, did not have a history of diagnosed CIN, cervical cancer, or hysterectomy, could accept a pelvic exam mentally and physically	291	Nested L1 PCR HPV DNA assay (Roche) Vaginal and cervical sample Swab (wet collection tube)	Nested L1 PCR HPV DNA assay (Roche) Cervical sample Cytobrush (wet collection tube)	Cytology	79.40%	41.60%	hrHPV infection and CIN3+
Obiri-Yeboah 2017 (cross-sectional) (137)	Ghana (hospital, ownership not reported)	High-risk WLHIV aged 18 years was systematically selected from the list of daily attendants, starting with a randomly picked attendance number for the first woman	194	CareHPV DNA assay (Qiagen) Vaginal sample Cytobrush (wet collection tube)	CareHPV DNA assay (Qiagen) Cervical sample Speculum and cytobrush (wet collection tube)	Not reported	92.6% (85–97.0%)	95.9% (89.8–98.9%)	hrHPV infection
Saidu 2021 (cross-sectional) (173)	Malawi, South Africa (public health centres)	High-risk WLHIV from the general population seeking primary screening and those referred for colposcopy because of abnormal screening test results.	705	Xpert HPV DNA assay (Cepheid) Vaginal sample Swab (wet collection tube)	Xpert HPV DNA assay (Cepheid) Cervical sample Brush (wet collection tube)	Biopsy	HIV negative: 87.7% (80.7–93.0%) HIV positive: 95.8% (91.6–98.3%)	HIV negative: 77.5% (72.8–81.8%) HIV positive: 44.0% (38.0%-5 0.1%)	hrHPV infection
Tiiti 2021 (cross-sectional) (150)	South Africa (public hospital)	Women from the general population who were 18 years and older	527	Real-time PCR hrHPV DNA assay (Abbott); Aptima HPV mRNA assay (Hologic) Vaginal sample Tampon (wet collection tube)	Real-time PCR hrHPV DNA assay (Abbott); Aptima HPV mRNA assay (Hologic) Cervical sample Brush (wet collection tube)	Aptima HPV mRNA assay	DNA: 86.2% mRNA: 68.3%	DNA: 88.0% mRNA: 77.1%	hrHPV infection
Tiiti 2021 (cross-sectional) (174)	South Africa (public hospital)	Women from the general population aged 18 years and older	527	Real-time PCR hrHPV DNA assay (Abbott) Vaginal sample Tampon (wet collection tube)	Real-time PCR hrHPV DNA assay (Abbott) Cervical sample Brush (wet collection tube)	Cytology	86.2% (81.4–89.9%)	87%	hrHPV infection
Toliman 2016 (cross-sectional) (175)	Papua New Guinea (public health centre)	Women from the general population aged 30 to 59 years attending clinics for routine cervical screening were provided information about the study while waiting to be seen	1,005	Xpert HPV DNA assay (Cepheid) Vaginal sample Cytobrush (wet collection tube)	Xpert HPV DNA assay (Cepheid) Cervical sample Cytobrush (wet collection tube)	Xpert HPV DNA assay	90.30%	93.90%	HPV infection
Toliman 2019 (cross-sectional) (176)	Papua New Guinea (health centre, ownership not reported)	Women from the general population aged 30 to 54 years (the target age group for cervical screening in Papua New Guinea)	1,005	Xpert HPV DNA assay (Cepheid) Vaginal sample Cytobrush (wet collection tube)	Xpert HPV DNA assay (Cepheid) Cervical sample Cytobrush (wet collection tube)	Not reported	92.1% (85.1–96.1%)	93.1% (91.1–94.6%)	hrHPV infection
Van De Wijgert 2006 (mixed-methods) (53)	South Africa (public health centre)	Women from the general population aged 18 years or older, sexually active, not pregnant, and willing and able to comply with the study protocol and give written informed consent	450	Digene HC2 HPV DNA assay (Qiagen) Vaginal sample Tampon (wet collection tube)	Digene HC2 HPV DNA assay (Qiagen) Vaginal sample Swab and cytobrush (wet collection tube)	Digene HC2 HPV DNA assay	69.9% (64.0–75.8%)	87.4% (84.1–90.6%)	hrHPV infection
Anand 2022 (cross-sectional) (102)	India (home-based sampling, testing facility and ownership not reported)	Sexually active non-pregnant women from the general population in the age group of 30 to 55 years with no previous history of cervical cancer	485	Digene HC2 HPV DNA assay (Qiagen) Vaginal sample Collection device not reported	Digene HC2 HPV DNA assay (Qiagen) Cervical sample Collection device not reported	Biopsy	59.20%	97.30%	Cervical cancer
Bansil 2015 (cross-sectional) (177)	Uganda (public hospital)	Women from the general population aged 18 years and over who visited the gynaecological outpatient clinics	202	CareHPV DNA assay (Qiagen) Vaginal sample Collection device not reported	CareHPV DNA assay (Qiagen) Cervical Collection device not reported	Cytology	HIV negative: 68.8% (41.3–89.0%) HIV positive: 65.9% (47.8–80.9%)	HIV negative: 92.8% (90.9–94.4%) HIV positive: 78.9% (73.2–83.9%)	HPV infection, CIN2 and CIN2+
Boggan 2015 (cross-sectional) (178)	Haiti (health centre, ownership not reported)	Women from the general population aged between 25 and 65 years who had engaged in vaginal intercourse at least once during their lifetimes	1,845	Digene HC2 HPV DNA assay (Qiagen) Vaginal sample Dacron brush (Collection tube, not described)	Digene HC2 HPV DNA assay (Qiagen) Cervical sample Dacron brush (Collection tube, not described)	Cytology	87.5% (84.1–90.9%)	96.9% (95.1–98.7%)	CIN1, CIN2+ and CIN3+
Chen 2014 (cross-sectional) (179)	China (public hospital)	Women from the general population aged 25–65 years	7,500	Digene HC2 HPV DNA assay (Qiagen) Vaginal sample Collection device, not reported	CareHPV DNA assay (Qiagen) Cervical sample Brush (wet collection tube)	Biopsy	54.60%	89.80%	High risk HPV, CIN2+, CIN3+
Feng 2022 (cross-sectional) (4)	Nigeria (public health centre)	High-risk sexually active women at the ART clinic and outpatient clinic in Lagos, Nigeria	213	Real-time PCR hrHPV DNA assay (Abbott Molecular) Vaginal sample Collection device, not reported	Real-time PCR HPV DNA assay (Sansure Biotech) Cervical sample Collection device, not reported	Not reported	89.8% (77.77–96.60%)	98.21% (94.87–99.63%)	HPV infection
Jones 2007 (cross-sectional) (180)	South Africa (public health centre)	Women from the general population aged 18 years or older, sexually active, self-reportedly not pregnant, and willing to comply with the protocol and gave written informed consent	450	Digene HC2 HPV DNA assay (Qiagen); RBL HPV DNA assay (Roche) Vaginal sample Tampon and swab (wet collection tube)	Digene HC2 HPV DNA assay (Qiagen); RBL HPV DNA assay (Roche) Cervical sample Brush (collection tube, not described)	Digene HC2 HPV DNA assay; RBL HPV DNA assay	Tampon 59.5% (47.9–70.4%) Swab 79.8% (69.6–87.8%)	Tampon 91.8% (86.2–95.7%). Swab 82.6% (75.2–88.5%)	hrHPV infection
Longatto-Filho 2008 (cohort) (128)	Argentina; Brazil (hospitals, ownership not reported)	In our ongoing multi-centre study in Latin America, a cohort of women from the general population has been examined using eight different diagnostic tests as potential screening tools in low-resource settings	1,081	Digene HC2 HPV DNA assay (Qiagen) Vaginal sample Collection device, not reported	Digene HC2 HPV DNA assay (Qiagen) Sampe, not reported Collection device, not reported	Cytology	37.5% (19.5–59.2%)	87.55% (82.7–91.20%)	hrHPV infection
Longatto-Filho 2012 (cohort) (181)	Argentina; Brazil (health centres, ownership not reported)	Women from the general population residing in the cities of Campinas (Brazil), So Paulo (Brazil), Porto Alegre (Brazil), and Buenos Aires (Argentina) were invited to gynaecological consultations and tests examination	4,725	Digene HC2 HPV DNA assay (Qiagen) Cervical sample Tampon (collection tube, not described)	Digene HC2 HPV DNA assay (Qiagen) Cervical sample Swab (collection tube, not described)	Cytology	57.1% (20.2–88.2%)	85.7% (97.7–98.8%)	hrHPV infection and CIN2+
Madhivanan 2021 (cross-sectional) (126)	India (health centre, ownership not reported)	Women from the general population aged 30 years or older who have not undergone cervical cancer screening within the last 3 years and can undergo an informed consent process	120	Digene HC2 HPV DNA assay (Qiagen) Vaginal sample Swab (wet collection tube)	Digene HC2 HPV DNA assay (Qiagen) Cervical Brush (collection tube, not described)	Digene HC2 HPV DNA assay	66.7% (42.8–90.6%)	98.1%; (95.5–100%)	hrHPV infection
Quincy 2012 (cross-sectional) (182)	Nicaragua (public health centre and community health posts)	Women from the general population who were non-pregnant aged 25–60 years with intact uteri, from Leon, Nicaragua	245	Digene HC2 HPV DNA assay (Qiagen) Vaginal sample Swab (collection tube, not described)	Digene HC2 HPV DNA assay (Qiagen) Cervical sample Cytobrush (collection tube not reported)	Histology	78.26%	92.63%	HPV infection
Safaeian 2007 (cohort) (183)	Uganda (public health centre)	High-risk WLHIV attending clinics at the outpatient department of an HIV treatment in Limbe Regional Hospital.	878	Digene HC2 HPV DNA assay (Qiagen) Vaginal sample Swab (wet collection tube)	Digene HC2 HPV DNA assay (Qiagen) Cervical sample Collection device, not reported	Not reported	79.30%	95.30%	HPV infection
Senkomago 2018 (cohort) (184)	Kenya (facility and ownership not reported)	Women from the general population aged at least 18 years, were not in the second or third trimester of pregnancy and had an intact cervix	344	Aptima HPV mRNA assay (Hologic) Cervicovaginal sample Cytobrush (collection tube not described)	Aptima HPV mRNA assay (Hologic) Cervical sample Collection device, not reported	Aptima HPV mRNA assay	79% (56–95%)	74% (69–78%)	hrHPV infection
Sowjanya 2009 (cross-sectional) (185)	India (community health posts, testing facility and ownership not reported)	Women from the general population aged 25 years or older had an intact uterus, were mentally competent, and were able and willing to provide informed consent	432	Digene HC2 HPV DNA assay (Qiagen) Vaginal sample Swab (wet collection tube)	Digene HC2 HPV DNA assay (Qiagen) Cervical sample Swab (collection tube, not described)	Not reported	78.6%	93.7%	hrHPV and lrHPVinfection
Taku 2020 (cross-sectional) (149)	South Africa (public health centre)	High-risk WLHIV aged over 30 years attending the community health clinic for CC screening or other reasons, aged over 18 years with abnormal cervical cytology and CC	737	hpVIR real-time PCR HPV DNA assay (manufacturer, not reported) Vaginal sample Viba brush (wet collection tube)	Digene HC2 HPV DNA assay (Qiagen) Cervical sample Viba brush (Collection tube, not described)	Not reported	88.4%	89.3%	hrHPV infection
Vega Crespo 2022 (cross-sectional) (186)	Ecuador (health centre, ownership not reported)	Sexually active women from the general population aged between 18 and 70 years old; not having undergone an excision or destructive treatment of the cervical intraepithelial neoplasm; not having used vaginal medication at least a week before the examination; not having had sexual intercourse for at least 48 h before the examination; not being pregnant; and the absence of menstrual bleeding at the time of examination	120	13 hrHPV real-time PCR DNA assay (Hybribio) Vaginal sample Evalyn brush (Collection tube, not described)	13 hrHPV real-time PCR DNA assay (Hybribio) Cervical sample Brush (wet collection tube)	13 hrHPV real-time PCR DNA assay	94.4% (74.2–99%)	92.1% (85.2–95.9%)	hrHPV infection
Wong 2018 (cross-sectional) (153)	China (private non-propriety health centre)	Women from the general population aged 18 years or older, not currently pregnant, had no known abnormal Papanicolaou test results, and having presented symptoms of cervical cancer, genital cancer, cervical surgery, or immune treatment of the cervix during the 6 months before recruitment into the study	68	HPV DNA (assay and manufacturer), not reported Vaginal sample Dacron swab (wet collection tube)	HPV DNA (assay and manufacturer), not reported Cervical sample Dacron swab and cytobrush (collection tube, not described)	Not reported	66.70%	66.10%	hrHPV and lrHPVinfection
Wright 2000 (cross-sectional) (187)	South Africa (public health centre)	High-risk WLHIV attending clinics at the outpatient department of an HIV treatment in Limbe Regional Hospital.	878	Digene HC2 HPV DNA assay (Qiagen) Vaginal sample Swab (wet collection tube)	Digene HC2 HPV DNA assay (Qiagen) Cervical sample Brush (dry collection tube)	Cytology	66.1% (51.1–77.8%)	56.20%	HSIL and hrHPV infection

CC, Cervical Cancer; CIN, Cervical Intraepithelial Neoplasia; HSIL, High-Grade Squamous Intraepithelial Lesion; LSIL, Low-Grade Squamous Intraepithelial Lesion; HPV DNA, Human Papilloma Virus Deoxyribonucleic acid, mRNA, messenger Ribonucleic acid; WLHIV, Women living with HIV. ^*^The collection tube contained a liquid medium designed to preserve and transport biological samples, such as cervical cells collected during self- or provider-sampling.

#### 3.3.3 Cost

Most studies of the seven reporting on costs (*n* = 5, 71.4%) focused on the expenditures associated with either self- or provider-sampling (Table 3). Laboratory processing costs (associated with sample processing and analysis) were identical [$5.75 (United States dollars)] for both self and provider-collected samples (59). However, another study estimated a higher laboratory processing cost for the provider-collected samples ($7.10) (60). The round-trip transportation cost for provider-sampling was $0.76 (60). The mean cost per woman screened was slightly lower for self-sampling [$37.1 (range $27.6–$54.0)] compared to provider-sampling [$37.7 (range $26.4–$52.0)] (61). The self-sampling test kit’s reported price range was $11.24–$14.15 (62, 63). For the clinician’s time, the cost was $20.06 for provider-sampling (63). Self-sampling had a lower final aggregated direct medical cost (all expenses incurred throughout the testing process—overall costs) of $7.49 compared to $7.95 for provider-sampling (59).

**Table 3:** Cost of HPV self-sampling and provider-sampling.

Author, year	Country, income bracket (area) WHO region UN sub-region (intermediate)	Health setting	Population	Self-sampling Assay (manufacturer) Sample type Collection device	Provider-sampling Assay (manufacturer) Sample type Collection device	Self-sampling related costs	Provider-sampling-related costs
Cost measures							
Shi 2012 (59) (Micro-costing)	China, UMIC (rural) Western Pacific Eastern Asia (unclassified)	Public hospital	Women aged 30–59 years attending the Women and Children’s Hospital in Xiang yuan	HPV DNA (Assay, sample type and collection device, not reported)	Assay, sample type and collection device, not reported	Laboratory processing costs, inclusive of the test kit cost: 5.75 USD; Final aggregated direct medical cost for self-sampling: 7.49 USD	Laboratory processing costs, inclusive of the test kit cost: 5.75 USD; Final aggregated direct medical cost for self-sampling: 7.95 USD
Campos 2019 (60) (Modelling)	El Salvador, LMIC (area, not reported) Americas Latin America and the Caribbean (Central America)	Health setting, not reported	Data from phase 2 of the CAPE demonstration (*n* = 8,000 women) were used to inform a mathematical model of HPV infection and cervical cancer	Assay, sample type and collection device, not reported	Assay, sample type and collection device, not reported	Not reported	Laboratory processing cost; 7.10 USD; Transportation (round trip, clinic): 0.76 USD
Olwanda 2020 (61) (Micro-costing)	Kenya, LMIC (rural) African Sub-Saharan Africa (Eastern Africa)	Community health posts (testing facility and ownership, not reported)	Women aged between 25 and 65 years who did not screen at the CHCs were offered home-based screening in November 2018	CareHPV DNA assay (Qiagen) Vaginal sample Brush (wet collection tube)	CareHPV DNA assay (Qiagen) Vaginal sample Brush (wet collection tube)	The mean cost per woman screened by self-sampling was 37.1 USD (range 27.6–54.0)	The mean cost per woman screened by the provider is 37.7 USD (range 26.4–52.0)
Abdullah 2018 (62) (Cross-sectional)	Malaysia, UMIC (area, not reported) Western Pacific South-Eastern Asia (Unclassified)	Public health centre	Married women aged 28 to 60 years old	Digene HC2 HPV DNA assay (Qiagen) Vaginal sample Brush (collection tube, not described)	Digene HC2 HPV DNA assay (Qiagen) Vaginal sample Brush (collection tube, not described)	The price of a kit is 11.24 USD. Obtaining directly from the supplier in a governmental clinic may be cheaper compared to the private sector, where profit has been the priority	Not reported
Flores 2011 (63) (Modelling)	Mexico UMIC (area, not reported) Americas Latin America and the Caribbean (Central America)	Health setting, not reported	Female clients from the Mexican Institute of Social Security, between the ages of 20 to80 years, in the state of Morelos	HPV DNA (Assay, sample type and collection device, not reported)	Assay, sample type and collection device, not reported	Test kit price: 14.15 USD	Cost for clinician’s time: 20.06 USD
Cost-effectiveness measures							
Campos 2017 (64) (Modelling)	Uganda, LIC (area, not reported) African Sub-Saharan Africa (Eastern Africa)	Community health posts and public health centre	Women aged 30 to 49 years	HPV DNA (Assay, sample type and collection device, not reported)	Assay, sample type and collection device, not reported	Self-collection campaigns cost 70 international dollars per YLS, which is very cost-effective as ICERs are below 1,690 international dollars’ *per capita* GDP	Clinic-based provider collection was associated with a slightly higher ICER (140 international dollars per YLS);
Zhao 2023 (65) (Modelling)	China, UMIC (urban; rural) Western Pacific Eastern Asia (Unclassified)	Health setting, not reported	Women aged 30 to 65 years in urban and rural China, with a 5-year screening interval	HPV DNA (Assay, sample type and collection device, not reported)	No comparison test reported	Self-collected HPV test without triage is regarded as the optimal strategy with the most incremental QALYs gained (420 QALYs; ICER = −1401.7 USD per QALY)	Not reported

LIC, Low-income country; LMIC, Low-middle-income country; UMIC, Upper-middle-income country.

Two studies (28.6%) reported on the following cost-effectiveness measures: incremental quality-adjusted life years (QALYs) and total cost-effectiveness ratio (ICER) per year of life saved (YLS) (64, 65). Self-sampling was associated with lower costs of 70 international dollars per YLS, with ICERs below 1,690 international dollars (64). Compared with current strategies (physician-HPV with genotype or cytology triage), all screen-and-treat strategies were cost-effective and self-HPV without triage was optimal with the most incremental QALYs gained (220–440) (65).

#### 3.3.4 Uptake

Four randomised studies of the seven reporting on uptake (57.1%) reported that the uptake of HPV self-sampling ranged between 84.1 and 99.2% (Table 4) (66–69). Test uptake was measured based on participants’ consent to the sampling method offered within randomised studies. Participants randomised to self-sampling were 4.02 times more likely to consent than those in the provider-sampling group (95% CI: 3.44–4.71) (69). The remaining three studies (42.9%) offered participants a choice between self and provider-sampling (52, 54, 70). Where a choice was offered (*n* = 3), uptake of self-sampling ranged between (52.7–96%) in comparison to where participants were randomised (*n* = 4) (84.5–99%).

**Table 4:** Uptake of HPV self-sampling.

Author, Year (Design)	Country, income bracket (area) WHO region UN sub-region (intermediate)	Health setting	Population	Self-sampling Assay (manufacturer) Sample type Collection device	Provider-sampling Assay (manufacturer) Sample type Collection device	Self-sampling		The proportion of women taking HPV testing by provider-sampling
						The proportion of women taking HPV testing by self-sampling	Effect measure	
Arrossi 2015 (69) (Cluster RCT)	Argentina, UMIC (rural) Americas Latin America and the Caribbean (South America)	Community health posts and public health centre.	Women 30 years or older living in a household visited by community health workers	HPV DNA (Assay, not reported) Vaginal sample Brush (wet collection tube)	Assay, sample type and collection device, not reported	86.0% (2,618/3049), *P* < 0.0001	Risk ratio; 4.02 (95% CI: 3.44–4.71)	20.0 (599/2964)
Arrossi 2017 (70) (Cross-sectional)	Argentina, UMIC (area, not reported) Americas Latin America and the Caribbean (South America)	Home-based sampling, testing facility and ownership not reported	Women aged 30 years and above (home-based screening)	HPV DNA (Assay and sample type, not reported) Brush (collection tube, not described)	Assay, sample type and collection device, not reported	52.7% (2,983/5657)	Measure of effect not reported	47.3% (2,674/5657)
Gizaw 2019 (68) (Cluster RCT)	Ethiopia, LIC (urban; rural) African Sub-Saharan Africa (Eastern Africa)	Public hospital	Non-screened women attending a healthcare facility	HPV DNA (Assay, not reported) Cervical sample Brush (dry collection tube)	(Assay, not reported) Cervical sample Collection device, not reported	84.1% (1,020/1213), *P* < 0.0001	Measure of effect not reported	50.5% (575/1143), *P* < 0.0001
Behnke 2020 (54) (Mixed-methods)	Ghana, LMIC (rural) African Sub-Saharan Africa (Western Africa)	Private non-propriety health centre	Women aged 30 to 65 years, living or working in Karu	HPV DNA (Assay, sample type and collection device, not reported)	Assay, sample type and collection device, not reported	96% (50/52)	Measure of effect not reported	2% (1/52)
Bansil 2014 (52) (Mixed-methods)	India and Nicaragua, LMICs; Uganda LIC (area, not reported) South-East Asia; Americas; African Southern Asia (unclassified); Latin America and the Caribbean (Central America); Sub-Saharan Africa (Eastern Africa)	Home-based and public health centre	Female staff members	CareHPV DNA assay (Qiagen) Vaginal sample Brush (collection tube, not described)	CareHPV DNA assay (Qiagen) Cervical sample Brush and speculum (collection tube, not described)	89.7% (3,474/3873)	Measure of effect not reported	10.3% (399/3873)
Modibbo 2017 (67) (Individual RCT)	Nigeria, LMIC (peri-urban) African Sub-Saharan Africa (Western Africa)	Community health posts and public hospital	Married women aged 28 to 60 years.	HPV DNA (Assay, not reported) Cervicovaginal sample Swab (dry collection tube)	HPV DNA (Assay, not reported) Cervicovaginal sample Swab (dry collection tube)	93% (185/200), *P* < 0.001	Measure of effect not reported	56% (113/200), *P* < 0.001
Moses 2015 (66) (Individual RCT)	Uganda, LIC (area, not reported) African Sub-Saharan Africa (Eastern Africa)	Community health posts and public hospitals and health centres	Women aged 30 to 65 years attending a health care facility and community-based screening	HPV DNA (Assay, not reported) Vaginal sample Swab (dry collection tube)	Assay, sample type and collection device, not reported	99.2% (248/250) *P* < 0.001	Measure of effect not reported	48.4% (121/250), *P* < 0.001

CI, Confidence Interval; P, Probability value; LIC, Low-income country; LMIC, Low-middle-income country; UMIC, Upper-middle-income country.

#### 3.3.5 Health equity factors

**Figure 2:**
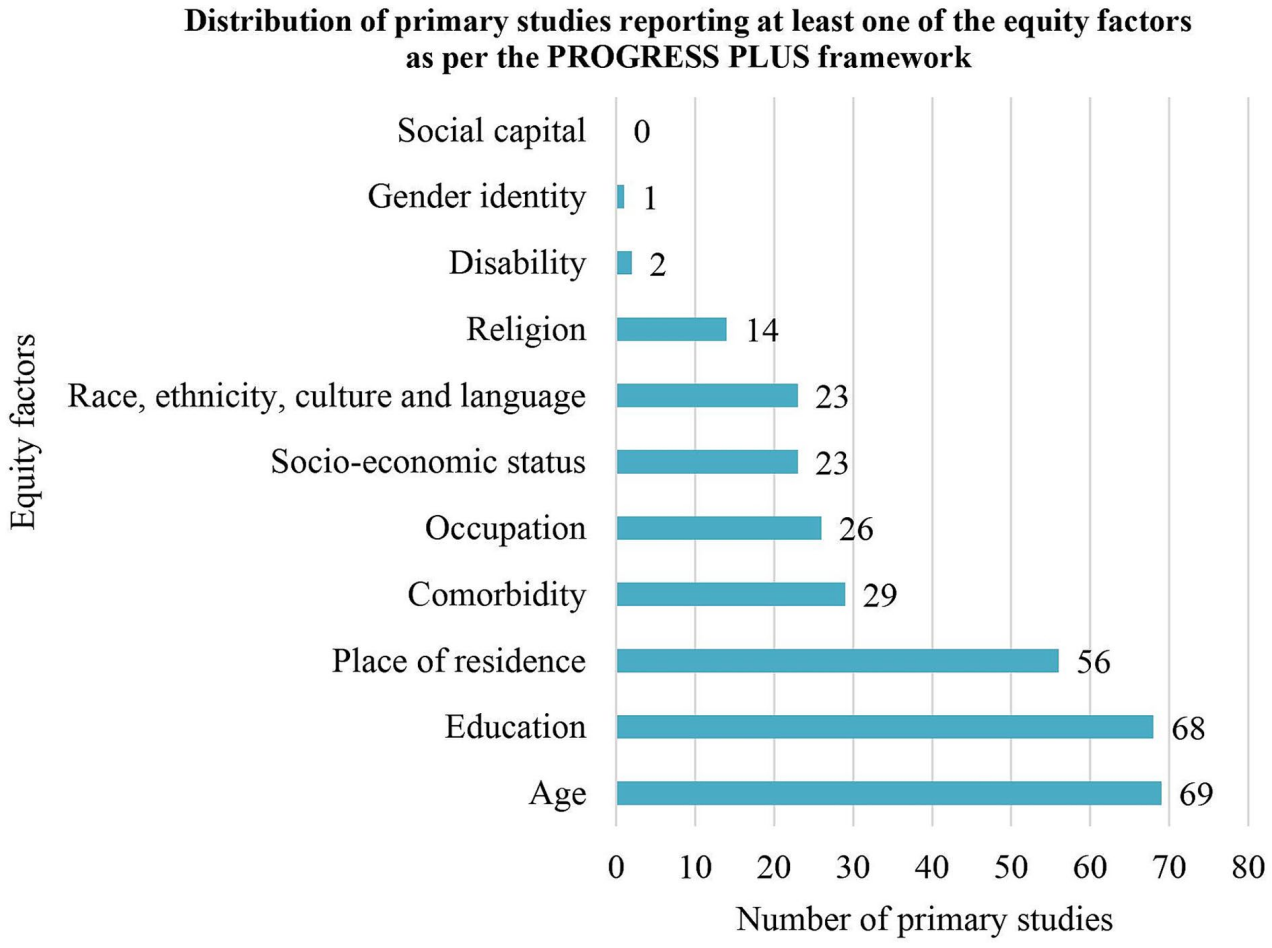
Distribution of studies reporting at least one of the equity factors as per the PROGRESS PLUS framework.

Studies mentioned 10 of the 11 PROGRESS-Plus health-important equity factors but none of them evaluated their impacts on HPV self-sampling (Figure 2 and Supplementary Annex 8) (40). No study mentioned social capital. The most frequently mentioned equity factors were age (*n* = 69, 65.1%), education (*n* = 68, 64.2%) and place of residence (*n* = 59, 55.6%). The least mentioned equity factors were disability (*n* = 2, 1.9%) and gender identity (*n* = 1, 0.9%) (69, 71, 72).

### 3.4 Quality appraisal

**Figure 3:**
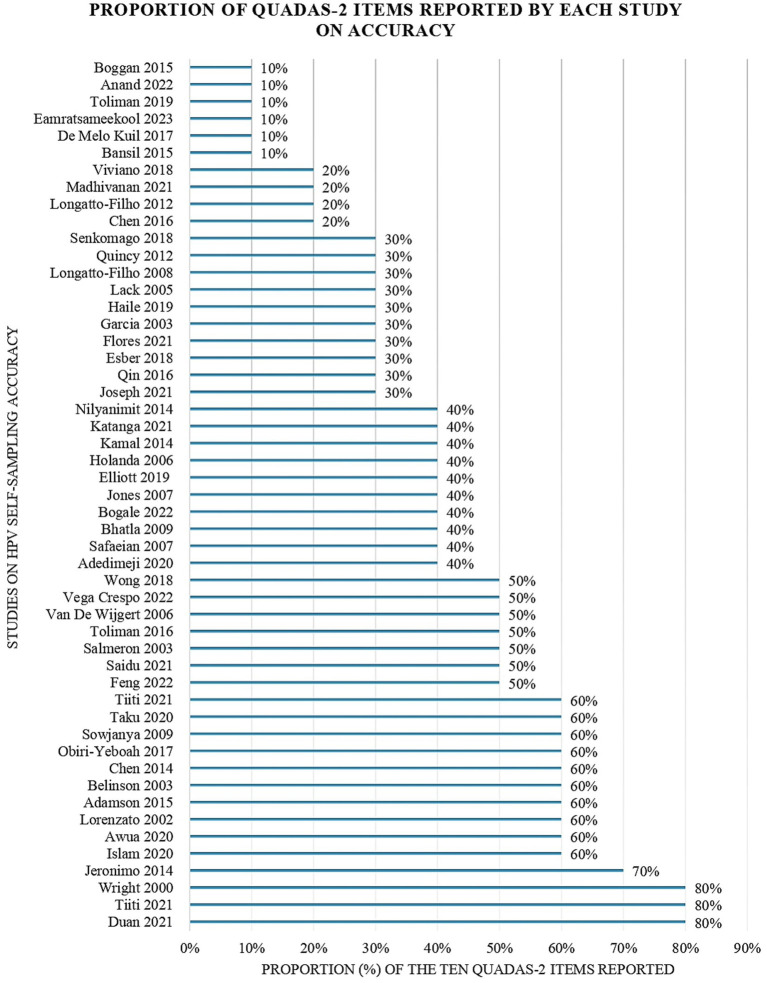
Proportion of QUADAS-2 items reported by each study evaluating the accuracy of HPV self-sampling.

Quality appraisal findings of the included accuracy studies using the QUADAS-2 tool are available in Figure 3 (43). Most accuracy studies reported an appropriate interval between the index and the reference test (*n* = 40, 78.4%) and that all patients received the same reference standard (*n* = 31, 60.8%). Most of the accuracy studies avoided inappropriate exclusions (*n* = 28, 54.9%) case–control designs (*n* = 24, 47.1%) and used suitable reference standards (*n* = 26, 50.9%). However, only (*n* = 22, 43.1%) studies included all patients in their analyses. Few studies (*n* = 16, 31.3%) adopted random or consecutive sampling and (*n* = 12, 23.5%) used pre-specified thresholds. Fewer studies (*n* = 6, 11.8%) blinded the interpretation of the reference and the index (*n* = 5, 9.8%) test results.

## 4 Discussion

### 4.1 Summary of the key findings

In this scoping review, we mapped and summarised the existing evidence and identified gaps in HPV self-sampling vs. provider-sampling for cervical cancer screening in LMICs. The majority/most studies were from sub-Saharan Africa and upper-middle-income countries indicating minimal evidence from other LMIC settings such as South Asia, Southeast Asia and Latin America. Participants included had a wide age range (15–88 years). WHO recommends HPV screening at 30 years of age for non-HIV-infected individuals and 25 years for persons living with HIV with regular screening after every 5–10 years (73). While most accuracy studies used wet sample transportation modes, few included dry sample transportation modes. Direct comparisons were limited to one study, which suggested that dry sample transportation performed similarly to wet sample transportation for detecting high-grade squamous intraepithelial lesions (HSIL) (58). Our findings revealed HPV self-sampling’s importance for improving cervical cancer screening uptake and highlighted the need for further research on equity considerations (66–69). Most studies found HPV self-sampling highly acceptable although the reported ranges were broad (46–52). Laboratory processing costs were similar, but final aggregated costs were lower for self-collected samples (59). Most studies reported higher uptake with self-collected samples when blind randomisation of participants took place (66–69). Regarding equity factors, most studies mentioned place of residence, education, and age while few mentioned disabilities, and only one reported on gender identity (69, 71, 72).

### 4.2 Comparisons with other studies

Similar to our findings on the acceptability of self-sampling, Morgan et al. (74) reported wide ranges (64.7–93.0%). The reported wide ranges could be attributed to variations in sample sizes across studies, study populations, cultural differences and the settings in which the studies were conducted (74). In our study, these variations could reflect underlying social and cultural dynamics, especially in LMICs, where health-seeking behaviours and perceptions of reproductive health are shaped by societal norms and access to healthcare. For instance, cultural taboos and embarrassment around cervical cancer screening may explain the lower acceptability rates that were reported in certain settings. Additionally, Camara et al. (75) also reported that most women favoured self-sampling to evade feelings of embarrassment, although some opted for provider-sampling because of issues related to trust and self-confidence. These findings suggest that trust in the healthcare system and self-efficacy in performing medical procedures play crucial roles in screening uptake. Efforts to build trust and increase confidence in self-sampling could therefore help address these challenges. Other barriers like limited access to healthcare, religious and cultural beliefs, and time constraints due to daily responsibilities are important factors that contribute to lower acceptance rates of self-sampling among women (16, 17, 30, 74, 76–80). These barriers not only affect acceptability but also the feasibility of scaling up HPV self-sampling programs in regions where healthcare resources are scarce. Addressing these factors is critical for improving uptake and ensuring the sustainability of self-sampling initiatives in such settings.

In accordance with our results, three systematic reviews encompassing studies done in Africa and another review of studies done in Asia reported higher sensitivities and specificities of self-sampling (81–84). Two of these reported higher sensitivities for CIN2+ but low specificities for CIN2+ (82, 84). Sy et al. (83), assessed the accuracy of self-sampling compared to provider-sampling, including 21 studies for meta-analysis. They reported sensitivities close to 80% and specificities close to 90% of self-sampling to detect high-risk HPV in reference to provider-sampling (83). They also investigated inter-study heterogeneity, finding variabilities in populations, settings and testing methods, likewise our study (83). Our findings revealed that the sensitivity and specificity of self-sampling ranged from 37.5 to 96.8% and 41.6 to 100.0%, respectively. However, the wide range of accuracy measures presented in this review could be attributed to variations in factors such as the sampled population, transportation method, sample-collection device and sample type across included studies (83). These variations underscore the importance of standardising protocols and ensuring reliable sample transport, especially in LMICs, where infrastructure challenges may affect diagnostic accuracy. Self-sampling using dry collection devices was comparable to provider-sampling using wet collection devices for the detection of HSIL (58). While promising, these findings would require further validation as Islam et al. (58) were limited in sample size (400 participants). Additionally, despite the good quality of this study (by reporting 60% of the QUADAS II tool items) interpretations regarding these findings should be made with caution.

Our findings, indicating that HPV self-sampling yielded population coverage gains over provider-sampling and increased cervical cancer screening attendance because of its cost-effectiveness, align with three other systematic reviews focusing on the cost and effectiveness of HPV self-sampling (30, 31, 85). However, evidence on the cost-effectiveness of HPV self-sampling was limited to only two studies (64, 65). While our findings suggest that self-sampling is generally more cost-effective than provider-sampling, the cost-effectiveness of HPV self-sampling in LMICs remains highly context-dependent. Differences in healthcare infrastructure, resource allocation, and local economic factors can significantly affect costs, such as transportation and clinician time, which were shown to vary across studies. Its cost-effectiveness should be evaluated within the specific economic and healthcare contexts of individual regions, acknowledging the uncertainties and limitations that may affect its broader applicability.

The uptake of cervical cancer screening in low and middle-income countries (LMICs) increased when women were offered self-sampling options (30, 86–88). In a scoping review comprising 27 studies, Serrano et al. (88) found that, as of 2022, only a few countries globally (*n* = 17, 12%) recommended HPV self-sampling. This finding aligns with ours, as most of the evidence on self-sampling uptake originated from just seven countries (Argentina, Ethiopia, Ghana, India, Nicaragua, Nigeria and Uganda). Uptake of self-sampling was high when participants were randomised, but when they had a choice between self- and provider-sampling, there was a large range of uptake reported across studies. This variability could be attributed to differences in sample sizes between randomised studies (200–3,049 participants) and non-randomised studies (52 to 5,657 participants). In a meta-analysis that included 26 high-income countries (HICs), Yeh et al. (86) also reported that uptake was two times higher among participants randomised. Understanding these underlying mechanisms and tailoring strategies accordingly could enhance the effectiveness of self-sampling interventions within LMICs.

Six WHO guidelines published between 2014 and 2020 recommended HPV self-sampling on women aged 30–60 years (89–94). Two of these strongly advocated for HPV self-sampling for DNA testing (89, 92). However, concerning HPV mRNA testing, the guidelines acknowledged that the available low-certainty evidence suggested an inferior performance of self-samples (90). Therefore, provider sampling is recommended for mRNA testing (91). Further studies comparing the outcomes between self-sampling and provider-sampling strategies for DNA and mRNA testing are essential to addressing this gap.

### 4.3 Gaps and implications for practice, policy and future research

Ease of use may necessitate increased uptake of HPV self-sampling (20). More research on the effects of the population sampled, transportation mode, type of swabs, type of samples and cadres of providers on test accuracy would inform decision-making on the uptake, especially with the noted variation in test sensitivity and specificity. Reference tests, comparator tests, type of tests (mRNA vs. DNA), and sampling devices vary in studies requiring more research on their effects on the test accuracy of HPV self-sampling across multiple settings. It is imperative to conduct a systematic review and meta-analysis that compares the accuracy and acceptability of self-sampling in LMICs versus high-income countries. This will help identify gaps in evidence and provide crucial guidance for implementation decisions. While one study showcased that there are no differences in the accuracy of self-sampling depending on the transportation mode, interpretations should be drawn with caution (58). Further research is necessary to confirm these findings and enhance generalizability.

Examining the impact of health equity factors on self-sampling is crucial for shaping policy and reducing disparities (95). Policymakers can utilise the evidence provided to guide policy and guideline development, allocate resources and plan implementation strategies. Therefore, studies may incorporate health equity factors within their evaluations—encompassing participants’ characteristics, future relationships with other settings, and time-dependent relationships (96). Adhering to reporting guidelines on health equity, such as those available on the Equator Network, can improve the quality of implementation studies on HPV self-sampling and enhance applicability (97).

### 4.4 Strengths and limitations

The strength of our review included conducting an extensive search of the literature, minimising bias by having two review authors conduct study selection independently and having two senior reviewers conduct quality control by double-checking all excluded studies. Studies reporting on the diagnostic accuracy of HPV self-sampling were also subjected to quality assessment by checking on the reporting of the 10 items across the four domains of the QUADAS-2 tool. Our review also gives an overall view of all relevant factors for any diagnostic intervention with the public health lens. Included studies utilized data from a range of contexts including small pilot studies such as community-based initiatives and local screening programs encompassing multiple regions within the respective nations. This review provides an overview of all critical pieces (accuracy, acceptability, cost, uptake and equity) of self-sampling needed for policy recommendation at both national and global levels.

One of the limitations of our review was that data extraction for each study was done by a single reviewer independently and not by two reviewers independently. Studies that compared the sensitivity and specificity of dry vs. wet transportation modes were limited. While our search terms were kept broad, our review could have missed a few studies, especially those focusing on equity concepts using other related terms; however, we mitigated that by searching the reference lists of relevant secondary studies. Since the scope of this review was broad, differences in study populations, concepts, outcomes and methodologies within included studies may have limited the comparisons across studies.

## 5 Conclusion

The acceptability of HPV self-sampling was high across most studies, with 80.3% of participants willing to perform the procedure themselves, citing increased privacy, convenience, and autonomy as key motivators. However, barriers such as concerns over proper self-sampling technique, fear of discomfort, and cultural or religious beliefs influenced the preferences of some participants, highlighting the need for targeted interventions to address these concerns. However, this review showed that self-sampling tests varied widely in how accurately they detected HPV. Transportation of self-collected vaginal samples using swabs stored in liquid media incurs additional costs and might create testing barriers for women. Dry transport of samples has the advantage of lower cost and ease of handling. Evidence on comparisons between self-collected vaginal samples using the dry swab and those transported in liquid media is limited, particularly in LMICs. The evidence on cost-effectiveness varies across regions, and further research is needed to determine its broader applicability and address context-specific limitations. More research evaluating variable outcomes for accuracy and uptake, comparisons of transportation modes and comparisons with high-income countries will effectively inform cervical screening uptake. Impact evaluations of health equity factors on HPV self-sampling can improve the applicability and guide the development of policies and programmes.

## Data Availability

The original contributions presented in the study are included in the article/Supplementary material, further inquiries can be directed to the corresponding author.

## Footnotes

^1^ https://doi.org/10.17605/OSF.IO/34TUY

^2^IRIS Home (who.int).

^3^Search | International AIDS Society (IAS) (iasociety.org).

^4^IARC Publications Website—IARC Biennial Reports

^5^Dry self- or provider sampling: Method of sample collection by either the patient or the healthcare professional without the use of liquid preservatives or transport media.

^6^Wet self- or provider sampling: Method of sample collection by either the patient or the healthcare professional typically using transport media or liquid preservatives to maintain the sample’s integrity until it reaches the laboratory for analysis.
