# Supplementary material for: Human papillomavirus self-sampling versus provider-sampling in low- and middle-income countries: a scoping review of accuracy, acceptability, cost, uptake, and equity
Source: Front Public Health. 2024 Nov 29;12:1439164. doi: 10.3389/fpubh.2024.1439164 (PMC11638174; doi:10.3389/fpubh.2024.1439164)
Supplement: Supplementary file 2 [file Table_2.docx]

# **Annex 2: Country Classification**

## **By Income (World Bank Classification for the fiscal year 2023/2024^[[1]](#footnote-1)^)**

Low-income economies [n=26]

| Afghanistan | Korea, Dem. People's Rep | South Sudan |
| --- | --- | --- |
| Burkina Faso | Liberia | Sudan |
| Burundi | Madagascar | Syrian Arab Republic |
| Central African Republic | Malawi | Togo |
| Chad | Mali | Uganda |
| Congo, Dem. Rep | Mozambique | Yemen, Rep. |
| Eritrea | Niger |  |
| Ethiopia | Rwanda |  |
| Gambia, The | Sierra Leone |  |
| Guinea-Bissau | Somalia |  |

Lower-middle income economies [n=54]

| Angola | Jordan | Philippines |
| --- | --- | --- |
| Algeria | India | Samoa |
| Bangladesh | Iran, Islamic Rep | São Tomé and Principe |
| Benin | Kenya | Senegal |
| Bhutan | Kiribati | Solomon Islands |
| Bolivia | Kyrgyz Republic | Sri Lanka |
| Cabo Verde | Lao PDR | Tanzania |
| Cambodia | Lebanon | Tajikistan |
| Cameroon | Lesotho | Timor-Leste |
| Comoros | Mauritania | Tunisia |
| Congo, Rep. | Micronesia, Fed. Sts. | Ukraine |
| Côte d'Ivoire | Mongolia | Uzbekistan |
| Djibouti | Morocco | Vanuatu |
| Egypt, Arab Rep. | Myanmar | Vietnam |
| Eswatini | Nepal | Zambia |
| Ghana | Nicaragua | Zimbabwe |
| Guinea | Nigeria |  |
| Haiti | Pakistan |  |
| Honduras | Papua New Guinea |  |

Upper-middle-income economies [n=54]

| Albania | Fiji | North Macedonia |
| --- | --- | --- |
| Argentina | Gabon | Palau |
| Armenia | Georgia | Paraguay |
| Azerbaijan | Grenada | Peru |
| Belarus | Guatemala | Russian Federation |
| Belize | Indonesia | Serbia |
| Bosnia and Herzegovina | Iraq | South Africa |
| Botswana | Jamaica | St. Lucia |
| Brazil | Kazakhstan | St. Vincent and the Grenadines |
| Bulgaria | Kosovo | Suriname |
| China | Libya | Thailand |
| Colombia | Malaysia | Tonga |
| Costa Rica | Maldives | Türkiye |
| Cuba | Marshall Islands | Turkmenistan |
| Dominica | Mauritius | Tuvalu |
| Dominican Republic | Mexico | West Bank and Gaza |
| El Salvador | Moldova |  |
| Equatorial Guinea | Montenegro |  |
| Ecuador | Namibia |  |

High-income economies [n=83]

| American Samoa | Germany | Oman |
| --- | --- | --- |
| Andorra | Gibraltar | Panama |
| Antigua and Barbuda | Greece | Poland |
| Aruba | Greenland | Portugal |
| Australia | Guam | Puerto Rico |
| Austria | Hong Kong SAR, China | Qatar |
| Bahamas, The | Hungary | Romania |
| Bahrain | Iceland | San Marino |
| Barbados | Ireland | Saudi Arabia |
| Belgium | Isle of Man | Seychelles |
| Bermuda | Israel | Singapore |
| British Virgin Islands | Italy | Sint Maarten (Dutch part) |
| Brunei Darussalam | Japan | Slovak Republic |
| Canada | Korea, Rep. | Slovenia |
| Cayman Islands | Kuwait | Spain |
| Channel Islands | Latvia | St. Kitts and Nevis |
| Chile | Liechtenstein | St. Martin (French part) |
| Croatia | Lithuania | Sweden |
| Curaçao | Luxembourg | Switzerland |
| Cyprus | Macao SAR, China | Taiwan, China |
| Czech Republic | Malta | Trinidad and Tobago |
| Denmark | Monaco | Turks and Caicos Islands |
| Estonia | Nauru | United Arab Emirates |
| Faroe Islands | Netherlands | United Kingdom |
| Finland | New Caledonia | United States |
| France | New Zealand | Uruguay |
| French Polynesia | Northern Mariana Islands | Virgin Islands (U.S.) |
| Guyana | Norway |  |

## By WHO region

# WHO African Region

Algeria, Angola, Benin, Botswana, Burkina Faso, Burundi, Cabo Verde, Cameroon, Central African Republic, Chad, Comoros, Congo, Côte d'Ivoire, Democratic Republic of the Congo, Equatorial Guinea, Eritrea, Eswatini, Ethiopia, Gabon, Gambia, Ghana, Guinea, Guinea-Bissau, Kenya, Lesotho, Liberia, Madagascar, Malawi, Mali, Mauritania, Mauritius, Mozambique, Namibia, Niger, Nigeria, Rwanda, Sao Tome and Principe, Senegal, Seychelles, Sierra Leone, South Africa, South Sudan, Togo, Uganda, United Republic of Tanzania, Zambia, Zimbabwe.

# WHO Region of the Americas

Antigua and Barbuda, Argentina, Bahamas, Barbados, Belize, Bolivia (Plurinational State of), Brazil, Canada, Chile, Colombia, Costa Rica, Cuba, Dominica, Dominican Republic, Ecuador, El Salvador, Grenada, Guatemala, Guyana, Haiti, Honduras, Jamaica, Mexico, Nicaragua, Panama, Paraguay, Peru, Puerto Rico (*Associate WHO Member State), Saint Kitts and Nevis, Saint Lucia, Saint Vincent and the Grenadines, Suriname, Trinidad and Tobago, United States of America, Uruguay, Venezuela (Bolivarian Republic of).

# WHO South-East Asia Region

Bangladesh, Bhutan, Democratic People's Republic of Korea, India, Indonesia, Maldives, Myanmar, Nepal, Sri Lanka, Thailand, Timor-Leste.

# WHO European Region

Albania, Andorra, Armenia, Austria, Azerbaijan, Belarus, Belgium, Bosnia and Herzegovina, Bulgaria, Croatia, Cyprus, Czechia, Denmark, Estonia, Finland, France, Georgia, Germany, Greece, Hungary, Iceland, Ireland, Israel, Italy, Kazakhstan, Kyrgyzstan, Latvia, Lithuania, Luxembourg, Malta, Monaco, Montenegro, Netherlands, North Macedonia, Norway, Poland, Portugal, Republic of Moldova, Romania, Russian Federation, San Marino, Serbia, Slovakia, Slovenia, Spain, Sweden, Switzerland, Tajikistan, Türkiye, Turkmenistan, Ukraine, United Kingdom of Great Britain and Northern Ireland, Uzbekistan.

# WHO Eastern Mediterranean Region

Afghanistan, Bahrain, Djibouti, Egypt, Iran (Islamic Republic of), Iraq, Jordan, Kuwait, Lebanon, Libya, Morocco, Oman, Pakistan, Qatar, Saudi Arabia, Somalia, Sudan, Syrian Arab Republic, Tunisia, United Arab Emirates, West Bank and Gaza Strip (*Non-Member area), Yemen.

# WHO Western Pacific Region

Australia, Brunei Darussalam, Cambodia, China, Cook Islands, Fiji, Japan, Kiribati, Lao People's Democratic Republic, Malaysia, Marshall Islands, Micronesia (Federated States of), Mongolia, Nauru, New Zealand, Niue, Palau, Papua New Guinea, Philippines, Republic of Korea, Samoa, Singapore, Solomon Islands, Tokelau (*Associate WHO Member State), Tonga, Tuvalu, Vanuatu, Viet Nam.

1. [World Bank income groups, 2023 (ourworldindata.org)](https://ourworldindata.org/grapher/world-bank-income-groups) [↑](#footnote-ref-1)
