# Supplementary material for: Human papillomavirus self-sampling versus provider-sampling in low- and middle-income countries: a scoping review of accuracy, acceptability, cost, uptake, and equity
Source: Front Public Health. 2024 Nov 29;12:1439164. doi: 10.3389/fpubh.2024.1439164 (PMC11638174; doi:10.3389/fpubh.2024.1439164)
Supplement: Supplementary file 3 [file Table_3.docx]

# **Annex 3: Full Search Strategy**

## Number of Results Obtained

| Serial No. | Database Name | No. of Results |
| --- | --- | --- |
|  | CINAHL (EBSCOHost) | 705 |
|  | Clinicaltrials.gov | 122 |
|  | Cochrane CENTRAL | 210 |
|  | Embase (OVID) | 670 |
|  | Global Index Medicus | 45 |
|  | Medline (OVID) | 460 |
|  | MedRxiv | 57 |
|  | Science Citation-Expanded (Web of Science) | 1047 |
|  | Scopus (Elsevier) | 249 |
|  | WHO International Clinical Trials Registry Platform (ICTRP) | 88 |
|  | WHO Website | 38 |
|  | International Agency for Research on Cancer (IARC) | 4 |
|  | International AIDS Society | 6 |
|  | Citation Searching (Reference lists of included systematic reviews) | 38 |
|  | **Total number of results** | **3739** |
|  | **Number of duplicates removed** | **1179** |
|  | **Number of records screened** | **2560** |
|  | **Number of records included** | **124** |

## Search Histories

**#1 Ovid MEDLINE(R) ALL <1946 to July 06, 2023>**

| 1 | human papillomavirus.mp. or Human Papillomavirus Viruses/ |
| --- | --- |
| 2 | HPV.ti,ab. |
| 3 | Uterine Cervical Dysplasia/ |
| 4 | Uterine Cervical Neoplasms/ |
| 5 | ((cervix or cervical or cervic*) and (cancer or carcinoma or neoplas* or dysplas* or squamous)).ti,ab. |
| 6 | Papillomavirus Infections/ or Papillomaviridae/ |
| 7 | 1 or 2 or 3 or 4 or 5 or 6 |
| 8 | diagnostic test.mp. or Diagnostic Tests, Routine/ |
| 9 | HPV test*.mp. |
| 10 | HPV screening.mp. |
| 11 | HC2.mp. |
| 12 | hybrid capture.ti,ab. |
| 13 | 8 or 9 or 10 or 11 or 12 |
| 14 | 7 and 13 |
| 15 | (afghan* or africa* or albania* or algeria* or angola* or antigua* or barbuda* or argentin* or armenia* or aruba* or azerbaijan* or bahrain* or bangladesh* or bengal* or bangal* or barbados* or barbadian* or bajan or bajans or belarus* or belorus* or byelarus* or byelorus* or belize* or benin* or dahomey or bhutan* or bolivia* or bosnia* or herzegovin* or botswan* or batswan* or bechuanaland* or brazil* or brasil* or bulgaria* or burkina* or burkinese* or upper volta* or burundi* or urundi* or cabo verde* or cape verde* or cambodia* or kampuchea* or khmer* or cameroon* or cameroun* or ubangi shari* or chad* or chile* or china* or chinese or colombia* or comoro* or comore* or comorian* or mayotte* or congo* or zaire* or costa rica* or "cote d'ivoir*" or "cote d' ivoir*" or cote divoir* or cote d ivoir* or ivory coast* or ivorian* or croatia* or cuba or cuban or cubans or "cuba's" or cyprus* or cypriot* or czech* or djibouti* or french somaliland* or dominica* or ecuador* or egypt* or united arab republic* or el salvador* or salvadoran* or guinea* or equatoguinea* or eritrea* or estonia* or eswatini* or swaziland* or swazi* or swati* or ethiopia* or fiji* or gabon* or gabonese* or gabonaise* or gambia* or ((georgia or georgian or georgians) not (atlanta or california or florida)) or ghana* or gibraltar* or greece* or greek* or grecian* or grenada* or grenadian* or guam* or guatemala* or guyana* or guiana* or guyanese* or haiti* or hispaniola* or hondura* or hungary* or hungarian* or india* or indonesia* or iran* or iraq* or isle of man* or jamaica* or jordan* or kazakh* or kenya* or karabati* or korea* or kosovo* or kosova* or kyrgyz* or kirgiz* or kirghiz* or laos or lao or laotian* or latvia* or lebanon* or lebanese* or lesotho* or lesothan* or lesothonian* or basutoland* or mosotho* or basotho* or liberia* or libya* or jamahiriya* or lithuania* or macedonia* or madagasca* or malagasy* or malawi* or nyasaland* or malaysia* or malay* federation or maldives* or maldivian* or indian ocean or mali or malian* or "mali's" or malta or maltese* or "malta's" or micronesia* or marshallese* or kiribati* or marshall island* or nauru or nauran or nauruans or "naurian's" or mariana or marianas or palau or paluan* or tuvalu* or mauritania* or mauritan* or mauritius* or mexico* or mexican* or moldova* or moldovia* or mongol* or montenegr* or morocco* or moroccan* or ifni or mozambique* or mozambican* or myanmar* or burma* or burmese or namibia* or nepal* or new caledonia* or netherlands antill* or nicaragua* or niger* or oman or omani or omanis or "oman's" or pakistan* or palestin* or gaza* or west bank* or panama* or paraguay* or peru or peruvian* or "peru's" or philippine* or philipine* or phillipine* or phillippine* or filipino* or filipina* or poland* or polish or pole or poles or portugal* or portuguese or puerto ric* or romania* or russia* or ussr* or soviet* or rwanda* or rwandese or ruanda* or ruandese or samoa* or navigator island* or pacific island* or polynesia* or "sao tome and principe*" or sao tomean* or santomean* or saudi arabia* or saudi or saudis or senegal* or serbia* or seychell* or sierra leone* or slovak* or sloven* or melanesia* or solomon island* or norfolk island* or somali* or sri lanka* or ceylon* or "saint kitts and nevis*" or "st kitts and nevis*" or kittian* or nevisian* or saint lucia* or st lucia* or saint vincent* or st vincent* or vincentian* or grenadine* or sudan* or surinam* or syria* or tajik* or tadjik* or tadzhik* or tanzania* or tanganyika* or thai* or timor leste* or east timor* or timorese* or togo or togoles* or "togo's" or tonga* or trinidad* or tobago* or tunisia* or turkiy* or turkey* or turk or turks or turkish or turkmen* or uganda* or ukrain* or uruguay* or uzbek* or vanuatu* or new hebrides* or venezuela* or vietnam* or viet nam* or yemen* or yugoslav* or zambia* or zimbabwe* or rhodesia* or arab* countr* or middle east* or global south or sahara* or subsahara* or magreb* or maghrib* or west indies* or caribbean* or central america* or latin america* or south america* or central asia* or north asia* or northern asia* or southeastern asia* or south eastern asia* or southeast asia* or south east asia* or west asia* or western asia* or east europe* or eastern europe* or developing countr* or developing nation* or developing population* or developing world or less developed countr* or less developed nation* or less developed world or lesser developed countr* or lesser developed nation* or lesser developed world or under developed countr* or under developed nation* or under developed world or underdeveloped countr* or underdeveloped nation* or underdeveloped world or middle income countr* or middle income nation* or middle income population* or low income countr* or low income nation* or low income population* or lower income countr* or lower income nation* or lower income population* or underserved countr* or underserved nation* or underserved population* or under served population* or under served nation* or under served population* or deprived countr* or deprived population* or high burden countr* or high burden nation* or countdown countr* or countdown nation* or poor countr* or poor nation* or poor population* or poor world or poorer countr* or poorer nation* or poorer population* or poorer world or developing econom* or less developed econom* or underdeveloped econom* or under developed econom* or middle income econom* or low income econom* or lower income econom* or low gdp or low gnp or low gross domestic or low gross national or lower gdp or lower gnp or lower gross domestic or lower gross national or lmic or lmics or third world or lami countr* or transitional countr* or emerging econom* or emerging nation*).ti,ab,hw,kf. |
| 16 | 14 and 15 |
| 17 | Self Care/ or Self Sampling.mp. |
| 18 | self application.mp. |
| 19 | self administration.tw. or Self Administration/ |
| 20 | (self collection or self collected).mp. |
| 21 | Provider sampling.mp. |
| 22 | Specimen Handling/ or sample* collection.mp. |
| 23 | home-based.mp. |
| 24 | preferences.mp. or Patient Preference/ or Patient Acceptance of Health Care/ |
| 25 | *health personnel/ or *allied health personnel/ or *medical laboratory personnel/ or *medical staff/ or *nurses/ |
| 26 | (Costs.mp. and Cost Analysis/) or Health Services Accessibility/ or equity.ti,ab. or Socioeconomic Factors/ or cost effectiveness.ti,ab. |
| 27 | 17 or 18 or 19 or 20 or 21 or 22 or 23 or 24 or 25 or 26 |
| 28 | 16 and 27 |

**#2 Embase 1947-Present, updated daily**

| 1 | human papillomavirus.mp. or Wart virus/ |
| --- | --- |
| 2 | HPV.ti,ab. |
| 3 | uterine cervix dysplasia/ |
| 4 | uterine cervix tumor/ |
| 5 | ((cervix or cervical or cervic*) and (cancer or carcinoma or neoplas* or dysplas* or squamous)).ti,ab. |
| 6 | Papillomavirus Infection/ or Papillomaviridae/ |
| 7 | 1 or 2 or 3 or 4 or 5 or 6 |
| 8 | diagnostic test*.mp. |
| 9 | HPV test*.mp. |
| 10 | HPV screening.mp. |
| 11 | HC2.mp. |
| 12 | hybrid capture.ti,ab. |
| 13 | 8 or 9 or 10 or 11 or 12 |
| 14 | 7 and 13 |
| 15 | (afghan* or africa* or albania* or algeria* or angola* or antigua* or barbuda* or argentin* or armenia* or aruba* or azerbaijan* or bahrain* or bangladesh* or bengal* or bangal* or barbados* or barbadian* or bajan or bajans or belarus* or belorus* or byelarus* or byelorus* or belize* or benin* or dahomey or bhutan* or bolivia* or bosnia* or herzegovin* or botswan* or batswan* or bechuanaland* or brazil* or brasil* or bulgaria* or burkina* or burkinese* or upper volta* or burundi* or urundi* or cabo verde* or cape verde* or cambodia* or kampuchea* or khmer* or cameroon* or cameroun* or ubangi shari* or chad* or chile* or china* or chinese or colombia* or comoro* or comore* or comorian* or mayotte* or congo* or zaire* or costa rica* or "cote d'ivoir*" or "cote d' ivoir*" or cote divoir* or cote d ivoir* or ivory coast* or ivorian* or croatia* or cuba or cuban or cubans or "cuba's" or cyprus* or cypriot* or czech* or djibouti* or french somaliland* or dominica* or ecuador* or egypt* or united arab republic* or el salvador* or salvadoran* or guinea* or equatoguinea* or eritrea* or estonia* or eswatini* or swaziland* or swazi* or swati* or ethiopia* or fiji* or gabon* or gabonese* or gabonaise* or gambia* or ((georgia or georgian or georgians) not (atlanta or california or florida)) or ghana* or gibraltar* or greece* or greek* or grecian* or grenada* or grenadian* or guam* or guatemala* or guyana* or guiana* or guyanese* or haiti* or hispaniola* or hondura* or hungary* or hungarian* or india* or indonesia* or iran* or iraq* or isle of man* or jamaica* or jordan* or kazakh* or kenya* or karabati* or korea* or kosovo* or kosova* or kyrgyz* or kirgiz* or kirghiz* or laos or lao or laotian* or latvia* or lebanon* or lebanese* or lesotho* or lesothan* or lesothonian* or basutoland* or mosotho* or basotho* or liberia* or libya* or jamahiriya* or lithuania* or macedonia* or madagasca* or malagasy* or malawi* or nyasaland* or malaysia* or malay* federation or maldives* or maldivian* or indian ocean or mali or malian* or "mali's" or malta or maltese* or "malta's" or micronesia* or marshallese* or kiribati* or marshall island* or nauru or nauran or nauruans or "naurian's" or mariana or marianas or palau or paluan* or tuvalu* or mauritania* or mauritan* or mauritius* or mexico* or mexican* or moldova* or moldovia* or mongol* or montenegr* or morocco* or moroccan* or ifni or mozambique* or mozambican* or myanmar* or burma* or burmese or namibia* or nepal* or new caledonia* or netherlands antill* or nicaragua* or niger* or oman or omani or omanis or "oman's" or pakistan* or palestin* or gaza* or west bank* or panama* or paraguay* or peru or peruvian* or "peru's" or philippine* or philipine* or phillipine* or phillippine* or filipino* or filipina* or poland* or polish or pole or poles or portugal* or portuguese or puerto ric* or romania* or russia* or ussr* or soviet* or rwanda* or rwandese or ruanda* or ruandese or samoa* or navigator island* or pacific island* or polynesia* or "sao tome and principe*" or sao tomean* or santomean* or saudi arabia* or saudi or saudis or senegal* or serbia* or seychell* or sierra leone* or slovak* or sloven* or melanesia* or solomon island* or norfolk island* or somali* or sri lanka* or ceylon* or "saint kitts and nevis*" or "st kitts and nevis*" or kittian* or nevisian* or saint lucia* or st lucia* or saint vincent* or st vincent* or vincentian* or grenadine* or sudan* or surinam* or syria* or tajik* or tadjik* or tadzhik* or tanzania* or tanganyika* or thai* or timor leste* or east timor* or timorese* or togo or togoles* or "togo's" or tonga* or trinidad* or tobago* or tunisia* or turkiy* or turkey* or turk or turks or turkish or turkmen* or uganda* or ukrain* or uruguay* or uzbek* or vanuatu* or new hebrides* or venezuela* or vietnam* or viet nam* or yemen* or yugoslav* or zambia* or zimbabwe* or rhodesia* or arab* countr* or middle east* or global south or sahara* or subsahara* or magreb* or maghrib* or west indies* or caribbean* or central america* or latin america* or south america* or central asia* or north asia* or northern asia* or southeastern asia* or south eastern asia* or southeast asia* or south east asia* or west asia* or western asia* or east europe* or eastern europe* or developing countr* or developing nation* or developing population* or developing world or less developed countr* or less developed nation* or less developed world or lesser developed countr* or lesser developed nation* or lesser developed world or under developed countr* or under developed nation* or under developed world or underdeveloped countr* or underdeveloped nation* or underdeveloped world or middle income countr* or middle income nation* or middle income population* or low income countr* or low income nation* or low income population* or lower income countr* or lower income nation* or lower income population* or underserved countr* or underserved nation* or underserved population* or under served population* or under served nation* or under served population* or deprived countr* or deprived population* or high burden countr* or high burden nation* or countdown countr* or countdown nation* or poor countr* or poor nation* or poor population* or poor world or poorer countr* or poorer nation* or poorer population* or poorer world or developing econom* or less developed econom* or underdeveloped econom* or under developed econom* or middle income econom* or low income econom* or lower income econom* or low gdp or low gnp or low gross domestic or low gross national or lower gdp or lower gnp or lower gross domestic or lower gross national or lmic or lmics or third world or lami countr* or transitional countr* or emerging econom* or emerging nation*).ti,ab,hw,kf. |
| 16 | 14 and 15 |
| 17 | Self Care/ or Self Sampling.mp. |
| 18 | self application.mp. |
| 19 | self administration.tw. or Self Administration/ |
| 20 | (self collection or self collected).mp. |
| 21 | Provider sampling.mp. |
| 22 | Specimen Handling/ or sample* collection.mp. |
| 23 | home-based.mp. |
| 24 | preferences.tw. or Patient Preference/ or Patient Attitude/ |
| 25 | health care personnel/ |
| 26 | "cost benefit analysis"/ or Health care access/ or health equity/ or Socioeconomic parameters/ or cost effectiveness.ti,ab. |
| 27 | 17 or 18 or 19 or 20 or 21 or 22 or 23 or 24 or 25 or 26 |
| 28 | 16 and 27 |

**Interface - EBSCOhost Research Databases**

**#3 Database - CINAHL**

| # | Query |
| --- | --- |
| S1 | TX ( human papillomavirus or human papilloma virus or hpv ) OR MH uterine cervical neoplasms OR MH uterine cervical dysplasia OR TX ( ((cervix or cervical or cervic*) and (cancer or carcinoma or neoplas* or dysplas* or squamous)) ) |
| S2 | TX diagnostic test OR TX ( HPV and (test* or screen*) ) OR TX ( HC2 or "hybrid capture" ) |
| S3 | S1 AND S2 |
| S4 | TX (afghan* or africa* or albania* or algeria* or angola* or antigua* or barbuda* or argentin* or armenia* or aruba* or azerbaijan* or bahrain* or bangladesh* or bengal* or bangal* or barbados* or barbadian* or bajan or bajans or belarus* or belorus* or byelarus* or byelorus* or belize* or benin* or dahomey or bhutan* or bolivia* or bosnia* or herzegovin* or botswan* or batswan* or bechuanaland* or brazil* or brasil* or bulgaria* or burkina* or burkinese* or upper volta* or burundi* or urundi* or cabo verde* or cape verde* or cambodia* or kampuchea* or khmer* or cameroon* or cameroun* or ubangi shari* or chad* or chile* or china* or chinese or colombia* or comoro* or comore* or comorian* or mayotte* or congo* or zaire* or costa rica* or "cote d'ivoir*" or "cote d' ivoir*" or cote divoir* or cote d ivoir* or ivory coast* or ivorian* or croatia* or cuba or cuban or cubans or "cuba's" or cyprus* or cypriot* or czech* or djibouti* or french somaliland* or dominica* or ecuador* or egypt* or united arab republic* or el salvador* or salvadoran* or guinea* or equatoguinea* or eritrea* or estonia* or eswatini* or swaziland* or swazi* or swati* or ethiopia* or fiji* or gabon* or gabonese* or gabonaise* or gambia* or ((georgia or georgian or georgians) not (atlanta or california or florida)) or ghana* or gibraltar* or greece* or greek* or grecian* or grenada* or grenadian* or guam* or guatemala* or guyana* or guiana* or guyanese* or haiti* or hispaniola* or hondura* or hungary* or hungarian* or india* or indonesia* or iran* or iraq* or isle of man* or jamaica* or jordan* or kazakh* or kenya* or karabati* or korea* or kosovo* or kosova* or kyrgyz* or kirgiz* or kirghiz* or laos or lao or laotian* or latvia* or lebanon* or lebanese* or lesotho* or lesothan* or lesothonian* or basutoland* or mosotho* or basotho* or liberia* or libya* or jamahiriya* or lithuania* or macedonia* or madagasca* or malagasy* or malawi* or nyasaland* or malaysia* or malay* federation or maldives* or maldivian* or indian ocean or mali or malian* or "mali's" or malta or maltese* or "malta's" or micronesia* or marshallese* or kiribati* or marshall island* or nauru or nauran or nauruans or "naurian's" or mariana or marianas or palau or paluan* or tuvalu* or mauritania* or mauritan* or mauritius* or mexico* or mexican* or moldova* or moldovia* or mongol* or montenegr* or morocco* or moroccan* or ifni or mozambique* or mozambican* or myanmar* or burma* or burmese or namibia* or nepal* or new caledonia* or netherlands antill* or nicaragua* or niger* or oman or omani or omanis or "oman's" or pakistan* or palestin* or gaza* or west bank* or panama* or paraguay* or peru or peruvian* or "peru's" or philippine* or philipine* or phillipine* or phillippine* or filipino* or filipina* or poland* or polish or pole or poles or portugal* or portuguese or puerto ric* or romania* or russia* or ussr* or soviet* or rwanda* or rwandese or ruanda* or ruandese or samoa* or navigator island* or pacific island* or polynesia* or "sao tome and principe*" or sao tomean* or santomean* or saudi arabia* or saudi or saudis or senegal* or serbia* or seychell* or sierra leone* or slovak* or sloven* or melanesia* or solomon island* or norfolk island* or somali* or sri lanka* or ceylon* or "saint kitts and nevis*" or "st kitts and nevis*" or kittian* or nevisian* or saint lucia* or st lucia* or saint vincent* or st vincent* or vincentian* or grenadine* or sudan* or surinam* or syria* or tajik* or tadjik* or tadzhik* or tanzania* or tanganyika* or thai* or timor leste* or east timor* or timorese* or togo or togoles* or "togo's" or tonga* or trinidad* or tobago* or tunisia* or turkiy* or turkey* or turk or turks or turkish or turkmen* or uganda* or ukrain* or uruguay* or uzbek* or vanuatu* or new hebrides* or venezuela* or vietnam* or viet nam* or yemen* or yugoslav* or zambia* or zimbabwe* or rhodesia* or arab* countr* or middle east* or global south or sahara* or subsahara* or magreb* or maghrib* or west indies* or caribbean* or central america* or latin america* or south america* or central asia* or north asia* or northern asia* or southeastern asia* or south eastern asia* or southeast asia* or south east asia* or west asia* or western asia* or east europe* or eastern europe* or developing countr* or developing nation* or developing population* or developing world or less developed countr* or less developed nation* or less developed world or lesser developed countr* or lesser developed nation* or lesser developed world or under developed countr* or under developed nation* or under developed world or underdeveloped countr* or underdeveloped nation* or underdeveloped world or middle income countr* or middle income nation* or middle income population* or low income countr* or low income nation* or low income population* or lower income countr* or lower income nation* or lower income population* or underserved countr* or underserved nation* or underserved population* or under served population* or under served nation* or under served population* or deprived countr* or deprived population* or high burden countr* or high burden nation* or countdown countr* or countdown nation* or poor countr* or poor nation* or poor population* or poor world or poorer countr* or poorer nation* or poorer population* or poorer world or developing econom* or less developed econom* or underdeveloped econom* or under developed econom* or middle income econom* or low income econom* or lower income econom* or low gdp or low gnp or low gross domestic or low gross national or lower gdp or lower gnp or lower gross domestic or lower gross national or lmic or lmics or third world or lami countr* or transitional countr* or emerging econom* or emerging nation*) |
| S5 | S3 AND S4 |
| S6 | TX self and (sampl* or care or application or administration or collect*) |
| S7 | TX provider sampling OR TX ( sample collection or specimen collection ) OR TX home-based |
| S8 | MH patient preference OR MH Patient Acceptance of Health Care OR MH health personnel |
| S9 | TX ( costs or cost or expense ) OR TX cost benefit analysis OR TX equity OR MH socioeconomic factors |
| S10 | S6 OR S7 OR S8 OR S9 |
| S11 | S5 AND S10 |

**#4 Search Name: Cochrane Central Register of Controlled Trials**

**Issue 7 of 12, July 2023**

| ID | Search |
| --- | --- |
| #1 | human papillomavirus |
| #2 | MeSH descriptor: [Human Papillomavirus Viruses] explode all trees |
| #3 | (HPV):ti,ab,kw |
| #4 | MeSH descriptor: [Uterine Cervical Dysplasia] explode all trees |
| #5 | MeSH descriptor: [Uterine Cervical Neoplasms] explode all trees |
| #6 | (((cervix or cervical or cervic*) and (cancer or carcinoma or neoplas* or dysplas* or squamous))):ti,ab,kw |
| #7 | #1 or #2 or #3 or #4 or #5 or #6 |
| #8 | (diagnostic test):ti,ab,kw |
| #9 | MeSH descriptor: [Diagnostic Tests, Routine] explode all trees |
| #10 | (HPV and (test* or screen*)):ti,ab,kw |
| #11 | (HC2 or hybrid capture):ti,ab,kw |
| #12 | #8 or #9 or #10 or #11 |
| #13 | #7 and #12 |
| #14 | ((afghan* OR africa* OR albania* OR algeria* OR angola* OR antigua* OR barbuda* OR argentin* OR armenia* OR aruba* OR azerbaijan* OR bahrain* OR bangladesh* OR bengal* OR bangal* OR barbados* OR barbadian* OR bajan OR bajans OR belarus* OR belorus* OR byelarus* OR byelorus* OR belize* OR benin* OR dahomey OR bhutan* OR bolivia* OR bosnia* OR herzegovin* OR botswan* OR batswan* OR bechuanaland OR brazil* OR brasil* OR bulgaria* OR burkina* OR burkinese* OR upper-volta* OR burundi* OR urundi* OR cabo-verde* OR cape-verde* OR cambodia* OR kampuchea* OR khmer* OR cameroon* OR cameroun* OR ubangi-shari* OR chad* OR chile* OR china* OR chinese OR colombia* OR comoro* OR comore* OR comorian* OR mayotte* OR congo* OR zaire* OR costa-rica* OR (cote* AND *ivoir*) OR ivory-coast* OR ivorian* OR croatia* OR cuba* OR cyprus* OR cypriot* OR czech* OR djibouti* OR french-somaliland* OR dominica* OR ecuador* OR egypt* OR united-arab-republic* OR el-salvador* OR salvadoran* OR guinea* OR equatoguinea* OR eritrea* OR estonia* OR eswatini* OR swaziland* OR swazi* OR swati* OR ethiopia* OR fiji* OR gabon* OR gabonese* OR gabonaise* OR gambia* OR ((georgia OR georgian OR georgians) NOT (atlanta OR california OR florida)) OR ghana* OR gibraltar* OR greece* OR greek* OR grecian* OR grenada* OR grenadian* OR guam* OR guatemala* OR guyana* OR guiana* OR guyanese* OR haiti* OR hispaniola* OR hondura* OR hungary* OR hungarian* OR india* OR indonesia* OR iran* OR iraq* OR isle-of-man* OR jamaica* OR jordan* OR kazakh* OR kenya* OR karabati* OR korea* OR kosovo* OR kosova* OR kyrgyz* OR kirgiz* OR kirghiz* OR laos OR lao OR laotian* OR latvia* OR lebanon* OR lebanese* OR lesotho* OR lesothan* OR lesothonian* OR basutoland* OR mosotho* OR basotho* OR liberia* OR libya* OR jamahiriya* OR lithuania* OR macedonia* OR madagasca* OR malagasy* OR malawi* OR nyasaland* OR malaysia* OR malay-federation OR malaya-federation OR malayan-federation OR maldives* OR maldivian* OR indian-ocean* OR mali* OR malta* OR maltese* OR micronesia* OR marshallese* OR kiribati* OR marshall-island* OR nauru OR nauran OR nauruans OR nauran* OR mariana OR marianas OR palau OR paluan* OR tuvalu* OR mauritania* OR mauritan* OR mauritius* OR mexico* OR mexican* OR moldova* OR moldovia* OR mongol* OR montenegr* OR morocco* OR moroccan* OR ifni OR mozambique* OR mozambican* OR myanmar* OR burma* OR burmese OR namibia* OR nepal* OR new-caledonia* OR netherlands-antill* OR nicaragua* OR niger* OR oman* OR pakistan* OR palestin* OR gaza* OR west-bank* OR panama* OR paraguay* OR peru* OR philippine* OR philipine* OR phillipine* OR phillippine* OR filipino* OR filipina* OR poland* OR polish OR pole OR poles OR portugal* OR portuguese OR puerto-ric* OR romania* OR russia* OR ussr* OR soviet* OR rwanda* OR rwandese OR ruanda* OR ruandese OR samoa* OR navigator-island* OR pacific-island* OR polynesia* OR sao-tome* OR santomean* OR saudi-arabia* OR saudi OR saudis OR senegal* OR serbia* OR seychell* OR sierra-leone* OR slovak* OR sloven* OR melanesia* OR solomon-island* OR norfolk-island* OR somali* OR sri-lanka* OR ceylon* OR saint-kitts* OR st-kitts* OR kittian* OR nevisian* OR saint-lucia* OR st-lucia* OR saint-vincent* OR st-vincent* OR vincentian* OR grenadine* OR sudan* OR surinam* OR syria* OR tajik* OR tadjik* OR tadzhik* OR tanzania* OR tanganyika* OR thai* OR timor-leste* OR east-timor* OR timorese* OR togo* OR tonga* OR trinidad* OR tobago* OR tunisia* OR turkiy* OR turkey* OR turk OR turks OR turkish OR turkmen* OR uganda* OR ukrain* OR uruguay* OR uzbek* OR vanuatu* OR new-hebrides OR venezuela* OR vietnam* OR viet-nam* OR yemen* OR yugoslav* OR zambia* OR zimbabwe* OR rhodesia* OR arab-countr* OR arabic-countr* OR middle-east* OR global-south OR sahara* OR subsahara* OR magreb* OR maghrib* OR west-indies* OR caribbean* OR central-america* OR latin-america* OR south-america* OR central-asia* OR north-asia* OR northern-asia* OR southeastern-asia* OR south-eastern-asia* OR southeast-asia* OR south-east-asia* OR west-asia* OR western-asia* OR east-europe* OR eastern-europe* OR developing-countr* OR developing-nation* OR developing-population* OR developing-world OR less-developed-countr* OR less-developed-nation* OR less-developed-world OR lesser-developed-countr* OR lesser-developed-nation* OR lesser-developed-world OR under-developed-countr* OR under-developed-nation* OR under-developed-world OR underdeveloped-countr* OR underdeveloped-nation* OR underdeveloped-world OR middle-income-countr* OR middle-income-nation* OR middle-income-population* OR low-income-countr* OR low-income-nation* OR low-income-population* OR lower-income-countr* OR lower-income-nation* OR lower-income-population* OR underserved-countr* OR underserved-nation* OR underserved-population* OR under-served-population* OR under-served-nation* OR under-served-population* OR deprived-countr* OR deprived-population* OR high-burden-countr* OR high-burden-nation* OR countdown-countr* OR countdown-nation* OR poor-countr* OR poor-nation* OR poor-population* OR poor-world OR poorer-countr* OR poorer-nation* OR poorer-population* OR poorer-world OR developing-econom* OR less developed-econom* OR underdeveloped-econom* OR under-developed-econom* OR middle-income-econom* OR low-income-econom* OR lower-income-econom* OR low-gdp OR low-gnp OR low-gross-domestic OR low-gross-national OR lower-gdp OR lower-gnp OR lower-gross-domestic OR lower-gross-national OR lmic OR lmics OR third-world OR lami-countr* OR transitional-countr* OR emerging-econom* OR emerging-nation*)):ti,ab,kw |
| #15 | #13 and #14 |
| #16 | (self sampling):ti,ab,kw |
| #17 | MeSH descriptor: [Self Care] explode all trees |
| #18 | (self application or self administration or self collection):ti,ab,kw |
| #19 | (Provider sampling):ti,ab,kw |
| #20 | MeSH descriptor: [Specimen Handling] explode all trees |
| #21 | (home-based):ti,ab,kw |
| #22 | (preference*):ti,ab,kw |
| #23 | MeSH descriptor: [Patient Acceptance of Health Care] explode all trees |
| #24 | (health personnel):ti,ab,kw |
| #25 | ("cost effectiveness"):ti,ab,kw |
| #26 | MeSH descriptor: [Health Services Accessibility] explode all trees |
| #27 | (equity):ti,ab,kw |
| #28 | MeSH descriptor: [Socioeconomic Factors] explode all trees |
| #29 | #16 or #17 or #18 or #19 or #20 or #21 or #22 or #23 or #24 or #25 or #26 or #27 or #28 |

**#5 Web of Science – Science Citation Index Expanded**

| Search Query |
| --- |
| #1 human papillomavirus or HPV (Topic) OR ((cervix or cervical or cervic*) and (cancer or carcinoma or neoplas* or dysplas* or squamous)). (Topic) |
| #2 diagnostic test* or screening or HC2 or "hybrid capture" (Topic) |
| #3 #1 AND #2 |
| #4 (afghan* or africa* or albania* or algeria* or angola* or antigua* or barbuda* or argentin* or armenia* or aruba* or azerbaijan* or bahrain* or bangladesh* or bengal* or bangal* or barbados* or barbadian* or bajan or bajans or belarus* or belorus* or byelarus* or byelorus* or belize* or benin* or dahomey or bhutan* or bolivia* or bosnia* or herzegovin* or botswan* or batswan* or bechuanaland* or brazil* or brasil* or bulgaria* or burkina* or burkinese* or upper volta* or burundi* or urundi* or cabo verde* or cape verde* or cambodia* or kampuchea* or khmer* or cameroon* or cameroun* or ubangi shari* or chad* or chile* or china* or chinese or colombia* or comoro* or comore* or comorian* or mayotte* or congo* or zaire* or costa rica* or "cote d'ivoir*" or "cote d' ivoir*" or cote divoir* or cote d ivoir* or ivory coast* or ivorian* or croatia* or cuba or cuban or cubans or "cuba's" or cyprus* or cypriot* or czech* or djibouti* or french somaliland* or dominica* or ecuador* or egypt* or united arab republic* or el salvador* or salvadoran* or guinea* or equatoguinea* or eritrea* or estonia* or eswatini* or swaziland* or swazi* or swati* or ethiopia* or fiji* or gabon* or gabonese* or gabonaise* or gambia* or ((georgia or georgian or georgians) not (atlanta or california or florida)) or ghana* or gibraltar* or greece* or greek* or grecian* or grenada* or grenadian* or guam* or guatemala* or guyana* or guiana* or guyanese* or haiti* or hispaniola* or hondura* or hungary* or hungarian* or india* or indonesia* or iran* or iraq* or isle of man* or jamaica* or jordan* or kazakh* or kenya* or karabati* or korea* or kosovo* or kosova* or kyrgyz* or kirgiz* or kirghiz* or laos or lao or laotian* or latvia* or lebanon* or lebanese* or lesotho* or lesothan* or lesothonian* or basutoland* or mosotho* or basotho* or liberia* or libya* or jamahiriya* or lithuania* or macedonia* or madagasca* or malagasy* or malawi* or nyasaland* or malaysia* or malay* federation or maldives* or maldivian* or indian ocean or mali or malian* or "mali's" or malta or maltese* or "malta's" or micronesia* or marshallese* or kiribati* or marshall island* or nauru or nauran or nauruans or "naurian's" or mariana or marianas or palau or paluan* or tuvalu* or mauritania* or mauritan* or mauritius* or mexico* or mexican* or moldova* or moldovia* or mongol* or montenegr* or morocco* or moroccan* or ifni or mozambique* or mozambican* or myanmar* or burma* or burmese or namibia* or nepal* or new caledonia* or netherlands antill* or nicaragua* or niger* or oman or omani or omanis or "oman's" or pakistan* or palestin* or gaza* or west bank* or panama* or paraguay* or peru or peruvian* or "peru's" or philippine* or philipine* or phillipine* or phillippine* or filipino* or filipina* or poland* or polish or pole or poles or portugal* or portuguese or puerto ric* or romania* or russia* or ussr* or soviet* or rwanda* or rwandese or ruanda* or ruandese or samoa* or navigator island* or pacific island* or polynesia* or "sao tome and principe*" or sao tomean* or santomean* or saudi arabia* or saudi or saudis or senegal* or serbia* or seychell* or sierra leone* or slovak* or sloven* or melanesia* or solomon island* or norfolk island* or somali* or sri lanka* or ceylon* or "saint kitts and nevis*" or "st kitts and nevis*" or kittian* or nevisian* or saint lucia* or st lucia* or saint vincent* or st vincent* or vincentian* or grenadine* or sudan* or surinam* or syria* or tajik* or tadjik* or tadzhik* or tanzania* or tanganyika* or thai* or timor leste* or east timor* or timorese* or togo or togoles* or "togo's" or tonga* or trinidad* or tobago* or tunisia* or turkiy* or turkey* or turk or turks or turkish or turkmen* or uganda* or ukrain* or uruguay* or uzbek* or vanuatu* or new hebrides* or venezuela* or vietnam* or viet nam* or yemen* or yugoslav* or zambia* or zimbabwe* or rhodesia* or arab* countr* or middle east* or global south or sahara* or subsahara* or magreb* or maghrib* or west indies* or caribbean* or central america* or latin america* or south america* or central asia* or north asia* or northern asia* or southeastern asia* or south eastern asia* or southeast asia* or south east asia* or west asia* or western asia* or east europe* or eastern europe* or developing countr* or developing nation* or developing population* or developing world or less developed countr* or less developed nation* or less developed world or lesser developed countr* or lesser developed nation* or lesser developed world or under developed countr* or under developed nation* or under developed world or underdeveloped countr* or underdeveloped nation* or underdeveloped world or middle income countr* or middle income nation* or middle income population* or low income countr* or low income nation* or low income population* or lower income countr* or lower income nation* or lower income population* or underserved countr* or underserved nation* or underserved population* or under served population* or under served nation* or under served population* or deprived countr* or deprived population* or high burden countr* or high burden nation* or countdown countr* or countdown nation* or poor countr* or poor nation* or poor population* or poor world or poorer countr* or poorer nation* or poorer population* or poorer world or developing econom* or less developed econom* or underdeveloped econom* or under developed econom* or middle income econom* or low income econom* or lower income econom* or low gdp or low gnp or low gross domestic or low gross national or lower gdp or lower gnp or lower gross domestic or lower gross national or lmic or lmics or third world or lami countr* or transitional countr* or emerging econom* or emerging nation*). (Topic) |
| #5 #3 AND #4 |
| #6 self application or Self Administration or self collection (Topic) OR Provider sampling or home-based (Topic) |
| #7 preferences or health personnel or equity or cost-effectiveness (Topic) |
| #8 #6 OR #7 |
| #9 #5 AND #8 |

**# 6 Global index Medicus**

tw:( (tw:(papillomavirus OR hpv OR cervical cancer OR cervical dysplasia)) AND (tw:(diagnosis OR screening OR hc2 OR hybrid capture))) AND ( mj:("Uterine Cervical Dysplasia" OR "Humans" OR "Uterine Cervical Neoplasms" OR "Papillomavirus Infections" OR "Female" OR "Papillomaviridae" OR "Adult" OR "Middle Aged" OR "Cervix Uteri")

**# 7 MedRxiv**

"HPV diagnostic test home-based"

**# 8 Scopus**

**Search Article Title, Abstract, Keywords:**

papillomavirus OR hpv OR "cervical cancer" OR "cervical dysplasia

AND

self-testing OR home-based OR self-collection OR cost-effectiveness OR economic* OR equity OR preference* OR socioeconomic

AND

lmic OR "low and middle income countries" OR africa OR asia OR latin AND america OR developing AND countries

**# 9 Clinicaltrials.gov**

screening | Recruiting, not yet recruiting, Active, not recruiting, Enrolling by invitation Studies | HPV | Studies with Female Participants

Also searched for Human papilloma virus, Papillomavirus Infection.

**# 10 WHO ICTRP**

HPV and self-sampling

**#11 WHO website**

HPV and self-sampling

**# 12 International Agency for Research on Cancer (IARC)**

HPV and self-sampling

**# 13 International AIDS Society (IAS)**

HPV and self-sampling

**#14 International Conference of AIDS and STIs in Africa (ICASA)**

HPV and self-sampling

#**15 Citation searching**

Manual search through the reference list for studies done on HPV and self-sampling
