# Supplementary material for: Human papillomavirus self-sampling versus provider-sampling in low- and middle-income countries: a scoping review of accuracy, acceptability, cost, uptake, and equity
Source: Front Public Health. 2024 Nov 29;12:1439164. doi: 10.3389/fpubh.2024.1439164 (PMC11638174; doi:10.3389/fpubh.2024.1439164)
Supplement: Supplementary file 4 [file Table_4.docx]

# Annex 4: PROGRESS PLUS Equity Factors Explanation

| **Equity Factor** | | **Definition** |
| --- | --- | --- |
| **P**lace of residence | | The geographical areas where participants live may impact access to healthcare services, e.g., rural, peri-urban or urban. |
| **R**ace, ethnicity, culture or language | | Racial, social, language and cultural determinants may contribute to health disparities, e.g., participants of different ethnic groups. |
| **O**ccupation | | The type of work in which the participants engage may impact their level of exposure to occupational health hazards, e.g., female sex workers. |
| **G**ender/ sexual identity | | Variations in sexual identity perceptions may challenge the process of tailoring interventions towards the population, e.g., transgender participants. |
| **R**eligion | | Religious beliefs and practices that can impact participants’ behavior towards the intervention, e.g., the role of various denominations such as Christianity/ Hinduism/Islam/ any other in health research. |
| **E**ducation | | Participants’ educational attainments directly relate to their access to information on health improvement, e.g., Formal/ non-formal/none, Primary/secondary/tertiary. |
| **S**ocioeconomic status | | The income received by the participants may impact their access and affordability of healthcare, e.g., monthly income per household. |
| **S**ocial capital | | Social relationships and support systems/groups may influence health outcomes, e.g., social networks/ affiliated with an individual. |
| **PLUS** | **A**ge | The age of the participants plays a role in ensuring all targeted age groups for the intervention have been assessed by considering the mean or median ages. |
|  | **D**isability | Factors that hinder individuals from accomplishing certain activities directly impact establishing an inclusive healthcare system, e.g., blindness. |
|  | **C**omorbidity | Multiple health conditions may influence participants’ access to health care, e.g., Human Immunodeficiency Virus or other sexually transmitted infections. |
