# Supplementary material for: Human papillomavirus self-sampling versus provider-sampling in low- and middle-income countries: a scoping review of accuracy, acceptability, cost, uptake, and equity
Source: Front Public Health. 2024 Nov 29;12:1439164. doi: 10.3389/fpubh.2024.1439164 (PMC11638174; doi:10.3389/fpubh.2024.1439164)
Supplement: Supplementary file 5 [file Table_5.docx]

# **Annex 5: Characteristics of excluded studies and guidelines**

| Study ID | Reason for exclusion |
| --- | --- |
| 1. Aarnio 2020 ([1](#_ENREF_1)) | Ineligible context |
| 1. ACTRN12613000906752 2013 ([2](#_ENREF_2)) | Inaccessible full text |
| 1. Wong 2016 ([3](#_ENREF_3)) | Ineligible context |
| 1. Adegboyega 2022 ([4](#_ENREF_4)) | Ineligible context |
| 1. Adler 2013 ([5](#_ENREF_5)) | Ineligible concept |
| 1. Adsul 2019 ([6](#_ENREF_6)) | Ineligible concept |
| 1. Ajenifuja 2018 ([7](#_ENREF_7)) | Ineligible concept |
| 1. Allende 2020 ([8](#_ENREF_8)) | Ineligible concept |
| 1. Arbyn 2022 ([9](#_ENREF_9)) | Ineligible concept |
| 1. Avian 2022 ([10](#_ENREF_10)) | Ineligible context |
| 1. Awua 2017 ([11](#_ENREF_11)) | Ineligible concept |
| 1. Aziz 2012 ([12](#_ENREF_12)) | Ineligible article type |
| 1. Bante 2019 ([13](#_ENREF_13)) | Ineligible concept |
| 1. Barbee 2010 ([14](#_ENREF_14)) | Ineligible context |
| 1. Baussano 2017 ([15](#_ENREF_15)) | Ineligible concept |
| 1. Beal 2014 ([16](#_ENREF_16)) | Ineligible concept |
| 1. Belinson 2017 ([17](#_ENREF_17)) | Ineligible article type |
| 1. Benski 2019 ([18](#_ENREF_18)) | Ineligible concept |
| 1. Berrada 2021 ([19](#_ENREF_19)) | Ineligible concept |
| 1. Bhatla 2015 ([20](#_ENREF_20)) | Ineligible article type |
| 1. Bidus 2005 ([21](#_ENREF_21)) | Ineligible article type |
| 1. Bigoni 2015 ([22](#_ENREF_22)) | Ineligible concept |
| 1. Bohn 2022 ([23](#_ENREF_23)) | Ineligible context |
| 1. Bottari 2017 ([24](#_ENREF_24)) | Ineligible context |
| 1. Breidenthal 2019 ([25](#_ENREF_25)) | Ineligible article type |
| 1. Brito 2022 ([26](#_ENREF_26)) | Not in English |
| 1. Broquet 2022 ([27](#_ENREF_27)) | Ineligible concept |
| 1. Camel 2017 ([28](#_ENREF_28)) | Ineligible article type |
| 1. Campos 2015 ([29](#_ENREF_29)) | Ineligible concept |
| 1. Campos 2017 ([30](#_ENREF_30)) | Ineligible concept |
| 1. Carozzi 2022 ([31](#_ENREF_31)) | Ineligible article type |
| 1. Castle 2020 ([32](#_ENREF_32)) | Ineligible concept |
| 1. Chang 2002 ([33](#_ENREF_33)) | Ineligible context |
| 1. Chatterjee 2021 ([34](#_ENREF_34)) | Ineligible article type |
| 1. Chatzistamatiou 2020 ([35](#_ENREF_35)) | Ineligible context |
| 1. Chen 2016 ([36](#_ENREF_36)) | Ineligible population |
| 1. ChiCTR2200056212 2022 ([37](#_ENREF_37)) | Ineligible article type |
| 1. Cho 2020 ([38](#_ENREF_38)) | Ineligible context |
| 1. Clifford 2023 ([39](#_ENREF_39)) | Ineligible concept |
| 1. CTRI/2020/11/029419 2020 ([40](#_ENREF_40)) | Ineligible article type |
| 1. Des Marais 2018 ([41](#_ENREF_41)) | Ineligible context |
| 1. Du 2017 ([42](#_ENREF_42)) | Ineligible article type |
| 1. Du 2020 ([43](#_ENREF_43)) | Ineligible concept |
| 1. Franciscatto 2014 ([44](#_ENREF_44)) | Ineligible concept |
| 1. Girianelli 2006 ([45](#_ENREF_45)) | Ineligible concept |
| 1. Goldie 2005 ([46](#_ENREF_46)) | Ineligible concept |
| 1. Gravitt 2014 ([47](#_ENREF_47)) | Ineligible article type |
| 1. Grigore 2022 ([48](#_ENREF_48)) | Ineligible context |
| 1. Guillaume 2020 ([49](#_ENREF_49)) | Ineligible concept |
| 1. Guo 2021 ([50](#_ENREF_50)) | Not in English |
| 1. Hailu 2013 ([51](#_ENREF_51)) | Ineligible population |
| 1. Hamzah 2013 ([52](#_ENREF_52)) | Ineligible article type |
| 1. Hariprasad 2023 ([53](#_ENREF_53)) | Ineligible concept |
| 1. Hernandez-Marquez 2014 ([54](#_ENREF_54)) | Not in English |
| 1. Hpv-Dna 2022 ([55](#_ENREF_55)) | Not in English |
| 1. Hussain 2014 ([56](#_ENREF_56)) | Ineligible article type |
| 1. Isrctn 2020 ([57](#_ENREF_57)) | Inaccessible full text |
| 1. ISRCTN43310942 2015 ([58](#_ENREF_58)) | Ineligible context |
| 1. Jede 2020 ([59](#_ENREF_59)) | Ineligible concept |
| 1. Jingran 2021 ([60](#_ENREF_60)) | Ineligible article type |
| 1. Johnson 2014 ([61](#_ENREF_61)) | Ineligible concept |
| 1. Jun, 2016 ([62](#_ENREF_62)) | Ineligible concept |
| 1. Kahesa 2023 ([63](#_ENREF_63)) | Ineligible concept |
| 1. Kang 2014 ([64](#_ENREF_64)) | Ineligible concept |
| 1. Keane 2021 ([65](#_ENREF_65)) | Ineligible concept |
| 1. Khanna 2007 ([66](#_ENREF_66)) | Ineligible context |
| 1. Khoo 2020 ([67](#_ENREF_67)) | Ineligible article type |
| 1. Kim 2021 ([68](#_ENREF_68)) | Ineligible context |
| 1. Klischke 2021 ([69](#_ENREF_69)) | Ineligible concept |
| 1. Kovalyova 2023 ([70](#_ENREF_70)) | Ineligible article type |
| 1. Krings 2016 ([71](#_ENREF_71)) | Ineligible article type |
| 1. Kuhn 2016 ([72](#_ENREF_72)) | Ineligible article type |
| 1. Kuhn 2018 ([73](#_ENREF_73)) | Ineligible article type |
| 1. Kuriakose 2020 ([74](#_ENREF_74)) | Ineligible population |
| 1. Latiff 2015 ([75](#_ENREF_75)) | Ineligible article type |
| 1. Lazcano-Ponce 2011 ([76](#_ENREF_76)) | Ineligible comparator |
| 1. Lazcano-Ponce 2014 ([77](#_ENREF_77)) | Ineligible concept |
| 1. Le 2022 ([78](#_ENREF_78)) | Ineligible context |
| 1. Lee 2021 ([79](#_ENREF_79)) | Ineligible concept |
| 1. Liling 2022 ([80](#_ENREF_80)) | Ineligible context |
| 1. Lim 2017 ([81](#_ENREF_81)) | Ineligible concept |
| 1. Lin 2017 ([82](#_ENREF_82)) | Ineligible concept |
| 1. Lince-Deroche 2015 ([83](#_ENREF_83)) | Ineligible concept |
| 1. Lorenzi 2013 ([84](#_ENREF_84)) | Ineligible concept |
| 1. Mandelblatt 2002 ([85](#_ENREF_85)) | Ineligible concept |
| 1. Manglardi 2019 ([86](#_ENREF_86)) | Ineligible article type |
| 1. Masadah 2016 ([87](#_ENREF_87)) | Ineligible article type |
| 1. Maza 2018 ([88](#_ENREF_88)) | Ineligible article type |
| 1. Maza 2018 ([89](#_ENREF_89)) | Ineligible article type |
| 1. McDowell 2017 ([90](#_ENREF_90)) | Ineligible context |
| 1. Melendez 2018 ([91](#_ENREF_91)) | Ineligible article type |
| 1. Mitchell 2015 ([92](#_ENREF_92)) | Ineligible article type |
| 1. Mnisi 2013 ([93](#_ENREF_93)) | Ineligible concept |
| 1. Mohan 2020 ([94](#_ENREF_94)) | Ineligible concept |
| 1. Montealegre 2015 ([95](#_ENREF_95)) | Ineligible context |
| 1. Mremi 2022 ([96](#_ENREF_96)) | Ineligible concept |
| 1. Nct 2014 ([97](#_ENREF_97)) | Ineligible article type |
| 1. Nct 2016 ([98](#_ENREF_98)) | Ineligible context |
| 1. Nct 2018 ([99](#_ENREF_99)) | Ineligible article type |
| 1. NCT01095198 2010 ([100](#_ENREF_100)) | Ineligible article type |
| 1. NCT01316120 2011 ([101](#_ENREF_101)) | Ineligible context |
| 1. NCT02945891 2016 ([102](#_ENREF_102)) | Ineligible context |
| 1. NCT03386695 2017 ([103](#_ENREF_103)) | Ineligible article type |
| 1. NCT03813576 2019 ([104](#_ENREF_104)) | Ineligible article type |
| 1. NCT04612660 2020 ([105](#_ENREF_105)) | Ineligible context |
| 1. NCT05059015 2021 ([106](#_ENREF_106)) | Ineligible article type |
| 1. NCT05600283 2022 ([107](#_ENREF_107)) | Ineligible article type |
| 1. NCT05783167 2023 ([108](#_ENREF_108)) | Ineligible article type |
| 1. Nguyen 2022 ([109](#_ENREF_109)) | Ineligible concept |
| 1. Ni 2021 ([110](#_ENREF_110)) | Ineligible population |
| 1. Nieves 2013 ([111](#_ENREF_111)) | Ineligible article type |
| 1. Nieves-Arriba 2010 ([112](#_ENREF_112)) | Ineligible concept |
| 1. Nishimura 2016 ([113](#_ENREF_113)) | Ineligible article type |
| 1. Nodjikouambaye 2019 ([114](#_ENREF_114)) | Ineligible article type |
| 1. Nwabichie 2018 ([115](#_ENREF_115)) | Ineligible concept |
| 1. Nyabigambo 2022 ([116](#_ENREF_116)) | Ineligible concept |
| 1. Ondryasova 2015 ([117](#_ENREF_117)) | Not in English |
| 1. Oranratanaphan 2020 ([118](#_ENREF_118)) | Ineligible population |
| 1. Osman 2021 ([119](#_ENREF_119)) | Ineligible article type |
| 1. Othman 2014 ([120](#_ENREF_120)) | Ineligible article type |
| 1. Penaranda 2015 ([121](#_ENREF_121)) | Ineligible context |
| 1. Pengsaa 1997 ([122](#_ENREF_122)) | Ineligible concept |
| 1. Phongsavan 2011 ([123](#_ENREF_123)) | Ineligible concept |
| 1. Phoolcharoen 2018 ([124](#_ENREF_124)) | Ineligible concept |
| 1. Poli 2012 ([125](#_ENREF_125)) | Ineligible article type |
| 1. Poli 2020 ([126](#_ENREF_126)) | Ineligible concept |
| 1. Porras 2015 ([127](#_ENREF_127)) | Ineligible context |
| 1. Ratshaa 2018 ([128](#_ENREF_128)) | Ineligible article type |
| 1. Ricard-Gauthier 2015 ([129](#_ENREF_129)) | Ineligible concept |
| 1. Richardson-Parry 2023 ([130](#_ENREF_130)) | Ineligible context |
| 1. Rodrigues 2019 ([131](#_ENREF_131)) | Ineligible concept |
| 1. Rodriguez 2023 ([132](#_ENREF_132)) | Ineligible context |
| 1. Rosen 2018 ([133](#_ENREF_133)) | Ineligible article type |
| 1. Serwadda 1999 ([134](#_ENREF_134)) | Ineligible concept |
| 1. Sharma 2012 ([135](#_ENREF_135)) | Ineligible concept |
| 1. Sherman 2022 ([136](#_ENREF_136)) | Ineligible context |
| 1. Shin 2019 ([137](#_ENREF_137)) | Ineligible context |
| 1. Silas 2018 ([138](#_ENREF_138)) | Ineligible concept |
| 1. Snijders 2013 ([139](#_ENREF_139)) | Ineligible article type |
| 1. Song 2020 ([140](#_ENREF_140)) | Ineligible concept |
| 1. Sormani 2022 ([141](#_ENREF_141)) | Ineligible article type |
| 1. Sun 2020 ([142](#_ENREF_142)) | Ineligible concept |
| 1. Surriabre 2017 ([143](#_ENREF_143)) | Ineligible concept |
| 1. Taylor 2011 ([144](#_ENREF_144)) | Ineligible concept |
| 1. Thasneem 2021 ([145](#_ENREF_145)) | Ineligible article type |
| 1. Tin 2023 ([146](#_ENREF_146)) | Ineligible concept |
| 1. Toliman 2020 ([147](#_ENREF_147)) | Ineligible concept |
| 1. Tshomo 2017 ([148](#_ENREF_148)) | Ineligible concept |
| 1. Chappell 2020 ([149](#_ENREF_149)) | Ineligible article type |
| 1. Untiet 2014 ([150](#_ENREF_150)) | Ineligible concept |
| 1. Vassilakos 2016 ([151](#_ENREF_151)) | Ineligible concept |
| 1. Vega 2022 ([152](#_ENREF_152)) | Inaccessible full text |
| 1. Wang 2014 ([153](#_ENREF_153)) | Ineligible population |
| 1. White 2018 ([154](#_ENREF_154)) | Ineligible article type |
| 1. Wong 2016 ([155](#_ENREF_155)) | Ineligible context |
| 1. Woo 2020 ([156](#_ENREF_156)) | Ineligible article type |
| 1. World Health Organization 2021 ([157](#_ENREF_157)) | Ineligible concept |
| 1. Wu 2021 ([158](#_ENREF_158)) | Ineligible article type |
| 1. Wysong 2018 ([159](#_ENREF_159)) | Ineligible article type |
| 1. Xiong 2023 ([160](#_ENREF_160)) | Ineligible context |
| 1. Xu 2017 ([161](#_ENREF_161)) | Ineligible article type |
| 1. Zhang 2018 ([162](#_ENREF_162)) | Ineligible concept |
| 1. Zhao 2015 ([163](#_ENREF_163)) | Ineligible article type |
| 1. Zhao 2020 ([164](#_ENREF_164)) | Ineligible concept |
| 1. Zhou 2022 ([165](#_ENREF_165)) | Ineligible article type |
| 1. Zhuang 2022 ([166](#_ENREF_166)) | Ineligible concept |
| 1. Braz 2017 ([167](#_ENREF_167)) | Ineligible study type |
| 1. Caleia 2020 ([168](#_ENREF_168)) | Ineligible study type |
| 1. Camara 2021 ([169](#_ENREF_169)) | Ineligible study type |
| 1. Morgan 2019 ([170](#_ENREF_170)) | Ineligible study type |
| 1. Nishimura 2021 ([171](#_ENREF_171)) | Ineligible study type |
| 1. Wong 2018 ([172](#_ENREF_172)) | Ineligible study type |
| 1. Asare 2022 ([173](#_ENREF_173)) | Ineligible study type |
| 1. Kamath Mulki 2021 ([174](#_ENREF_174)) | Ineligible study type |
| 1. Nodjikouambaye 2020 ([175](#_ENREF_175)) | Ineligible study type |
| 1. Tatara 2022 ([176](#_ENREF_176)) | Ineligible study type |
| 1. Arbyn 2014 ([177](#_ENREF_177)) | Ineligible study type |
| 1. Sy 2022 ([178](#_ENREF_178)) | Ineligible study type |
| 1. Zhao 2012 ([179](#_ENREF_179)) | Ineligible study type |
| 1. Malone 2020 ([180](#_ENREF_180)) | Ineligible study type |
| 1. Mezei 2017 ([181](#_ENREF_181)) | Ineligible study type |
| 1. Serrano 2022 ([182](#_ENREF_182)) | Ineligible study type |
| 1. Tesfahunei 2021 ([183](#_ENREF_183)) | Ineligible study type |
| 1. Yeh 2019 ([184](#_ENREF_184)) | Ineligible study type |
| 1. Mekuria 2023 ([185](#_ENREF_185)) | Ineligible study type |
| 1. Consolidated guidelines on HIV, viral hepatitis and STI prevention, diagnosis, treatment and care for key populations (2022) ([186](#_ENREF_186)) | Ineligible article type |
| 1. WHO guideline for screening and treatment of cervical pre-cancer lesions for cervical cancer prevention, second edition (2021) ([187](#_ENREF_187)) | Ineligible article type |
| 1. WHO guideline on self-care Interventions for health and well-being, 2022 revision (2022) ([188](#_ENREF_188)) | Ineligible article type |
| 1. WHO guidelines for screening and treatment of cervical pre-cancer lesions for cervical cancer prevention, second edition: use of mRNA tests for human papillomavirus (HPV) (2021) ([189](#_ENREF_189)) | Ineligible article type |
| 1. WHO Consolidated Guideline on Self-Care Interventions for Health Sexual and Reproductive Health and Rights (2019) ([190](#_ENREF_190)) | Ineligible article type |
| 1. Comprehensive Cervical Cancer Control: A guide to essential practice, Second edition (2014) ([191](#_ENREF_191)) | Ineligible article type |

# **References**

1. Aarnio R., Ostensson E., Olovsson M., Gustavsson I., Gyllensten U. Cost-effectiveness analysis of repeated self-sampling for HPV testing in primary cervical screening: a randomized study. BMC Cancer. 2020;20(1):9 doi:10.1186/s12885-020-07085-9.

2. Actrn. Accuracy and acceptability towards cervical screening by self-collected vs clinician-collected sampling for Human papillomavirus (HPV) DNA testing in female sex workers. 2013.

3. Wong E.L.Y., Chan Paul K. S., Chor Josette s. Y., Cheung Annie W. L., Huang Fenwei, Wong Samuel Y. S. Evaluation of the Impact of Human Papillomavirus DNA Self-sampling on the Uptake of Cervical Cancer Screening. Cancer Nursing. 2016;39(1) doi:10.1097/NCC.0000000000000241.

4. Adegboyega A., Wiggins A.T., Williams L.B., Dignan M. HPV Testing Behaviors and Willingness to Use HPV Self-sampling at Home Among African American (AA) and Sub-Saharan African Immigrant (SAI) Women. J Racial Ethn Health Disparities. 2022;9(6):2485-94 doi:<https://dx.doi.org/10.1007/s40615-021-01184-4>.

5. Adler D.H., Laher F., Lazarus E., Grzesik K., Gray G.E., Allan B., et al. A Viable and Simple Self-Sampling Method for Human Papillomavirus Detection among South African Adolescents. J Immunol Tech Infect Dis. 2013;2(3):18 doi:<https://dx.doi.org/10.4172/2329-9541.1000113>.

6. Adsul P., Srinivas V., Gowda S., Nayaka S., Pramathesh R., Chandrappa K., et al. A community-based, cross-sectional study of hrHPV DNA self-sampling-based cervical cancer screening in rural Karnataka, India. Int J Gynaecol Obstet. 2019;146(2):170-6 doi:<https://dx.doi.org/10.1002/ijgo.12859>.

7. Ajenifuja O.K., Ikeri N.Z., Adeteye O.V., Banjo A.A. Comparison between self sampling and provider collected samples for Human Papillomavirus (HPV) Deoxyribonucleic acid (DNA) testing in a Nigerian facility. Pan Afr Med J. 2018;30:110 doi:<https://dx.doi.org/10.11604/pamj.2018.30.110.14321>.

8. Allende G., Surriabre P., Ovando N., Calle P., Torrico A., Villarroel J., et al. Evaluation of the effectiveness of high-risk human papilloma self-sampling test for cervical cancer screening in Bolivia. BMC Infect Dis. 2020;20(1):259 doi:<https://dx.doi.org/10.1186/s12879-020-04963-2>.

9. Arbyn M., Simon M., de Sanjosé S., Clarke M.A., Poljak M., Rezhake R., et al. Accuracy and effectiveness of HPV mRNA testing in cervical cancer screening: a systematic review and meta-analysis. Lancet Oncology. 2022;23 1077-4114 (Print)(7):950-60 doi:10.1016/S1470-2045(22)00294-7.

10. Avian A., Clemente N., Mauro E., Isidoro E., Di Napoli M., Dudine S., et al. Clinical validation of full HR-HPV genotyping HPV Selfy assay according to the international guidelines for HPV test requirements for cervical cancer screening on clinician-collected and self-collected samples. Journal of Translational Medicine. 2022;20(1):1-12 doi:10.1186/s12967-022-03383-x.

11. Awua A.K., Adanu R.M.K., Wiredu E.K., Afari E.A., Severini A. Differences in age-specific HPV prevalence between self-collected and health personnel collected specimen in a cross-sectional study in Ghana. Infect Agent Cancer. 2017;12:26 doi:<https://dx.doi.org/10.1186/s13027-017-0136-7>.

12. Aziz A., Hamzah H., Lim B.K., Woo Y.L., Omar S.Z. The effectiveness and acceptability of self-sampling against conventional Pap smear in University Malaya Medical Centre (UMMC). BJOG: An International Journal of Obstetrics and Gynaecology. 2012;1):168-9 doi:<https://dx.doi.org/10.1111/j.1471-0528.2012.03380.x>.

13. Bante S.A., Getie S.A., Getu A.A., Mulatu K., Fenta S.L. Uptake of pre-cervical cancer screening and associated factors among reproductive age women in Debre Markos town, Northwest Ethiopia, 2017. BMC Public Health. 2019;19(1):9 doi:10.1186/s12889-019-7398-5.

14. Barbee L., Kobetz E., Menard J., Cook N., Blanco J., Barton B., et al. Assessing the acceptability of self-sampling for HPV among Haitian immigrant women: CBPR in action. Cancer Causes Control. 2010;21(3):421-31 doi:<https://dx.doi.org/10.1007/s10552-009-9474-0>.

15. Baussano I., Tshering S., Choden T., Lazzarato F., Tenet V., Plummer M., et al. Cervical cancer screening in rural Bhutan with the careHPV test on self-collected samples: an ongoing cross-sectional, population-based study (REACH-Bhutan). BMJ Open. 2017;7(7):e016309 doi:<https://dx.doi.org/10.1136/bmjopen-2017-016309>.

16. Beal C.M., Salmeron J., Flores Y.N., Torres L., Granados-Garcia V., Dugan E., et al. Cost analysis of different cervical cancer screening strategies in Mexico. Salud Publica Mex. 2014;56(5):429-501.

17. Belinson J., Qu X., Du H., Wang G., Luo H., Huang X., et al. Potential use of "dry brush" samples for self-collected HPV screening. Journal of Lower Genital Tract Disease. 2017;21(2 Supplement 1):S34-S5.

18. Benski A.C., Viviano M., Jinoro J., Alec M., Catarino R., Herniainasolo J., et al. HPV self-testing for primary cervical cancer screening in Madagascar: VIA/VILI triage compliance in HPV-positive women. PLoS ONE. 2019;14(8):e0220632 doi:<https://dx.doi.org/10.1371/journal.pone.0220632>.

19. Berrada M., Holl R., Ndao T., Benčina G., Dikhaye S., Melhouf A., et al. Healthcare resource utilization and costs associated with anogenital warts in Morocco. Infectious Agents & Cancer. 2021;16(1):1-10 doi:10.1186/s13027-021-00403-1.

20. Bhatla N., Vashist S., Mathur S., Dar L., Rai S., Kumari A., et al. Feasibility and reliability of cervical cancer screening by HPV DNA testing of self-collected samples in a north Indian community. International Journal of Gynecology and Obstetrics. 2015;5):E564-E5.

21. Bidus M.A., Zahn C.M., Maxwell G.L., Rodriguez M., Elkas J.C., Rose G.S. The role of self-collection devices for cytology and human papillomavirus DNA testing in cervical cancer screening. Clinical Obstetrics and Gynecology. 2005;48(1):127-32 doi:<https://dx.doi.org/10.1097/01.grf.0000151569.46072.34>.

22. Bigoni J., Gundar M., Tebeu P.M., Bongoe A., Schäfer S., Fokom-Domgue J., et al. Cervical cancer screening in sub-Saharan Africa: a randomized trial of VIA versus cytology for triage of HPV-positive women. International journal of cancer. 2015;137(1):127‐34 doi:10.1002/ijc.29353.

23. Bohn J.A., Fitch K.C., Currier J.J., Bruegl A. HPV self-collection: what are we waiting for? Exploration of attitudes from frontline healthcare providers. Int J Gynecol Cancer. 2022;32(12):1519-23 doi:10.1136/ijgc-2022-003860.

24. Bottari F., Igidbashian S., Boveri S., Tricca A., Gulmini C., Sesia M., et al. HPV self-sampling in CIN2+ detection: sensitivity and specificity of different RLU cut-off of HC2 in specimens from 786 women. J Clin Pathol. 2017;70(4):327-30 doi:<https://dx.doi.org/10.1136/jclinpath-2016-204044>.

25. Breidenthal A., Van Der Merwe F.H., Dreyer G., Snyman L., Visser C., Botha M.H. Comparison of two self-sampler devices for cervical screening in a South African population. International Journal of Gynecological Cancer. 2019;29(Supplement 4):A560-A1 doi:<https://dx.doi.org/10.1136/ijgc-2019-ESGO.1107>.

26. Brito Y.S., Dominguez Y.S., Leon D.O., Cardella V.K., Diaz A.P., Lay L.A.R., et al. Evaluation of real-time PCR kits to detect high-risk human papillomavirus. [Spanish]. Revista Cubana de Medicina Tropical. 2022;74(1) (no pagination)(e752).

27. Broquet C., Vassilakos P., Ndam Nsangou F.M., Kenfack B., Noubom M., Tincho E., et al. Utility of extended HPV genotyping for the triage of self-sampled HPV-positive women in a screen-and-treat strategy for cervical cancer prevention in Cameroon: a prospective study of diagnostic accuracy. BMJ Open. 2022;12(12):e057234 doi:<https://dx.doi.org/10.1136/bmjopen-2021-057234>.

28. Camel C., Jeronimo J., Holme F., Maldonado F. Screening hard-to-reach women in population-based programs using self-sampling: Experiences from Nicaragua and Guatemala. Journal of Lower Genital Tract Disease. 2017;21(2 Supplement 1):S39.

29. Campos N.G., Castle P.E., Wright T.C., Jr., Kim J.J. Cervical cancer screening in low-resource settings: A cost-effectiveness framework for valuing tradeoffs between test performance and program coverage. Int J Cancer. 2015;137(9):2208-19 doi:<https://dx.doi.org/10.1002/ijc.29594>.

30. Campos N.G., Mvundura M., Jeronimo J., Holme F., Vodicka E., Kim J.J. Cost-effectiveness of HPV-based cervical cancer screening in the public health system in Nicaragua. BMJ Open. 2017;7(6):e015048 doi:<https://dx.doi.org/10.1136/bmjopen-2016-015048>.

31. Carozzi F.M., Yanez R.R., Cannistra S., Matucci M., Paganini I., Sani C., et al. Feasibility of self-sampling and human papillomavirus testing in rural and urban areas for cervical cancer prevention in Bolivia: an observational study. The Lancet Oncology. 2022;23(Supplement 1):S4 doi:<https://dx.doi.org/10.1016/S1470-2045%2822%2900403-X>.

32. Castle P.E., Ajeh R., Dzudie A., Kendowo E., Fuhngwa N., Simo-Wambo A.G., et al. A comparison of screening tests for detection of high-grade cervical abnormalities in women living with HIV from Cameroon. Infect Agent Cancer. 2020;15:45 doi:<https://dx.doi.org/10.1186/s13027-020-00311-w>.

33. Chang C.C., Tseng C.J., Liu W.W., Jain S., Horng S.G., Soong Y.K., et al. Clinical evaluation of a new model of self-obtained method for the assessment of genital human papilloma virus infection in an underserved population. Chang Gung Med J. 2002;25(10):664-71.

34. Chatterjee P., Ray C., Bose S., Banerjee D., Vernekar M., Mandal R. Feasibility of self-sampling for HPV Dna detection in a community-based cervical cancer screening project. Indian Journal of Gynecologic Oncology Conference: 17th World Congress of the International Federation of Colposcopy and Cervical Pathology IFCPC. 2021;19(3) doi:<https://dx.doi.org/10.1007/s40944-021-00548-2>.

35. Chatzistamatiou K., Vrekoussis T., Tsertanidou A., Moysiadis T., Mouchtaropoulou E., Pasentsis K., et al. Acceptability of Self-Sampling for Human Papillomavirus-Based Cervical Cancer Screening. Journal of Women's Health. 2020;29(11):1447-56 doi:<https://dx.doi.org/10.1089/jwh.2019.8258>.

36. Chen Q., Du H., Zhang R., Zhao J.H., Hu Q.C., Wang C., et al. Evaluation of novel assays for the detection of human papilloma virus in self-collected samples for cervical cancer screening. Genet Mol Res. 2016;15(2) doi:10.4238/gmr.15027896.

37. ChiCtr. Accuracy of HPV E6/E7mRNA detection in cervical exfoliated cells self-sampling. 2022.

38. Cho H.W., Lee J.K., Hong J.H. Performance and diagnostic accuracy of human papillomavirus testing on urine and self-collected vaginal samples in a referral population. International Journal of Gynecological Cancer. 2020;30(SUPPL 4):A7 doi:<https://dx.doi.org/10.1136/ijgc-2020-ESGO.15>.

39. Clifford G.M., Baussano I., Heideman D.A.M., Tshering S., Choden T., Lazzarato F., et al. Human papillomavirus testing on self-collected samples to detect high-grade cervical lesions in rural Bhutan: The REACH-Bhutan study. Cancer Med. 2023;12(10):11828-37 doi:<https://dx.doi.org/10.1002/cam4.5851>.

40. Ctri. Cervical cancer screening using HPV self sampling method. 2020.

41. Des Marais A.C.M.P.H., Zhao Y.P., Hobbs M.M.P., Sivaraman V.P., Barclay L.B.A., Brewer N.T.P., et al. Home Self-Collection by Mail to Test for Human Papillomavirus and Sexually Transmitted Infections. Obstetrics & Gynecology. 2018;132(6):1412-20 doi:10.1097/AOG.0000000000002964.

42. Du H., Luo H., Xlan-Zhi D., Belinson J., Qu X., Wang G., et al. Evaluation of vaginal self-samplingwith the cobas 4800 HPV-assay for cervical cancer screening. Journal of Lower Genital Tract Disease. 2017;21(2 Supplement 1):S4.

43. Du H., Duan X., Liu Y., Shi B., Zhang W., Wang C., et al. An evaluation of solid versus liquid transport media for high-risk HPV detection and cervical cancer screening on self-collected specimens. Infectious Agents & Cancer. 2020;15(1):N.PAG-N.PAG doi:10.1186/s13027-020-00333-4.

44. Franciscatto L.G., Silva C.M., Barcellos R.B., Angeli S., Silva M.S., Almeida S.E., et al. Comparison of urine and self-collected vaginal samples for detecting human papillomavirus DNA in pregnant women. Int J Gynaecol Obstet. 2014;125(1):69-72 doi:<https://dx.doi.org/10.1016/j.ijgo.2013.09.031>.

45. Girianelli V.R., Thuler L.C.S., Szklo M., Donato A., Zardo L.M.G., Lozana J.A., et al. Comparison of human papillomavirus DNA tests, liquid-based cytology and conventional cytology for the early detection of cervix uteri cancer. Eur J Cancer Prev. 2006;15(6):504-10 doi:10.1097/01.cej.0000220630.08352.7a.

46. Goldie S.J., Gaffikin L., Goldhaber-Fiebert J.D., Gordillo-Tobar A., Levin C., Mahe C., et al. Cost-effectiveness of cervical-cancer screening in five developing countries. N Engl J Med. 2005;353(20):2158-68 doi:10.1056/NEJMsa044278.

47. Gravitt P.E., Rositch A.F. HPV self-testing and cervical cancer screening coverage. Lancet Oncology. 2014;15 1077-4114 (Print)(2):128-9 doi:10.1016/S1470-2045(13)70605-3.

48. Grigore M., Vasilache I.A., Cianga P., Constantinescu D., Duma O., Matasariu R.D., et al. Acceptability of Human Papilloma Virus Self-Sampling for Cervical Cancer Screening in a Cohort of Patients from Romania (Stage 2). J. 2022;11(9):29 doi:<https://dx.doi.org/10.3390/jcm11092503>.

49. Guillaume D., Chandler R., Igbinoba S. Barriers to cervical cancer screening among women living with hiv in low- and middle-income countries: A systematic review. Journal of the Association of Nurses in AIDS Care. 2020;31(5):497-516 doi:10.1097/JNC.0000000000000194.

50. Guo C.L., Luo H.X., Wang C., Qu X.F., Yang B., Belinson J.L., et al. [Performance of vaginal self-sampling high-risk HPV genotyping as primary and combining cytology or viral load as secondary in cervical cancer screening]. Chung Hua Fu Chan Ko Tsa Chih. 2021;56(4):271-9 doi:<https://dx.doi.org/10.3760/cma.j.cn112141-20200824-00357>.

51. Hailu A., Mariam D.H. Patient side cost and its predictors for cervical cancer in Ethiopia: a cross sectional hospital based study. BMC Cancer. 2013;13(1):69- doi:10.1186/1471-2407-13-69.

52. Hamzah H., Aziz A., Lim B.K., Woo Y.L., Omar S.Z. Evaluation of human papillomavirus (HPV) infection among women in UMMC - comparison between Thin Prep and Fournier's self sampling. Journal of Health and Translational Medicine. 2013;16(SPECIAL):92.

53. Hariprasad R., John A., Abdulkader R.S. Challenges in the Implementation of Human Papillomavirus Self-Sampling for Cervical Cancer Screening in India: A Systematic Review. JCO Glob Oncol. 2023;9:e2200401 doi:<https://dx.doi.org/10.1200/GO.22.00401>.

54. Hernandez-Marquez C.I., Salinas-Urbina A.A., Cruz-Valdez A., Hernandez-Giron C. [Knowledge of human papilloma virus (HPV) and acceptance of vaginal self-sampling among Mexican woman]. Rev Salud Publica (Bogota). 2014;16(5):697-708.

55. Hpv-Dna P., de La G.D. Tamizacion primaria con prueba ADN-VPH en mujeres menores de 30 anos: evaluacion de tecnologia sanitaria. Revista Colombiana de Obstetricia y Ginecologia. 2022;73(2):48-62 doi:<https://dx.doi.org/10.18597/rcog.3866>.

56. Hussain M.J. Home cervical cancer screening a new opportunity for Malaysian women-can self-test kits reduce the burden of cervical cancer in Malaysia? BJOG: An International Journal of Obstetrics and Gynaecology. 2014;6):51 doi:<https://dx.doi.org/10.1111/1471-0528.13165>.

57. Isrctn. Does cervical screening without a speculum increase screening uptake in women aged 50-64? <https://trialsearchwhoint/Trial2aspx?TrialID=ISRCTN16007231>. 2020.

58. Isrctn. Two self-sampling methods (Vaginal dry swabs vs. FTA-elute cartridge) for HPV detection. 2015.

59. Jede F., Brandt T., Gedefaw M., Wubneh S.B., Abebe T., Teka B., et al. Home-based HPV self-sampling assisted by a cloud-based electronic data system: Lessons learnt from a pilot community cervical cancer screening campaign in rural Ethiopia. Papillomavirus Res. 2020;9:100198 doi:<https://dx.doi.org/10.1016/j.pvr.2020.100198>.

60. Jingran L.I., Wu R., Li L., Ling Z., Zhang Z., Liu R., et al. The clinical value of HPV genotyping in triage of women with high-risk HPV-positive self-samples for cervical cancer screening in china. Indian Journal of Gynecologic Oncology Conference: 17th World Congress of the International Federation of Colposcopy and Cervical Pathology IFCPC. 2021;19(3) doi:<https://dx.doi.org/10.1007/s40944-021-00548-2>.

61. Johnson D.C., Bhatta M.P., Smith J.S., Kempf M.C., Broker T.R., Vermund S.H., et al. Assessment of high-risk human papillomavirus infections using clinician- and self-collected cervical sampling methods in rural women from far western Nepal. PLoS ONE. 2014;9(6):e101255 doi:<https://dx.doi.org/10.1371/journal.pone.0101255>.

62. Jun J.K., Lim M.C., Hwang S.H., Shin H.Y., Hwang N.R., Kim Y.J., et al. Comparison of DRY and WET vaginal swabs with cervical specimens in Roche Cobas 4800 HPV and Abbott RealTime High Risk HPV tests. J Clin Virol. 2016;79:80-4 doi:<https://dx.doi.org/10.1016/j.jcv.2016.04.012>.

63. Kahesa C., Thomsen L.T., Linde D.S., McHome B., Katanga J., Swai P., et al. Comparison of human papillomavirus-based cervical cancer screening strategies in Tanzania among women with and without HIV. International Journal of Cancer. 2023;152(4):686-96 doi:<https://dx.doi.org/10.1002/ijc.34283>.

64. Kang L.N., Jeronimo J., Qiao Y.L., Zhao F.H., Chen W., Valdez M., et al. Optimal positive cutoff points for careHPV testing of clinician- and self-collected specimens in primary cervical cancer screening: an analysis from rural China. J Clin Microbiol. 2014;52(6):1954-61 doi:<https://dx.doi.org/10.1128/JCM.03432-13>.

65. Keane A., Ng C.W., Simms K.T., Nguyen D., Woo Y.L., Saville M., et al. The road to cervical cancer elimination in Malaysia: Evaluation of the impact and cost-effectiveness of human papillomavirus screening with self-collection and digital registry support. Int J Cancer. 2021;149(12):1997-2009 doi:<https://dx.doi.org/10.1002/ijc.33759>.

66. Khanna N., Mishra S.I., Tian G., Tan M.T., Arnold S., Lee C., et al. Human papillomavirus detection in self-collected vaginal specimens and matched clinician-collected cervical specimens. Int J Gynecol Cancer. 2007;17(3):615-22.

67. Khoo S., Lim W., Nasir N., Gravitt P., Woo Y.L. Would a multi-ethnic asian female population take up self-sampling HPV testing as a routine cervical screening method? International Journal of Gynecological Cancer. 2020;30(SUPPL 3):A32 doi:<https://dx.doi.org/10.1136/ijgc-2020-IGCS.50>.

68. Kim M.H., Jung H.J., Park S.I., Kim B.J. Self‐obtained vaginal samples for HPV DNA testing to detect HPV‐related cervical disease. International Journal of Gynecology & Obstetrics. 2021;154(1):127-32 doi:10.1002/ijgo.13574.

69. Klischke L., von Ehr J., Kohls F., Kampers J., Hulse F., Schmitz M., et al. Performance of a six-methylation-marker assay on self-collected cervical samples-A feasibility study. J Virol Methods. 2021;295:6 doi:10.1016/j.jviromet.2021.114219.

70. Kovalyova Z.A. European Cancer Summit 2022 submission for HPV Action NetworkHPV SCREENING FOR CERVICAL CANCER USING THE SELF-SAMPLING MODEL IN UKRAINE DURING THE WAR. Journal of Cancer Policy. 2023;Conference: European Cancer Summit 2022. Brussels Belgium. 35 (no pagination)(100391) doi:<https://dx.doi.org/10.1016/j.jcpo.2023.100391>.

71. Krings A., Effah K., Amuah J.E., Dunyo P., Hansen B., Adaletey R., et al. Comparison of delphi screener and evalyn brush self-samplers for HPV screening in low resource settings (accessing∗). Oncology research and treatment. 2016;39:98‐9 doi:10.1159/000444354.

72. Kuhn L., Saidu R., Boa R., Denny L., Svanholm-Barrie C., Tergas A., et al. HPV testing of self-collected vaginal swabs for cervical cancer prevention in South Africa. Journal of Global Oncology. 2016;2(3 Supplement 3):65s-6s doi:<https://dx.doi.org/10.1200/JGO.2016.003749>.

73. Kuhn L. Changing cycle threshold cut-offs on point-of-care HPV test for screen-and-treat programs in South Africa. International Journal of Gynecological Cancer. 2018;28(Supplement 2):29 doi:<https://dx.doi.org/10.1097/01.IGC.0000546279.09648.02>.

74. Kuriakose S., Sabeena S., Binesh D., Abdulmajeed J., Ravishankar N., Ramachandran A., et al. Diagnostic accuracy of self-collected vaginal samples for HPV DNA detection in women from South India. International journal of gynaecology and obstetrics: the official organ of the International Federation of Gynaecology and Obstetrics. 2020;09 doi:<https://dx.doi.org/10.1002/ijgo.13116>.

75. Latiff L.A., Rahman S.A., Wee W.Y., Dashti S., Andi Asri A.A., Unit N.H., et al. Assessment of the reliability of a novel self-sampling device for performing cervical sampling in Malaysia. Asian Pac J Cancer Prev. 2015;16(2):559-64.

76. Lazcano-Ponce E., Lorincz A.T., Cruz-Valdez A., Salmeron J., Uribe P., Velasco-Mondragon E., et al. Self-collection of vaginal specimens for human papillomavirus testing in cervical cancer prevention (MARCH): a community-based randomised controlled trial. Lancet. 2011;378(9806):1868-73 doi:<https://dx.doi.org/10.1016/S0140-6736(11)61522-5>.

77. Lazcano-Ponce E., Lorincz A.T., Torres L., Salmeron J., Cruz A., Rojas R., et al. Specimen self-collection and HPV DNA screening in a pilot study of 100,242 women. Int J Cancer. 2014;135(1):109-16 doi:<https://dx.doi.org/10.1002/ijc.28639>.

78. Le D., Coriolan Ciceron A., Jeon M.J., Gonzalez L.I., Jordan J.A., Bordon J., et al. Cervical Cancer Prevention and High-Risk HPV Self-Sampling Awareness and Acceptability among Women Living with HIV: A Qualitative Investigation from the Patients' and Providers' Perspectives. Current Oncology. 2022;29(2):516-33 doi:10.3390/curroncol29020047.

79. Lee F., Bula A., Chapola J., Mapanje C., Phiri B., Kamtuwange N., et al. Women's experiences in a community-based screen-and-treat cervical cancer prevention program in rural Malawi: a qualitative study. BMC Cancer. 2021;21(1):428 doi:<https://dx.doi.org/10.1186/s12885-021-08109-8>.

80. Liling C., Shirley H.F.L., Nurul Iffah Hazwani J.a., Edwin L., Roslin S. Reasons for non-attendance to cervical cancer screening and acceptability of HPV self-sampling among Bruneian women: A cross-sectional study. medRxiv. 2022:2021.03.30.21254670 doi:10.1101/2021.03.30.21254670.

81. Lim J.N.W., Ojo A.A. Barriers to utilisation of cervical cancer screening in Sub Sahara Africa: a systematic review. Eur J Cancer Care. 2017;26(1):9 doi:10.1111/ecc.12444.

82. Lin C.Q., Zeng X., Cui J.F., Liao G.D., Wu Z.N., Gao Q.Q., et al. Stability Study of Cervical Specimens Collected by Swab and Stored Dry Followed by Human Papillomavirus DNA Detection Using the cobas 4800 Test. J Clin Microbiol. 2017;55(2):568-73 doi:<https://dx.doi.org/10.1128/JCM.02025-16>.

83. Lince-Deroche N., Phiri J., Michelow P., Smith J.S., Firnhaber C. Costs and cost effectiveness of three approaches for cervical cancer screening among HIV-positive women in Johannesburg, South Africa. PLoS ONE. 2015;10(11) (no pagination)(e0141969) doi:<https://dx.doi.org/10.1371/journal.pone.0141969>.

84. Lorenzi A.T., Fregnani J.H., Possati-Resende J.C., Neto C.S., Villa L.L., Longatto-Filho A. Self-collection for high-risk HPV detection in Brazilian women using the careHPV™ test. Gynecologic oncology. 2013;131(1):131‐4 doi:10.1016/j.ygyno.2013.07.092.

85. Mandelblatt J.S., Lawrence W.F., Gaffikin L., Limpahayom K.K., Lumbiganon P., Warakamin S., et al. Costs and benefits of different strategies to screen for cervical cancer in less-developed countries. J Natl Cancer Inst. 2002;94(19):1469-83.

86. Manglardi E.E., Lendore J., Balhotra K., Fields P.J., Bahadoor-Yetman A., McGill F.M. Self Screening as an Alternative Method of Cervical Screening. Obstetrics and gynecology. 2019;133(SUPPL 1) doi:10.1097/01.AOG.0000558962.45537.e0.

87. Masadah R., Chandra R., Rauf S. The sensitivity and specificity of human papilloma virus self-sampling collection test in cervical cytology screening. International Journal of Gynecological Cancer. 2016;26(Supplement 3):402 doi:<https://dx.doi.org/10.1097/01.IGC.0000503327.50238.5c>.

88. Maza M., Melendez M., Cremer M., Masch R., Alonzo T., Castle P., et al. High acceptability of human papillomavirus self-sampling among nonattenders of a public cervical cancer screening program in El Salvador. Journal of Global Oncology. 2018;4(Supplement 1):13S doi:<https://dx.doi.org/10.1200/jgo.18.50000>.

89. Maza M., Melendez M., Masch R., Alonzo T., Castle P., Soler M., et al. Hpv self-sampling in non-attenders of cervical cancer screening programs in El Salvador. International Journal of Gynecology and Obstetrics. 2018;143(Supplement 3):441 doi:<https://dx.doi.org/10.1002/ijgo.12582>.

90. McDowell M., Pardee D.J., Peitzmeier S., Reisner S.L., Agenor M., Alizaga N., et al. Cervical Cancer Screening Preferences Among Trans-Masculine Individuals: Patient-Collected Human Papillomavirus Vaginal Swabs Versus Provider-Administered Pap Tests. LGBT Health. 2017;4(4):252-9 doi:10.1089/lgbt.2016.0187.

91. Melendez M., Herrera A., Hernandez X., Rodryguez B., Soler M., Alfaro K., et al. Cervical cancer screening in transgender men in El Salvador: A pilot study. Journal of Global Oncology. 2018;4(Supplement 1):12S doi:<https://dx.doi.org/10.1200/jgo.18.40000>.

92. Mitchell S., Moses E., Pedersen H., Sekikubo M., Mwesigwa D., Singer J., et al. EXAMINING HPV PREVALENCE AND UPTAKE OF CERVICAL CANCER SCREENING AMONG HIV POSITIVE AND NEGATIVE WOMEN PARTICIPATING IN A PILOT RCT IN UGANDA COMPARING SELF-COLLECTION BASED HPV TESTING TO VIA. Sex Transm Infect. 2015;91:A66-A7 doi:10.1136/sextrans-2015-052270.180.

93. Mnisi E.F., Dreyer G., Richter K.L., Horton A., Snyman L.C. Human papillomavirus DNA testing on self-collected vaginal tampon samples as a cervical cancer screening test in a Gauteng population. Southern African Journal of Gynaecological Oncology. 2013;5(2 Supplement):S15-S20 doi:10.1080/20742835.2013.11441217.

94. Mohan G., Chattopadhyay S. Cost-effectiveness of Leveraging Social Determinants of Health to Improve Breast, Cervical, and Colorectal Cancer Screening A Systematic Review. JAMA Oncol. 2020;6(9):1434-44 doi:10.1001/jamaoncol.2020.1460.

95. Montealegre J.R., Landgren R.M., Anderson M.L., Hoxhaj S., Williams S., Robinson D.J., et al. Acceptability of self-sample human papillomavirus testing among medically underserved women visiting the emergency department. Gynecol Oncol. 2015;138(2):317-22 doi:<https://dx.doi.org/10.1016/j.ygyno.2015.05.028>.

96. Mremi A., McHome B., Mlay J., Schledermann D., Blaakaer J., Rasch V. Performance of HPV testing, Pap smear and VIA in women attending cervical cancer screening in Kilimanjaro region, Northern Tanzania: a cross-sectional study nested in a cohort. BMJ Open. 2022;12(10):e064321 doi:<https://dx.doi.org/10.1136/bmjopen-2022-064321>.

97. Nct. ASPIRE Pilot: comparing Self-collected HPV Testing With Visual Inspection With Acetic Acid Screening for Cervical Cancer. <https://clinicaltrialsgov/show/NCT02029794>. 2014.

98. Nct. A Comparison of Cotton and Flocked Swabs for Vaginal Self Collection. <https://clinicaltrialsgov/show/NCT02785289>. 2016.

99. Nct. Acceptability of Human Papillomavirus (HPV) Self-sampling Tools for Cervical Cancer Prevention. <https://clinicaltrialsgov/show/NCT03613493>. 2018.

100. Nct. Randomized Trial of Vaginal Self Sampling for Human Papillomavirus (HPV). 2010.

101. Nct. Randomized Comparison of Vaginal Self Sampling for Human Papillomavirus (HPV) Testing by Standard Versus Dry Vaginal Swabs. 2011.

102. Nct. SElf-SAMpling in Cervical Cancer Screening; SESAM Study. 2016.

103. Nct. Acceptability and Validity of Self Sampling for High Risk HPV Detection Among Women in Maharashtra. 2017.

104. Nct. Acceptability and Efficacy of Self-sampling for Cervical Cancer Screening: A Pilot Study. 2019.

105. Nct. HPV Self-test to Increase Cervical Cancer Screening in Asian Women. 2020.

106. Nct. Human Papilloma Virus (HPV) Self-collection and Women Adherence. 2021.

107. Nct. Patient Self-sampling of HPV to Screen for Cervical Cancer. 2022.

108. Nct. Self-collected Vaginal and Urine Samples in HIV-positive Women. 2023.

109. Nguyen D.T.N., Simms K.T., Keane A., Mola G., Bolnga J.W., Kuk J., et al. Towards the elimination of cervical cancer in low-income and lower-middle-income countries: modelled evaluation of the effectiveness and cost-effectiveness of point-of-care HPV self-collected screening and treatment in Papua New Guinea. BMJ glob. 2022;7(3):03 doi:<https://dx.doi.org/10.1136/bmjgh-2021-007380>.

110. Ni Y., Lu Y., He X., Li Y., Xu C., Tang W. Self-sampling demonstrates comparable sensitivity and specificity to clinician-sampling for HPV testing among msm in china. Sexually Transmitted Infections. 2021;97(SUPPL 1):A105 doi:<https://dx.doi.org/10.1136/sextrans-2021-sti.275>.

111. Nieves L., Enerson C.L., Belinson S., Brainard J., Chiesa-Vottero A., Nagore N., et al. Primary cervical cancer screening and triage using an mRNA human papillomavirus assay and visual inspection. Int J Gynecol Cancer. 2013;23(3):513-8 doi:<https://dx.doi.org/10.1097/IGC.0b013e318280f3bc>.

112. Nieves-Arriba L., Enerson C., Belinson S., Booth C., Brainard J., Chiesa-Vottero A., et al. Mexican cervical cancer screening study II: Acceptability of a new self-sampling device. Gynecologic Oncology. 2010;1):S18-S9 doi:<https://dx.doi.org/10.1016/j.ygyno.2009.12.013>.

113. Nishimura H., Yatich N., Huchko M. Facilitators and barriers to cervical cancer screening in Migori County, Kenya. Journal of Global Oncology. 2016;2(3 Supplement 3):82s doi:<https://dx.doi.org/10.1200/JGO.2016.003970>.

114. Nodjikouambaye Z.A., Sadjoli D., Bouassa R.S.M., Pere H., Veyer D., Adawaye C., et al. Accuracy of cervical cancer screening using a self-collected vial for HPV DNA testing among adult women in sub-Saharan Africa. Sexually Transmitted Infections. 2019;95(Supplement 1):A346-A7 doi:<https://dx.doi.org/10.1136/sextrans-2019-sti.869>.

115. Nwabichie C.C., Manaf R.A., Ismail S.B. Factors Affecting Uptake of Cervical Cancer Screening Among African Women in Klang Valley, Malaysia. Asian Pacific journal of cancer prevention. 2018;19(3):825‐31 doi:10.22034/APJCP.2018.19.3.825.

116. Nyabigambo A., Mayega R.W., Mendoza H., Shiraz A., Doorbar J., Atuyambe L., et al. The preference of women living with HIV for the HPV self-sampling of urine at a rural HIV clinic in Uganda. S. 2022;37(1):414 doi:<https://dx.doi.org/10.4102/sajid.v37i1.414>.

117. Ondryasova H., Koudelakova V., Drabek J., Vanek P., Slavkovsky R., Hajduch M. [Utilization of self-sampling kits for HPV testing in cervical cancer screening - pilot study]. Ceska Gynekol. 2015;80(6):436-43.

118. Oranratanaphan S., Kengsakul M., Triratanachat S., Kitkumthorn N., Mutirangura A., Termrungruanglert W. CyclinA1 Promoter Methylation in Self-Sampling Test. Asian Pac J Cancer Prev. 2020;21(10):2913-7 doi:<https://dx.doi.org/10.31557/APJCP.2020.21.10.2913>.

119. Osman N. Cervical cancer screening: Benefits and challenges of Hpv self-sampling in Mozambique. Sexually Transmitted Infections. 2021;97(SUPPL 1):A14 doi:<https://dx.doi.org/10.1136/sextrans-2021-sti.46>.

120. Othman N.H., Zaki F.H.M. Self-Collection Tools for Routine Cervical Cancer Screening: A Review. Asian Pac J Cancer Prev. 2014;15(20):8563-9 doi:10.7314/apjcp.2014.15.20.8563.

121. Penaranda E., Molokwu J., Flores S., Byrd T., Brown L., Shokar N. Women's Attitudes Toward Cervicovaginal Self-Sampling for High-Risk HPV Infection on the US-Mexico Border. Journal of Lower Genital Tract Disease. 2015;19(4):323-8 doi:10.1097/LGT.0000000000000134.

122. Pengsaa P., Vatanasapt V., Sriamporn S., Sanchaisuriya P., Schelp F.P., Noda S., et al. A self-administered device for cervical cancer screening in Northeast Thailand. Acta Cytologica. 1997;41(3):749-54 doi:<http://dx.doi.org/10.1159/000332698>.

123. Phongsavan K., Phengsavanh A., Wahlstrom R., Marions L. Safety, feasibility, and acceptability of visual inspection with acetic acid and immediate treatment with cryotherapy in rural Laos. International Journal of Gynecology and Obstetrics. 2011;114(3):268-72 doi:<https://dx.doi.org/10.1016/j.ijgo.2011.03.009>.

124. Phoolcharoen N., Kantathavorn N., Krisorakun W., Sricharunrat T., Teerayathanakul N., Taepisitpong C., et al. Agreement of self- and physician-collected samples for detection of high-risk human papillomavirus infections in women attending a colposcopy clinic in Thailand. BMC Res Notes. 2018;11(1):136 doi:<https://dx.doi.org/10.1186/s13104-018-3241-9>.

125. Poli U.R. cAREHPVTM experience in four countries. International Journal of Gynecology and Obstetrics. 2012;3):S235-S6 doi:<https://dx.doi.org/10.1016/S0020-7292%2812%2960325-7>.

126. Poli U.R., Muwonge R., Bhoopal T., Lucas E., Basu P. Feasibility, Acceptability, and Efficacy of a Community Health Worker-Driven Approach to Screen Hard-to-Reach Periurban Women Using Self-Sampled HPV Detection Test in India. JCO Glob Oncol. 2020;6:658-66 doi:<https://dx.doi.org/10.1200/GO.20.00061>.

127. Porras C., Hildesheim A., Gonzalez P., Schiffman M., Rodriguez A.C., Wacholder S., et al. Performance of self-collected cervical samples in screening for future precancer using human papillomavirus DNA testing. J Natl Cancer Inst. 2015;107(1):400 doi:<https://dx.doi.org/10.1093/jnci/dju400>.

128. Ratshaa B., Shissler T., Varallo J., Dialwa R., Bertram M.M., Bazant E., et al. Acceptability of HPV self collection: A demonstration project in Botswana. International Journal of Gynecology and Obstetrics. 2018;143(Supplement 3):425 doi:<https://dx.doi.org/10.1002/ijgo.12582>.

129. Ricard-Gauthier D., Wisniak A., Catarino R., Faure van Rossum A., Meyer-Hamme U., Negulescu R., et al. Use of Smartphones as Adjuvant Tools for Cervical Cancer Screening in Low-Resource Settings. Journal of Lower Genital Tract Disease. 2015;19(4):295-300 doi:10.1097/LGT.0000000000000136.

130. Richardson-Parry A., Silva M., Valderas J.M., Donde S., Woodruff S., van Vugt J. Interactive or tailored digital interventions to increase uptake in cervical, breast, and colorectal cancer screening to reduce health inequity: a systematic review. Eur J Cancer Prev. 2023;32(4):396-409 doi:10.1097/cej.0000000000000796.

131. Rodrigues L.L., Pilotto J.H., Lima L.R., Gaydos C.A., Hardick J., Morgado M.G., et al. Self-collected versus clinician-collected samples for HSV-2 and HSV-2/HPV screening in HIV-infected and -uninfected women in the Tapajos region, Amazon, Brazil. Int J STD AIDS. 2019;30(11):1055-62 doi:<https://dx.doi.org/10.1177/0956462419842007>.

132. Rodriguez N.M., Brennan L.P., Claure L., Balian L.N., Champion V.L., Forman M.R. Leveraging COVID-era innovation for cervical cancer screening: Clinician awareness and attitudes toward self-sampling and rapid testing for HPV detection. PLoS ONE. 2023;18(3):e0282853 doi:<https://dx.doi.org/10.1371/journal.pone.0282853>.

133. Rosen B., Namugga J., Orang'o E., Itsura P., Tonui P., Ganda G., et al. Accuracy of self versus provider collected vaginal/cervical samples to detect HR HPV in HIV affected and unaffected women in Kenya. International Journal of Gynecological Cancer. 2018;28(Supplement 2):495 doi:<https://dx.doi.org/10.1097/01.IGC.0000546279.09648.02>.

134. Serwadda D., Wawer M.J., Shah K.V., Sewankambo N.K., Daniel R., Li C., et al. Use of a hybrid capture assay of self-collected vaginal swabs in rural Uganda for detection of human papillomavirus. J Infect Dis. 1999;180(4):1316-9.

135. Sharma M., Ortendahl J., van der Ham E., Sy S., Kim J.J. Cost-effectiveness of human papillomavirus vaccination and cervical cancer screening in Thailand. Bjog. 2012;119(2):166-76 doi:<https://dx.doi.org/10.1111/j.1471-0528.2011.02974.x>.

136. Sherman S.M., Brewer N., Bartholomew K., Bromhead C., Crengle S., Cunningham C., et al. Human papillomavirus self-testing among unscreened and under-screened Māori, Pasifika and Asian women in Aotearoa New Zealand: a preference survey among responders and interviews with clinical-trial nonresponders. Health expectations. 2022;25(6):2914‐23 doi:10.1111/hex.13599.

137. Shin H.Y., Lee B., Hwang S.H., Lee D.O., Sung N.Y., Park J.Y., et al. Evaluation of satisfaction with three different cervical cancer screening modalities: clinician-collected Pap test vs. HPV test by self-sampling vs. HPV test by urine sampling. J. 2019;30(5):e76 doi:<https://dx.doi.org/10.3802/jgo.2019.30.e76>.

138. Silas O.A., Achenbach C.J., Murphy R.L., Hou L.F., Sagay S.A., Banwat E., et al. Cost effectiveness of human papilloma virus vaccination in low and middle income countries: a systematic review of literature. Expert Rev Vaccines. 2018;17(1):91-8 doi:10.1080/14760584.2018.1411195.

139. Snijders P.J.F., Verhoef V.M.J., Arbyn M., Ogilvie G., Minozzi S., Banzi R., et al. High-risk HPV testing on self-sampled versus clinician-collected specimens: A review on the clinical accuracy and impact on population attendance in cervical cancer screening. Int J Cancer. 2013;132(10):2223-36 doi:10.1002/ijc.27790.

140. Song F., Du H., Wang C., Huang X., Wu R. The effectiveness of HPV16 and HPV18 genotyping and cytology with different thresholds for the triage of human papillomavirus-based screening on self-collected samples. PLoS ONE. 2020;15(6):e0234518 doi:<https://dx.doi.org/10.1371/journal.pone.0234518>.

141. Sormani J., Wisniak A., Kenfack B., Datchoua A.M., Vassilakos P., Petignat P., et al. Cost-Effectiveness of Cervical Cancer Screnning Strategies among Women in Cameroon. International Journal of Gynecological Cancer. 2022;32(Supplement 2):A13-A4 doi:<https://dx.doi.org/10.1136/ijgc-2022-ESGO.29>.

142. Sun J., Wu S., Hu L., Shang H., Yang Y., Pretorius R., et al. Evaluation of Liquid Versus Dry Specimen Transport With a Newly Validated Isothermal Amplification High-Risk Human Papillomavirus Assay. J. 2020;24(3):243-6 doi:<https://dx.doi.org/10.1097/LGT.0000000000000546>.

143. Surriabre P., Allende G., Prado M., Caceres L., Bellot D., Torrico A., et al. Self-sampling for human papillomavirus DNA detection: a preliminary study of compliance and feasibility in BOLIVIA. BMC Womens Health. 2017;17(1):135 doi:<https://dx.doi.org/10.1186/s12905-017-0490-z>.

144. Taylor S., Wang C., Wright T.C., Denny L., Kuhn L. A comparison of human papillomavirus testing of clinician-collected and self-collected samples during follow-up after screen-and-treat. Int J Cancer. 2011;129(4):879-86 doi:<https://dx.doi.org/10.1002/ijc.25731>.

145. Thasneem P., Nalini C., Rani S.S., Bharathipriya J., Sridharan V., Balasubramani L. The use of HPV self-sampling to increase follow-up rate: A feasibility study. Indian Journal of Gynecologic Oncology Conference: 17th World Congress of the International Federation of Colposcopy and Cervical Pathology IFCPC. 2021;19(3) doi:<https://dx.doi.org/10.1007/s40944-021-00548-2>.

146. Tin K.N., Ngamjarus C., Rattanakanokchai S., Sothornwit J., Aue-Aungkul A., Paing A.K., et al. Interventions to increase the uptake of cervical cancer screening in low- and middle-income countries: a systematic review and meta-analysis. BMC Womens Health. 2023;23(1):120 doi:<https://dx.doi.org/10.1186/s12905-023-02265-8>.

147. Toliman P.J., Phillips S., de Jong S., O'Neill T., Tan G., Brotherton J.M.L., et al. Evaluation of p16/Ki-67 dual-stain cytology performed on self-collected vaginal and clinician-collected cervical specimens for the detection of cervical pre-cancer. Clin Microbiol Infect. 2020;26(6):748-52 doi:<https://dx.doi.org/10.1016/j.cmi.2019.10.020>.

148. Tshomo U., Franceschi S., Tshokey T., Tobgay T., Baussano I., Tenet V., et al. Evaluation of the performance of Human Papillomavirus testing in paired urine and clinician-collected cervical samples among women aged over 30 years in Bhutan. Virology Journal. 2017;14(1) (no pagination)(74) doi:<https://dx.doi.org/10.1186/s12985-017-0744-2>.

149. Chappell N. The Selfie Study- Assessing Novel Markers for Cervical Cancer Screening From Self-collected Samples. <https://classic.clinicaltrials.gov/show/NCT04423679>; 2020.

150. Untiet S., Vassilakos P., McCarey C., Tebeu P.M., Kengne-Fosso G., Menoud P.A., et al. HPV self-sampling as primary screening test in sub-Saharan Africa: implication for a triaging strategy. Int J Cancer. 2014;135(8):1911-7 doi:<https://dx.doi.org/10.1002/ijc.28834>.

151. Vassilakos P., Catarino R., Bougel S., Munoz M., Benski C., Meyer-Hamme U., et al. Use of swabs for dry collection of self-samples to detect human papillomavirus among Malagasy women. Infect Agent Cancer. 2016;11:13 doi:<https://dx.doi.org/10.1186/s13027-016-0059-8>.

152. Vega B., Neira V.A., Ortiz J., Orellana M.P., Lopez D., Carreno T.P., et al. Comparison of Acceptance, Sensitivity and Specificity among Urine Sampling, Self-Sampling and Clinician Sampling. Journal of Lower Genital Tract Disease. 2022;26(2 SUPPL 1):S3 doi:<https://dx.doi.org/10.1097/LGT.0000000000000670>.

153. Wang S.M., Hu S.Y., Chen F., Chen W., Zhao F.H., Zhang Y.Q., et al. Clinical evaluation of human papillomavirus detection by careHPV TM test on physician-samples and self-samples using the indicating FTA Elute R card. Asian Pac J Cancer Prev. 2014;15(17):7085-9.

154. White H.L., Ndamaje F., Kaggwa M., Blumenthal P.D. Optimizing community-based 'screen and treat' models with HPV testing in Uganda. International Journal of Gynecology and Obstetrics. 2018;143(Supplement 3):445 doi:<https://dx.doi.org/10.1002/ijgo.12582>.

155. Wong E.L.Y., Chan P.K.S., Chor J.S.Y., Cheung A.W.L., Huang F., Wong S.Y.S. Evaluation of the Impact of Human Papillomavirus DNA Self-sampling on the Uptake of Cervical Cancer Screening. Cancer Nursing. 2016;39(1):E1-E11 doi:10.1097/NCC.0000000000000241.

156. Woo Y.L. The feasibility and acceptability of self-sampling and HPV testing using Cepheid Xpert HPV in a busy primary care facility. Journal of Virus Eradication. 2020;5:10-1 doi:<https://dx.doi.org/10.1016/S2055-6640%2820%2930160-6>.

157. World Health O. Consolidated guidelines on HIV prevention, testing, treatment, service delivery and monitoring: recommendations for a public health approach. Geneva: World Health Organization; 2021.

158. Wu L.J., Wei L., Zhang Z. Feasibility and triage study of hpv genotyping of self-sampling in cervical cancer screening on internet-based in china. Indian Journal of Gynecologic Oncology Conference: 17th World Congress of the International Federation of Colposcopy and Cervical Pathology IFCPC. 2021;19(3) doi:<https://dx.doi.org/10.1007/s40944-021-00548-2>.

159. Wysong M. High HPV prevalence on self-collected vaginal samples in botswana. International Journal of Gynecology and Obstetrics. 2018;143(Supplement 3):439 doi:<https://dx.doi.org/10.1002/ijgo.12582>.

160. Xiong S., Ghebre R., Kulasingam S., Mason S.M., Pratt R.J., Lazovich D.A. Exploring factors associated with preferences for human papillomavirus (HPV) self-sampling among racially-and ethnically-diverse women: A cross-sectional study. Cancer Prevention Research Conference: AACR Special Conference: Precision Prevention, Early Detection, and Interception of Cancer Austin, TX United States. 2023;16(Supplement 1) doi:<https://dx.doi.org/10.1158/1940-6215.PrecPrev22-P026>.

161. Xu X.Q., Zhang L., Hu S.Y., Chen F., Zhang X., Pan Q.J., et al. Longitudinal performance of self-collected human papilloma virus testing and other mainstream strategies in detecting prevalent and incident cervical precancer: A 15-year cohort study in china. The Lancet. 2017;390(SPEC.ISS 1):64.

162. Zhang L., Xu X.Q., Hu S.Y., Chen F., Zhang X., Pan Q.J., et al. Durability of clinical performance afforded by self-collected HPV testing: A 15-year cohort study in China. Gynecol Oncol. 2018;151(2):221-8 doi:<https://dx.doi.org/10.1016/j.ygyno.2018.09.012>.

163. Zhao Y., Chen F., Zhang X., Zhao F., Gao G., Zheng F., et al. A real world feasibility study for using HPV test as primary screening technology for cervical cancer screening in rural China. Annals of Global Health. 2015;81(1):84.

164. Zhao X.L., Xu X.Q., Duan X.Z., Rezhake R., Hu S.Y., Wang Y., et al. Comparative performance evaluation of different HPV tests and triaging strategies using self-samples and feasibility assessment of thermal ablation in 'colposcopy and treat' approach: A population-based study in rural China. Int J Cancer. 2020;147(5):1275-85 doi:<https://dx.doi.org/10.1002/ijc.32881>.

165. Zhou D., Liu Z., Wang W., Zhao M., Tang W., Gong F. EE221 Cost-Effectiveness of Cervical Cancer Screening Program in China. Value in Health. 2022;25(7 Supplement):S377 doi:<https://dx.doi.org/10.1016/j.jval.2022.04.469>.

166. Zhuang L.J., Weng X.L., Wang L.H., Xie X.Y., Zhong L.Y., Liu D.B., et al. Performance of the Human Papillomavirus E6/E7 mRNA Assay in the Primary Screening of Cervical Cancer: Opportunistic Screening in Fujian, China. Int J Womens Health. 2022;14:1519-30 doi:10.2147/ijwh.S383431.

167. Braz N., Lorenzi N.P.C., Sorpreso I.C.E., de Aguiar L.M., Baracat E.C., Soares J.M. The acceptability of vaginal smear self-collection for screening for cervical cancer: a systematic review. Clinics. 2017;72(3):183-7 doi:10.6061/clinics/2017(03)09.

168. Caleia A.I., Pires C., Pereira J.D., Pinto-Ribeiro F., Longatto A. Self-Sampling as a Plausible Alternative to Screen Cervical Cancer Precursor Lesions in a Population with Low Adherence to Screening: A Systematic Review. Acta Cytol. 2020;64(4):332-43 doi:10.1159/000505121.

169. Camara H., Zhang Y., Lafferty L., Vallely A.J., Guy R., Kelly-Hanku A. Self-collection for HPV-based cervical screening: a qualitative evidence meta-synthesis. BMC Public Health. 2021;21(1):1503 doi:<https://dx.doi.org/10.1186/s12889-021-11554-6>.

170. Morgan K., Azzani M., Si Lay K., Yut-Lin W., Tin Tin S., Khaing S.L., et al. Acceptability of Women Self-Sampling versus Clinician-Collected Samples for HPV DNA Testing: A Systematic Review. Journal of Lower Genital Tract Disease. 2019;23(3):193-9 doi:10.1097/LGT.0000000000000476.

171. Nishimura H., Yeh P.T., Oguntade H., Kennedy C.E., Narasimhan M. HPV self-sampling for cervical cancer screening: a systematic review of values and preferences. BMJ glob. 2021;6(5):05 doi:<https://dx.doi.org/10.1136/bmjgh-2020-003743>.

172. Wong J.P.H., Vahabi M., Miholjcic J., Tan V., Owino M., Li A.T.W., et al. Knowledge of HPV/cervical cancer and acceptability of HPV self-sampling among women living with HIV: A scoping review. Curr. 2018;25(1):e73-e82 doi:<https://dx.doi.org/10.3747/co.25.3855>.

173. Asare M., Abah E., Obiri-Yeboah D., Lowenstein L., Lanning B. HPV Self-Sampling for Cervical Cancer Screening among Women Living with HIV in Low- and Middle-Income Countries: What Do We Know and What Can Be Done? Healthcare (2227-9032). 2022;10(7):1270- doi:10.3390/healthcare10071270.

174. Kamath Mulki A., Withers M. Human Papilloma Virus self-sampling performance in low- and middle-income countries. BMC Women's Health. 2021;21(1):1-11 doi:10.1186/s12905-020-01158-4.

175. Nodjikouambaye Z.A., Adawaye C., Mboumba Bouassa R.S., Sadjoli D., Belec L. A systematic review of self-sampling for HPV testing in Africa. Int J Gynaecol Obstet. 2020;149(2):123-9 doi:<https://dx.doi.org/10.1002/ijgo.13112>.

176. Tatara T., Wnuk K., Miazga W., Świtalski J., Karauda D., Mularczyk-Tomczewska P., et al. The Influence of Vaginal HPV Self-Sampling on the Efficacy of Populational Screening for Cervical Cancer—An Umbrella Review. Cancers. 2022;14(23):5913 doi:10.3390/cancers14235913.

177. Arbyn M., Verdoodt F., Snijders P.J., Verhoef V.M., Suonio E., Dillner L., et al. Accuracy of human papillomavirus testing on self-collected versus clinician-collected samples: a meta-analysis. Lancet Oncol. 2014;15(2):172-83 doi:10.1016/s1470-2045(13)70570-9.

178. Sy F., Greuel M., Winkler V., Bussmann H., Barnighausen T., Deckert A. Accuracy of HPV testing on self-collected and clinician-collected samples for different screening strategies in African settings: A systematic review and meta-analysis. Gynecol Oncol. 2022;166(2):358-68 doi:<https://dx.doi.org/10.1016/j.ygyno.2022.06.012>.

179. Zhao F.H., Lewkowitz A.K., Chen F., Lin M.J., Hu S.Y., Zhang X., et al. Pooled analysis of a self-sampling HPV DNA Test as a cervical cancer primary screening method. J Natl Cancer Inst. 2012;104(3):178-88 doi:<https://dx.doi.org/10.1093/jnci/djr532>.

180. Malone C., Barnabas R.V., Buist D.S.M., Tiro J.A., Winer R.L. Cost-effectiveness studies of HPV self-sampling: A systematic review. Preventive Medicine. 2020;132:N.PAG-N.PAG doi:10.1016/j.ypmed.2019.105953.

181. Mezei A.K., Armstrong H.L., Pedersen H.N., Campos N.G., Mitchell S.M., Sekikubo M., et al. Cost-effectiveness of cervical cancer screening methods in low- and middle-income countries: A systematic review. Int J Cancer. 2017;141(3):437-46 doi:<https://dx.doi.org/10.1002/ijc.30695>.

182. Serrano B., Ibáñez R., Robles C., Peremiquel-Trillas P., de Sanjosé S., Bruni L. Worldwide use of HPV self-sampling for cervical cancer screening. Prev Med. 2022;154:106900 doi:10.1016/j.ypmed.2021.106900.

183. Tesfahunei H.A., Ghebreyesus M.S., Assefa D.G., Zeleke E.D., Acam J., Joseph M., et al. Human papillomavirus self-sampling versus standard clinician-sampling for cervical cancer screening in sub-Saharan Africa: a systematic review and meta-analysis of randomized controlled trials. Infectious Agents & Cancer. 2021;16(1):1-12 doi:10.1186/s13027-021-00380-5.

184. Yeh P.T., Kennedy C.E., de Vuyst H., Narasimhan M. Self-sampling for human papillomavirus (HPV) testing: a systematic review and meta-analysis. BMJ glob. 2019;4(3):e001351 doi:<https://dx.doi.org/10.1136/bmjgh-2018-001351>.

185. Mekuria S.F., Timmermans S., Borgfeldt C., Jerkeman M., Johansson P., Linde D.S. HPV self-sampling versus healthcare provider collection on the effect of cervical cancer screening uptake and costs in LMIC: a systematic review and meta-analysis. Syst Rev. 2023;12(1):13 doi:10.1186/s13643-023-02252-y.

186. World Health O. Consolidated guidelines on HIV, viral hepatitis and STI prevention, diagnosis, treatment and care for key populations Geneva: World Health Organization; 2022 [cited 2024 23 May]. Available from: <https://apps.who.int/iris/handle/10665/360601>.

187. World Health O. WHO guideline for screening and treatment of cervical pre-cancer lesions for cervical cancer prevention Geneva: World Health Organization; 2021 [cited 2024 23 May]. Available from: <https://apps.who.int/iris/handle/10665/342365>.

188. World Health O. WHO guideline on self-care interventions for health and well-being Geneva: World Health Organization; 2022 [cited 2024 23 May]. Available from: <https://apps.who.int/iris/handle/10665/357828>.

189. World Health O. WHO guideline for screening and treatment of cervical pre-cancer lesions for cervical cancer prevention, second edition: use of mRNA tests for human papillomavirus (HPV) Geneva: World Health Organization; 2021 [cited 2024 23 May]. Available from: <https://apps.who.int/iris/handle/10665/350652>.

190. World Health O. WHO consolidated guideline on self-care interventions for health: sexual and reproductive health and rights Geneva: World Health Organization; 2019 [cited 2024 23 May]. Available from: <https://apps.who.int/iris/handle/10665/325480>.

191. World Health O. Comprehensive cervical cancer control: a guide to essential practice. Geneva: World Health Organization; 2014.
