# Supplementary material for: Human papillomavirus self-sampling versus provider-sampling in low- and middle-income countries: a scoping review of accuracy, acceptability, cost, uptake, and equity
Source: Front Public Health. 2024 Nov 29;12:1439164. doi: 10.3389/fpubh.2024.1439164 (PMC11638174; doi:10.3389/fpubh.2024.1439164)
Supplement: Supplementary file 6 [file Table_6.docx]

# **Annex 6: General Characteristics of Included Primary Studies**

| Author, Year  (Design) | **Population**  **(Sample size; n)** | - **Country** - **Area** - **Income bracket** - **WHO region** - **UN sub-region** - **UN intermediate region** | - **Health setting** - **Form of facility ownership** - **Facility type** - **Non-facility setting** | **Self-sampling**   - - **Assay test**   - **Sample type**   - **Transport medium**   - **Collection device type**   - **Collection device material**   - **Test manufacturer** | **Provider-sampling**   - **Assay test** - **Sample type** - **Transport medium** - **Collection device type** - **Collection device material** - **Test manufacturer** |
| --- | --- | --- | --- | --- | --- |
| Acceptability | | | | | |
| Afzal 2020 ([1](#_ENREF_1))  (Cross-sectional) | Women who presented for cervical cancer screening  (n= 120) | - Liberia - NR - LIC - African Region - Sub-Saharan Africa - Western Africa | - Healthcare facility - Government-owned - Health Centre - NR | - HPV DNA - NR - NR - Swab - NR - Qiagen, Gaithersburg, Maryland, USA | - NR - NR - NR - NR - NR - NR |
| Ahmad 2021 ([2](#_ENREF_2))  (Cross-sectional) | Women in the reproductive age group  (n= 220) | - Malaysia - NR - UMIC - Western Pacific Region - South-Eastern Asia - Unclassified | - Healthcare facility - Government-owned - Hospital - NR | - HPV DNA - NR - NR - NR - NR - NR | - NR - NR - NR - NR - NR - NR |
| Allende 2019 ([3](#_ENREF_3))  (Cross-sectional) | Women from the urban (Central neighbourhood, n = 74), the peri-urban (surrounding area, n = 63) and the rural (Chapare, n = 84) areas, with a range of age between 25 to 64 years  (n= 222) | - Bolivia - Urban; Peri-urban; Rural - LMIC - Region of the Americas - Latin America and the Caribbean - South America | - Community-based - Not applicable - Not applicable - Community health posts | - HPV DNA - Cervicovaginal - Dry - Swab - Cotton - Digene Corporation, Gaithersburg, MD, USA | - NR - NR - NR - Speculum & Brush - NR - NR |
| Allen-Leigh 2017 ([4](#_ENREF_4))  (Qualitative) | Women 20 years and older from three ethnic groups indigenous to Mexico in three different states: Mam women in Chiapas, Nahuatl-speaking women in Puebla and Huichol women in Jalisco  (n= NR) | - Mexico - Rural - UMIC - Region of the Americas - Latin America and the Caribbean - Central America | - Healthcare facility; Community-based - NR - Health Centre - Community health posts | - HPV DNA - NR - NR - NR - NR - NR | - NR - NR - NR - NR - NR - NR |
| Arrossi 2016  ([5](#_ENREF_5)) (Cluster randomized controlled trial) | Women present at home during their routine visit and instructed them about cervical cancer and HPV testing and offered them the self-collection option  (n= 3049) | - Argentina - NR - UMIC - Region of the Americas - Latin America and the Caribbean - South America | - Home-based - Not applicable - Not applicable - Home | - HPV DNA - NR - NR - NR - NR - NR | - NR - NR - NR - NR - NR - NR |
| Awua 2017  ([6](#_ENREF_6)) (Cross-sectional) | Healthy women (self-report) between the ages of 15 and 65 years who were willing to provide cervical specimens by either self-collection or health-personnel specimen collection or both were eligible to participate in this study  (n= 228) | - Ghana - NR - LMIC - African Region - Sub-Saharan Africa - Western Africa | - Healthcare facility; Community-based - Government-owned (public) - Hospital - Community health posts | - HPV DNA - Cervical - NR - Brush - Polyethylene - Rovers Medical Devices, Netherlands | - NR - Cervical - NR - Speculum - NR - NR |
| Bakiewicz 2020  ([7](#_ENREF_7)) (Qualitative) | Women aged 25 to 60 years  (n= 21) | - Tanzania - NR - LMIC - African Region - Sub-Saharan Africa - Eastern Africa | - Healthcare facility - Government-owned (public) - Hospital - NR | - HPV DNA - NR - NR - Swab - Cotton - Qiagen, Gaithersburg, Maryland, USA | - NR - NR - NR - NR - NR - NR |
| Berner 2013 ([8](#_ENREF_8))  (Cross-sectional) | We included 243 non-pregnant women aged between 24 and 65 who did not undergo a former hysterectomy from the urban area.  (n= 217) | - Cameroon - Urban - LMIC - African Region - Sub-Saharan Africa - Middle Africa | - Healthcare facility - Government-owned (public) - Hospital - NR | - HPV DNA - Cervical - NR - Swab - Nylon - NR | - Cytology - Cervical - NR - Brush - NR - NR |
| Brandt 2019  ([9](#_ENREF_9))  (Qualitative) | All sexually active women between the ages of 20 and 65 years, permanently living in Dabat Town who, were capable of understanding the study procedures and were willing to participate voluntarily.  (n= 41) | - Ethiopia - Rural - LIC - African Region - Sub-Saharan Africa - Eastern Africa | - Healthcare facility - Private-based - Health Centre - NR | - HPV DNA - NR - NR - Brush - Polyethylene - Rovers Medical Devices, Netherlands | - NR - NR - NR - NR - NR - NR |
| Broquet 2015  ([10](#_ENREF_10)) (Cross-sectional) | Women aged between 30 and 65 years  (n= 300) | - Madagascar - Urban; Rural - LIC - African Region - Sub-Saharan Africa - Eastern Africa | - Healthcare facility - NR - Health Centre - NR | - HPV DNA - NR - NR - Swab - Nylon - ESwab,  Copan, Brescia, Italy | - NR - NR - NR - NR - NR - NR |
| Castle 2019 ([11](#_ENREF_11))  (Cross-sectional) | Women between the ages of 25 and 64 years were recruited because this was the recommended screening age by the Brazilian Cancer Institut  (n= 483) | - Brazil - NR - UMIC - Region of the Americas - Latin America and the Caribbean - South America | - Healthcare facility - Government-owned (public) - Health Centre - NR | - HPV DNA - NR - Dry - Brush - NR - Qiagen, Gaithersburg, Maryland, USA | - NR - NR - NR - NR - NR - NR |
| Dareng 2015  ([12](#_ENREF_12)) (Cross-sectional) | Over 18 years with no obvious physical ailments  (n= 581) | - Nigeria - NR - LMIC - African Region - Sub-Saharan Africa - Western Africa | - Community based - Not applicable - Not applicable - Community health posts | - HPV DNA - NR - NR - NR - NR - NR | - NR - NR - NR - NR - NR - NR |
| Dzuba 2002  ([13](#_ENREF_13))(Cross-sectional) | Women aged 20 and above, use of IMSS services in Morelos, and previous registration in the parent study  (n= 1069) | - Mexico - NR - UMIC - Region of the Americas - Latin America and the Caribbean - Central America | - Healthcare facility - NR - Hospital - NR | - HPV DNA - NR - NR - NR - NR - NR | - NR - NR - NR - NR - NR - NR |
| Eche 2022  ([14](#_ENREF_14)) (Cross-sectional) | Only willing female students within the specified age bracket (18-65 years) and in the College of Humanities were included  (n= 386) | - South Africa - NR - UMIC - African Region - Sub-Saharan Africa - Southern Africa | - Home-based - Not applicable - Not applicable - Home | - HPV DNA - NR - NR - NR - NR - NR | - NR - NR - NR - NR - NR - NR |
| Esber 2017  ([15](#_ENREF_15)) (Cohort) | Women aged 19 to 39 years  (n= 824) | - Malawi - NR - LIC - African Region - Sub-Saharan Africa - Eastern Africa | - Community-based - Not applicable - Not applicable - Community health posts | - HPV DNA - Vaginal - NR - Swab - NR - NR | - NR - NR - NR - NR - NR - NR |
| Flores 2003  ([16](#_ENREF_16)) (Cross-sectional) | Women between the ages of 20 and 80 without a prior diagnosis of CIN 2/3 or CC, hysterectomy or other treatment.  (n= 7732) | - Mexico - NR - UMIC - Region of the Americas - Latin America and the Caribbean - Central America | - Healthcare facility - Private-based - Health Centre - NR | - HPV DNA - NR - NR - NR - NR - NR | - NR - NR - NR - NR - NR - NR |
| Goldstein 2020  ([17](#_ENREF_17)) (Cross-sectional) | Chinese women aged 35 to 65 years  (n= 600) | - China - Rural - UMIC - Western Pacific Region - Eastern Asia - Unclassified | - Healthcare facility - Government-owned (public) - Health Centre - NR | - HPV DNA - Vaginal - NR - Brush - NR - NR | - NR - NR - NR - NR - NR - NR |
| Gottschlich 2017  ([18](#_ENREF_18)) (Cross-sectional) | Women aged 25 to 54 were eligible to provide a self-collected sample for HPV testing. Menstruating and pregnant women were also excluded from self-collection.  (n= 202) | - Guatemala - Urban; Rural - UMIC - Region of the Americas - Latin America and the Caribbean - Central America | - Community-based - Not applicable - Not applicable - Community health posts | - HPV DNA - Vaginal - NR - Brush - NR - NR | - NR - NR - NR - NR - NR - NR |
| Gottschlich 2019 ([19](#_ENREF_19)) (Cross-sectional study) | Women attending clinic visit, between ages 25 and 60 years  (n= 267) | - Thailand - NR - UMIC - South-East Asia Region - South-Eastern Asia - Unclassified | - Healthcare facility - Government-owned (public) - Health Centre - NR | - HPV DNA - NR - NR - NR - NR - NR | - NR - NR - NR - NR - NR - NR |
| Guan 2012  ([20](#_ENREF_20)) (Cross-sectional) | Women who were not pregnant and had not had a hysterectomy  (n= 174) | - China - NR - UMIC - Western Pacific Region - Eastern Asia - Unclassified | - Healthcare facility - NR - Hospital - NR | - HPV DNA - Cervical - NR - Brush - NR - Qiagen, Gaithersburg, Maryland, USA | - HPV DNA - Cervical - NR - Brush - NR - Qiagen, Gaithersburg, Maryland, USA |
| He 2020  ([21](#_ENREF_21)) (Cross-sectional) | Women able to read and understand the questionnaire, either on their own or with the help of staff  (n= 810) | - China - NR - UMIC - Western Pacific Region - Eastern Asia - Unclassified | - Healthcare facility - NR - Hospital - NR | - HPV DNA - NR - NR - NR - NR - NR | - NR - NR - NR - NR - NR - NR |
| Hood 2020  ([22](#_ENREF_22)) (Cohort) | Participants for UTHA from the catchment area of a community hospital using stratified cluster sampling.13 The hospital serves 68 villages with a combined population of about 20,000 residents  (n= 1043) | - Malawi - Rural - LIC - African Region - Sub-Saharan Africa - Eastern Africa | - Community-based - Not applicable - Not applicable - Community health posts | - HPV DNA - Vaginal - Wet - swab - Cotton - NR | - NR - NR - NR - NR - NR - NR |
| Huchko 2018  ([23](#_ENREF_23)) (Cluster randomized controlled trial) | The study population comprised women aged 25-65 years living in Migori County who had an intact uterus and cervix  (n= 2898) | - Kenya - NR - LMIC - African Region - Sub-Saharan Africa - Eastern Africa | - Community-based - Not applicable - Not applicable - Community health posts | - HPV DNA - NR - NR - Brush - NR - Qiagen, Gaithersburg, Maryland, USA | - NR - NR - NR - NR - NR - NR |
| Khoo 2021  ([24](#_ENREF_24)) (Cross-sectional) | Women aged 35 to 45 who consented to vaginal self-sampling HPV testing and answering questionnaires were recruited to this study  (n= 725) | - Malaysia - NR - UMIC - Western Pacific Region - South-Eastern Asia - Unclassified | - Community-based - Not applicable - Not applicable - Community health posts | - HPV DNA - Vaginal - Dry - Swab - NR - NR | - NR - NR - NR - NR - NR - NR |
| Kohler 2019 ([25](#_ENREF_25))  (Cross-sectional) | WLHIV aged 25 years and older attending the IDCC for routine healthcare appointments  (n= 104) | - Botswana - NR - UMIC - African Region - Sub-Saharan Africa - Southern Africa | - Healthcare facility - Government-owned (public) - Health Centre - NR | - HPV DNA - Vaginal - Wet - Swab - Nylon - NR | - NR - NR - NR - NR - NR - NR |
| Laskow 2017  ([26](#_ENREF_26)) (Cross-sectional) | Eligible participants were aged 30-59 years, not pregnant, and capable of providing informed consent. Women screened within the past two years or with a history of cryotherapy, loop electrosurgical excision procedure, or hysterectomy were excluded from the study  (n= 60) | - El Salvador - NR - UMIC - Region of the Americas - Latin America and the Caribbean - Central America | - Community-based - Not applicable - Not applicable - Community health posts | - Hybrid Capture II HPV DNA - Vaginal - NR - Brush - NR - Digene Corporation, Gaithersburg, MD, USA | - NR - NR - NR - NR - NR - NR |
| Li 2022  ([27](#_ENREF_27)) (Cohort) | 1) 30 to 59 years of age, (2) sexually exposed, (3) non-pregnant, and (4) consent for participation  (n= 20103) | - China - Rural - UMIC - Western Pacific Region - Eastern Asia - Unclassified | - Healthcare facility - NR - Hospital - NR | - HPV DNA - Vaginal - Wet - Brush - NR - GI-Shenzhen, China | - NR - NR - NR - NR - NR - NR |
| Mahande 2021  ([28](#_ENREF_28)) (Cross-sectional) | Women between the ages of 25 and 55 years, the high-risk age group for cervical cancer.  (n= 350) | - Tanzania - Peri-urban - LMIC - African Region - Sub-Saharan Africa - Eastern Africa | - Healthcare facility; Community-based - Government-owned (public) - Health Centre - Community health posts | - HPV DNA - Vaginal - NR - Swab - NR - Qiagen, Gaithersburg, Maryland, USA | - NR - NR - NR - NR - NR - NR |
| Mahomed 2014  ([29](#_ENREF_29)) (Cross-sectional) | WLHIV, willing to participate in the study, provided written informed consent, were English speaking, between 20 and 65 years of age and were not pregnant  (n= 106) | - South Africa - Urban; Rural - UMIC - African Region - Sub-Saharan Africa - Southern Africa | - Healthcare facility - Government-owned - Health Centre - NR | - HPV DNA - Vaginal - NR - brush - NR - NR | - NR - Vaginal - Wet - Lavager - NR - NR |
| Mandigo 2015  ([30](#_ENREF_30)) (Cross-sectional) | Premenopausal women aged 30–50 years who had not undergone a cervical smear in the previous 3 years and had no history of cervical cancer, hysterectomy, or cervical incompetence  (n= 439) | - Haiti - Rural - LMIC - Region of the Americas - Latin America and the Caribbean - Caribbean | - Community-based - Not applicable - Not applicable - Community health posts | - Hybrid Capture II HPV DNA - NR - wet - NR - NR - Preventive Oncology International (Cleveland Heights, OH, USA)' | - NR - NR - NR - NR - NR - NR |
| Manguro 2018  ([31](#_ENREF_31)) (Cross-sectional) | Women are invited into this parent cohort if they are 18 to 45 years old, reside in Mombasa area, are self-identifying as exchanging sex for payment in cash or in kind and are able to provide informed consent  (n= 199) | - Kenya - Urban - LMIC - African Region - Sub-Saharan Africa - Eastern Africa | - Healthcare facility - Government-owned (public) - Health Centre - NR | - HPV DNA - cervicovaginal - Wet - brush - NR - Rovers Medical Devices | - HPV DNA - Cervical - wet - speculum - NR - Hologic, USA |
| Maza 2018  ([32](#_ENREF_32)) (Cross-sectional) | Women who; 1) aged 30 to 59 years, 2) had not undergone cytology screening within the last three years, HPV screening within the last five years, or had never been screened, and 3) had not undergone procedures associated with treatment of CIN related to cervical cancer prevention (e.g., cryotherapy, cold knife conisation, or hysterectomy) or did not have a history of cervical cancer'  (n= 1989) | - El Salvador - Rural - UMIC - Region of the Americas - Latin America and the Caribbean - Central America | - Community-based - Not applicable - Not applicable - Community health posts | - Care HPV DNA - NR - NR - NR - NR - NR | - NR - NR - NR - NR - NR - NR |
| Maza 2020 ([33](#_ENREF_33))  (Cross-sectional) | Transgender men who were female sex assigned at birth, (2) age 18 years or older, (3) agreed to participate in the study, (4) were willing and able to provide informed consent.  (n= 24) | - El Salvador - NR - LMIC - Region of the Americas - Latin America and the Caribbean - Central America | - Healthcare facility - Private non-propriety (faith-based/not-for-profit); Other: Advocacy group - Health Centre - NR | - Care HPV DNA - NR - NR - Brush - NR - Qiagen, Gaithersburg, VA | - NR - NR - NR - NR - NR - NR |
| McFarlane 2021 ([34](#_ENREF_34))  (Qualitative) | The inclusion is not clearly reported- this study mentions that Jamaican women were engaged as focus group participants.  (n= 36) | - Jamaica - NR - UMIC - Region of the Americas - Latin America and the Caribbean - Caribbean | - Community-based - Not applicable - Not applicable - Community health posts | - HPV DNA - NR - wet - Swab - NR - NR | - NR - NR - NR - NR - NR - NR |
| Megersa 2020  ([35](#_ENREF_35))(Qualitative) | Women who had participated in a community-wide home-based HPV self-sampling, chws, wdals, and the sample collectors of the pilot study  (n= 47) | - Ethiopia - Rural - LIC - African Region - Sub-Saharan Africa - Eastern Africa | - Community-based - Not applicable - Not applicable - Community health posts | - HPV DNA - Vaginal - NR - brush - NR - NR | - NR - NR - NR - NR - NR - NR |
| Mitchell 2011  ([36](#_ENREF_36))(Cross-sectional) | 30- to 65-year-old women who lived and/or worked in Kisenyi  (n= 300) | - Uganda - NR - LIC - African Region - Sub-Saharan Africa - Eastern Africa | - Community-based - Not applicable - Not applicable - Community health posts | - HPV DNA - NR - NR - NR - NR - NR | - NR - NR - NR - NR - NR - NR |
| Mitchell 2017  ([37](#_ENREF_37))(Cross-sectional) | WLHIV aged 30-69 years old attending a routine appointment for their HIV care and access to a mobile telephone  (n= 84) | - Uganda - NR - LIC - African Region - Sub-Saharan Africa - Eastern Africa | - Healthcare facility - Government-owned (public) - Health Centre - NR | - HPV DNA - Vaginal - NR - swab - NR - NR | - NR - NR - NR - NR - NR - NR |
| Mremi 2021  ([38](#_ENREF_38))(Cross-sectional) | Women aged 25-60 years who were permanent residents in the identified districts  (n= 1108) | - Tanzania - Rural - LMIC - African Region - Sub-Saharan Africa - Eastern Africa | - Healthcare facility - Government-owned (public); Private non-propriety (faith-based/not-for-profit) - Hospital - NR | - Care HPV DNA - Vaginal - NR - Brush - NR - Rovers Medical Devices | - NR - NR - NR - NR - NR - NR |
| Murchland 2019  ([39](#_ENREF_39)) (Cross-sectional) | Selected households that had at least one woman available between the ages of 18 and 60 were invited to participate in the survey.  (n= 956) | - Guatemala - Rural - UMIC - Region of the Americas - Latin America and the Caribbean - Central America | - Community-based - Not applicable - Not applicable - Community health posts | - Hybribio HR-13 kit HPV DNA - NR - wet - Swab - NR - NR | - NR - NR - NR - NR - NR - NR |
| Nyabigambo 2023 ([40](#_ENREF_40))  (Qualitative) | We interviewed 24 WLWH, all aged 25 to 49 years  (n= 30) | - Uganda - Rural - LIC - African Region - Sub-Saharan Africa - Eastern Africa | - Healthcare facility; Community-based; Home-based - NR - Health Centre - Home | - HPV DNA - NR - NR - Swab - NR - Approvix, Sweden | - HPV DNA - NR - NR - Brush - NR - NR |
| Oberlin 2018  ([41](#_ENREF_41)) (Cross-sectional) | Women aged 18 years or older  (n= 300) | - South Africa - Urban - UMIC - African Region - Sub-Saharan Africa - Southern Africa | - Healthcare facility - Government-owned - Hospital; Health Centre - NR | - HPV DNA - NR - NR - Swab - NR - NR | - NR - NR - NR - Swab - NR - NR |
| Oketch 2019 ([42](#_ENREF_42))  (Cluster randomized controlled trial) | Women aged 25 to 65 years living within selected communities within Migori County and offered HPV-based self-screening in health facilities or through community health campaigns  (n= 120) | - Kenya - Rural - LMIC - African Region - Sub-Saharan Africa - Eastern Africa | - Community-based - Not applicable - Not applicable - Community health posts | - HPV DNA - NR - NR - NR - NR - Qiagen, Gaithersburg, MD | - NR - NR - NR - NR - NR - NR |
| Oneko 2022  ([43](#_ENREF_43)) (Cross-sectional) | Women aged 18 to 55 years who were receiving care in outpatient clinics of selected facilities in Meru District  (n= 706) | - Tanzania - Urban; Rural - LMIC - African Region - Sub-Saharan Africa - Eastern Africa | - Healthcare facility - Government-owned - Health Centre - NR | - HPV DNA - NR - NR - NR - NR - NR | - NR - NR - NR - NR - NR - NR |
| Oranratanaphan 2014  ([44](#_ENREF_44)) (Cross-sectional) | Women aged 30 to 65 years with intact cervix were included  (n= 100) | - Thailand - NR - UMIC - South-East Asia Region - South-Eastern Asia - Unclassified | - Healthcare facility - Government-owned (public) - Hospital - NR | - HPV DNA - Vaginal - Dry - Brush - NR - NR | - Cytology - Cervical - NR - NR - NR - NR |
| Peedicayil 2014  ([45](#_ENREF_45)) (Cross-sectional) | Married or previously married women  (n= 809) | - India - Rural - LMIC - South-East Asia Region - Southern Asia - Unclassified | - Healthcare facility; Community-based - NR - Health Centre - Community health posts | - HPV DNA - Vaginal - NR - Swab - NR - NR | - HPV DNA - Cervical - NR - Swab - NR - NR |
| Phoolcharoen 2018  ([46](#_ENREF_46)) (Cross-sectional) | Women aged 30 to 70 years who visited the colposcopy clinic at Chulabhorn Hospital, Bangkok  (n= 248) | - Thailand - NR - UMIC - South-East Asia Region - South-Eastern Asia - Unclassified | - Healthcare facility - Private non-propriety (faith-based/not-for-profit) - Hospital - NR | - HPV DNA - Vaginal - dry - Brush - NR - Rovers Medical Devices B.V., Oss, Netherlands | - HPV DNA - cervical - NR - Brush - NR - Rovers Medical |
| Possati-Resende 2020  ([47](#_ENREF_47)) (Cohort) | Rural dwellers (women) in the Barretos region who were in their homes at the time of the police officer's visit  (n= 386) | - Brazil - Rural - UMIC - Region of the Americas - Latin America and the Caribbean - South America | - Healthcare facility; Community-based - Government-owned (public) - Hospital - Community health posts | - Cobas 4800 HPV DNA - Vaginal - Wet - Brush - NR - Rovers Medical Devices, The Netherlands | - NR - NR - NR - NR - NR - Rovers Medical Devices, Netherlands |
| Qu 2023  ([48](#_ENREF_48)) (Cross-sectional) | Women who could read and understand the questionnaire, over 25 years of age  (n= 862) | - China - NR - UMIC - Western Pacific Region - Eastern Asia - Unclassified | - NR - NR - NR - NR | - HPV DNA - NR - NR - NR - NR - NR | - NR - NR - NR - NR - NR - NR |
| Quincy 2012  ([49](#_ENREF_49)) (Cross-sectional) | Non-pregnant, 25 to 60-year-old women with intact uteri from Leon, Nicaragua.  (n= 245) | - Nicaragua - NR - LMIC - Region of the Americas - Latin America and the Caribbean - Central America | - Healthcare facility - Government-owned (public) - Hospital - NR | - HPV DNA - Vaginal - NR - swab - NR - NR | - NR - Cervical - NR - brush - NR - NR |
| Rawat 2021 ([50](#_ENREF_50))  (Mixed methods) | Women age 18 years and above providing written informed consent. Community women must live within the catchment populations of the health centres, and hcps must provide services to women or children at the health centre  (n= 64) | - Uganda - Rural - LIC - African Region - Sub-Saharan Africa - Eastern Africa | - Healthcare facility - Government-owned - Health Centre - NR | - HPV DNA - Vaginal - NR - Swab - NR - NR | - NR - NR - NR - NR - NR - NR |
| Rodrigues 2018  ([51](#_ENREF_51)) (Cross-sectional) | Non-indigenous HIV-infected and HIV-uninfected women living in the Tapajos region  (n= 153) | - Brazil - NR - UMIC - Region of the Americas - Latin America and the Caribbean - South America | - Healthcare facility - Government-owned - Health Centre - NR | - HPV DNA - NR - NR - NR - NR - NR | - HPV DNA - Cervical - Wet - Brush - NR - NR |
| Rosenbaum 2014  ([52](#_ENREF_52)) (Cross-sectional) | Women aged 30 to 49 years, not pregnant, able to provide informed consent, and without history of cryotherapy, loop electrosurgical excision procedure, or hysterectomy.  (n= 518) | - El Salvador - Rural - UMIC - Region of the Americas - Latin America and the Caribbean - Central America | - Healthcare facility; Community-based - Government-owned (public) - Health Centre - Community health posts | - Care HPV DNA - Cervicovaginal - NR - NR - NR - NR | - Care HPV DNA - Cervical - NR - Speculum exam - NR - NR |
| Rositch 2012  ([53](#_ENREF_53)) (Cohort) | HIV discordant couples reporting sex acts in the previous 3 months, planned to stay in Nairobi in their current relationship for at least 2 years, and if one member of the couple was HIV-1-infected and the other HIV-1 susceptible  (n= 409) | - Kenya - NR - LMIC - African Region - Sub-Saharan Africa - Eastern Africa | - Healthcare facility - Government-owned - Health Centre - NR | - HPV DNA - Vaginal - NR - NR - NR - NR | - HPV DNA - Cervical - NR - NR - NR - NR |
| Saidu 2019  ([54](#_ENREF_54))  (Mixed methods) | Women aged 30 to 65 years were recruited from a referral colposcopy clinic  (n= 863) | - South Africa - NR - UMIC - African Region - Sub-Saharan Africa - Southern Africa | - Healthcare facility; Home-based - Government-owned (public) - Health Centre - Home | - HPV DNA - Vaginal - Wet - Swab - Dacron - NR | - Colposcopy - Cervical - NR - colposcope - NR - NR |
| Sormani 2022  ([55](#_ENREF_55)) (Cross-sectional) | All women aged 30 to 49 years were included in the study after full understanding of the procedure, and an informed consent form, available in French and in English, was signed  (n= 2201) | - Cameroon - Rural - LMIC - African Region - Sub-Saharan Africa - Middle Africa | - Healthcare facility - Government-owned (public) - Hospital - NR | - HPV DNA - NR - NR - NR - NR - NR | - NR - NR - NR - NR - NR - NR |
| Vallely 2022  ([56](#_ENREF_56)) (Non-randomized crossover trial) | Women aged 30 to 59 years attending cervical screening services at the two clinical sites, willing to comply with study procedures, and able to provide written informed consent  (n= 4285) | - Papua New Guinea - NR - LMIC - Western Pacific Region - Melanesia - Unclassified | - Healthcare facility - Government-owned (public) - Hospital - NR | - GeneXpert HPV DNA - Vaginal - Wet - Brush - NR - NR | - NR - NR - NR - NR - NR - NR |
| Varun 2023  ([57](#_ENREF_57)) (Cross-sectional) | 18 years of age and over, spoke English or Kinyarwanda and were able to provide informed consent  (n= 374) | - Rwanda - Urban; Rural - LIC - African Region - Sub-Saharan Africa - Eastern Africa | - Healthcare facility - Government-owned (public) - Hospital - NR | - HPV DNA - NR - NR - NR - NR - NR | - NR - NR - NR - NR - NR - NR |
| Vega Crespo 2022  ([58](#_ENREF_58)) (Cross-sectional) | Women aged between 18 and 70 years old; not having undergone an excision or destructive treatment of cervical intraepithelial neoplasia; not having used vaginal medication at least a week before the examination; not having had sexual intercourse for at least 48 h previous to the examination; not being pregnant; and the absence of menstrual bleeding at the examination  (n= 120) | - Ecuador - Rural - UMIC - Region of the Americas - Latin America and the Caribbean - South America | - NR - NR - NR - NR | - HPV DNA - Vaginal - NR - Brush - NR - NR | - HPV DNA - Cervical - Wet - Brush - NR - NR |
| Yoshida 2013  ([59](#_ENREF_59)) (Cross-sectional) | Women who a) worked in Khammouane Provincial Hospital, b) worked in Xebangfay District Health Office, and c) lived in Tung village in Sibounhouane subdistrict  (n= 290) | - Lao PDR - NR - LMIC - Western Pacific Region - South-Eastern Asia - Unclassified | - Healthcare facility - Government-owned - Hospital - NR | - HPV DNA - Vaginal - Wet - Brush - NR - NR | - NR - NR - NR - NR - NR - NR |
| Acceptability & Accuracy | | | | | |
| Adamson 2015 ([60](#_ENREF_60))  (Cross-sectional) | WLHIV, 25 years or older, who did not have a cervical cytology test result documented in their chart within the past three years  (n= 325) | - South Africa - Urban - UMIC - African Region - Sub-Saharan Africa - Southern Africa | - Healthcare facility - Government-owned (public) - Health Centre - NR | - Aptima HPV mRNA - Vaginal - Wet - Tampon - NR - NR | - Aptima HPV mRNA - Cervical - Wet - Brush - NR - NR |
| Anand 2022 ([61](#_ENREF_61))  (Cross-sectional) | Sexually active non-pregnant women in the age group of 30 to 55 years with no previous history of cervical cancer  (n= 485) | - India - Urban; Rural - LMIC - South-East Asia Region - Southern Asia - Unclassified | - Home-based - Not applicable - Not applicable - Home | - HPV DNA - NR - NR - NR - NR - NR | - NR - NR - NR - NR - NR - NR |
| de Melo Kuil 2017  ([62](#_ENREF_62)) (Cross-sectional) | Women aged 18-64 years who were referred to the Colposcopy Ambulatory of the Prevention Department at Barretos Cancer Hospital (Brazil) due to abnormal (atypical squamous cells of uncertain significance or worse) cervical cytology test results (Pap smear)  (n= 171) | - Brazil - NR - UMIC - Region of the Americas - Latin America and the Caribbean - South America | - Healthcare facility - Government-owned - Hospital - NR | - HPV DNA - Vaginal - Wet - NR - NR - NR | - NR - Cervical - Wet - Brush - NR - NR |
| Eamratsameekool 2023  ([63](#_ENREF_63)) (Cross-sectional) | Women aged 30 to 59 years  (n= 535) | - Thailand - NR - UMIC - South-East Asia Region - South-Eastern Asia - Unclassified | - Healthcare facility - NR - Hospital - NR | - Cobas 4800 HPV DNA - Cervical - Wet - Swab - Cotton - Digene Corporation, Gaithersburg, MD, USA | - Cobas 4800 HPV DNA - Cervical - Wet - Brush - NR - NR |
| Esber 2018  ([64](#_ENREF_64)) (Cross-sectional) | Women 18 to 49 years of age, spoke Chichewa, had at least one genitourinary symptom, consented to be examined and give biological specimens for testing, and resided in Lilongwe District  (n= NR) | - Malawi - Rural - LIC - African Region - Sub-Saharan Africa - Eastern Africa | - Healthcare facility - Government-owned - Health Centre - NR | - GeneXpert HPV DNA - Vaginal - Wet - Swab - Cotton - Digene Corporation, Gaithersburg, MD, USA | - GeneXpert HPV DNA - Cervical - Wet - Brush - NR - NR |
| Flores 2021  ([65](#_ENREF_65)) (Cross-sectional) | Sexually active women aged 30 to 65 with no history of medical or surgical treatment (radiotherapy, chemotherapy, hysterectomy, cone biopsy) for cervical cancer.  (n= 505) | - Mexico; Peru - NR - UMIC - Region of the Americas - Latin America and the Caribbean - Central America | - Healthcare facility - Government-owned (public) - Health Centre - NR | - Abbott HPV DNA - Cervicovaginal - Wet - NR - NR - NR | - Abbott HPV mRNA - Cervical - Wet - Brush - NR - NR |
| Haile 2019  ([66](#_ENREF_66)) (Cross-sectional) | Women aged 20 years or older, had an intact uterus, had no history of cervical cancer, were mentally competent, and able and willing to provide informed consent  (n= 83) | - Ethiopia - Urban - LIC - African Region - Sub-Saharan Africa - Eastern Africa | - Healthcare facility - Private-based - Hospital - NR | - HPV DNA - Vaginal - Wet - Brush - NR - NR | - HPV DNA - Ectocervical and endocervical - Wet - Brush - NR - NR |
| Islam 2020  ([67](#_ENREF_67)) (Cross-sectional) | Women aged 18 years and above; 2. Residing in the Mombasa area; 3. Self-identifying as exchanging sex for payment in cash or in-kind at the time of enrollment and 4. Able to provide informed consent  (n= 400) | - Kenya - NR - LMIC - African Region - Sub-Saharan Africa - Eastern Africa | - Healthcare facility - Government-owned (public) - Hospital - NR | - Aptima HPV mRNA - Vaginal - Dry & Wet - Brush - Polyethylene - Rovers Medical Devices, Netherlands | - Aptima HPV mRNA - Cervical - Wet - Brush - NR - Rovers Medical Devices, Netherlands |
| Katanga 2021  ([68](#_ENREF_68)) (Cross-sectional) | Women in the age group 25 to 60 years who attended routine cervical cancer screening  (n= 464) | - Tanzania - Urban; Peri-urban - LMIC - African Region - Sub-Saharan Africa - Eastern Africa | - Healthcare facility - Government-owned (public); Private-based - Hospital - NR | - Care HPV DNA - Cervicovaginal - Wet - Brush - Polyethylene - Rovers Medical Devices, Netherlands | - Care HPV DNA - Cervical - Wet - NR - NR - QIAGEN GmbH, Hilden, China |
| Madhivanan 2021  ([69](#_ENREF_69)) (Cross-sectional) | Women 30 years or older, who have not undergone cervical cancer screening within the last three years, and having the capacity to undergo informed consent process  (n= 120) | - India - Rural - LMIC - South-East Asia Region - Southern Asia - Unclassified | - Healthcare facility - NR - Health Centre - NR | - HPV DNA - Vaginal - Wet - swab - NR - Digene Corporation, Gaithersburg, MD, USA | - HPV DNA - Cervical - NR - Brush - NR - NR |
| Obiri-Yeboah 2017  ([70](#_ENREF_70)) (Cross-sectional) | Every fifth woman aged 18 years was systematically selected from the list of daily attendants, starting with a randomly picked attendance number for the first woman  (n= 194) | - Ghana - NR - LMIC - African Region - Sub-Saharan Africa - Western Africa | - Healthcare facility - NR - Hospital - NR | - Care HPV DNA - Vaginal - Wet - Brush - NR - Qiagen, Gaithersburg, MD | - Care HPV DNA - Cervical - Wet - Brush - NR - Qiagen, Gaithersburg, MD |
| Saidu 2021  ([71](#_ENREF_71)) (Cross-sectional) | Women from the general population seeking primary screening and (2) a referral population composed of women referred for colposcopy because of abnormal screening test results.  (n= 705) | - Malawi; South Africa - NR - LIC; UMIC - African Region - Sub-Saharan Africa - Eastern Africa; Southern Africa | - Healthcare facility - Government-owned (public) - Health Centre - NR | - GeneXpert HPV DNA - Vaginal - Wet - Swab - Nylon - NR | - GeneXpert HPV DNA - Cervical - Wet - Brush - Plastic - NR |
| Taku 2020  ([72](#_ENREF_72)) (Cross-sectional) | Women aged over 30 years attending the community health clinic for CC screening or others reasons, age over 18 years with abnormal cervical cytology and CC  (n= 737) | - South Africa - Rural - UMIC - African Region - Sub-Saharan Africa - Southern Africa | - Healthcare facility - Government-owned (public) - Health Centre - NR | - HPV DNA - Vaginal - Wet - Brush - NR - NR | - HPV DNA - Cervical - NR - Brush - NR - NR |
| Tiiti 2021  ([73](#_ENREF_73)) (Cross-sectional) | Women aged 18 years and older  (n= 527) | - South Africa - NR - UMIC - African Region - Sub-Saharan Africa - Southern Africa | - Healthcare facility - Government-owned - Hospital - NR | - Abbott HPV DNA - NR - Wet - Tampon - NR - NR | - Abbott HPV DNA - Cervical - Wet - Brush - NR - NR |
| Van De Wijgert 2006  ([74](#_ENREF_74)) (Mixed methods) | Women 18 years or older, sexually active, not pregnant, and willing and able to comply with the study protocol and give written informed consent  (n= 450) | - South Africa - Peri-urban - UMIC - African Region - Sub-Saharan Africa - Southern Africa | - Healthcare facility - Government-owned (public) - Health Centre - NR | - Hybrid capture II HPV DNA - Vaginal - Wet - Tampon - Cotton - NR | - Digene HC2 High-Risk HPV DNA - Vaginal - Wet - Swab; brush - NR - NR |
| Wong 2018  ([75](#_ENREF_75)) (Cross-sectional) | Women aged 18 years or older, not currently pregnant, had no known abnormal Papanicolaou test results and not having presented symptoms of cervical cancer, genital cancer, cervical surgery, or immune treatment of the cervix during the 6months before recruitment into the study  (n= 68) | - China - NR - UMIC - Western Pacific Region - Eastern Asia - Unclassified | - Healthcare facility - Private non-propriety (faith-based/not-for-profit - Health Centre - NR | - HPV DNA - Vaginal - Wet - Swab - Dacron - NR | - HPV DNA and Pap smear - Cervical - NR - Brush; Swab - Dacron - NR |
| Acceptability & Costing | | | | | |
| Abdullah 2018 ([76](#_ENREF_76))  (Cross-sectional) | Married women, aged 28 to 60 years’ old  (n= 164) | - Malaysia - NR - UMIC - Western Pacific Region - South-Eastern Asia - Unclassified | - Healthcare facility - Government-owned (public) - Health Centre - NR | - Hybrid Capture II HPV DNA - Vaginal - NR - Brush - NR - Qiagen Biotechnology, Venlo, Netherlands | - NR - Vaginal - NR - NR - NR - Qiagen Biotechnology, Venlo, Netherlands |
| Acceptability & Uptake | | | | | |
| Bansil 2014  ([77](#_ENREF_77)) (Cross-sectional) | Female staff members  (n= 3863) | - India; Nicaragua; Uganda - NR - LMIC; LIC; - South-East Asia Region; Region of the Americas; African Region - Southern Asia; Latin America and the Caribbean; Sub-Saharan Africa - Unclassified; Central America; Eastern Africa | - Healthcare facility; Home-based - Government-owned (public) - Health Centre - Home | - Care HPV DNA - Vaginal - NR - Brush - NR - NR | - Care HPV DNA - Cervical - NR - Speculum & Brush - NR - NR |
| Behnke 2020 ([78](#_ENREF_78)) (Mixed-methods)  ([78](#_ENREF_78)) (Cross-sectional) | Women aged between 30 and 65 years, living or working in Karu who do not plan to move out of the community over the next 6 months  (n= 52) | - Ghana - Rural - LMIC - African Region - Sub-Saharan Africa - Western Africa | - Healthcare facility - Private non-propriety (faith-based/not-for-profit - Health Centre - NR | - HPV DNA - NR - NR - NR - NR - NR | - NR - NR - NR - NR - NR - NR |
| Modibbo 2017  ([79](#_ENREF_79)) (Randomized crossover trial) | Only married women, aged 28 to 60 years’ old  (n= 400) | - Nigeria - Peri-urban - LMIC - African Region - Sub-Saharan Africa - Western Africa | - Healthcare facility; Community-based - Government-owned (public) - Hospital - Community health posts | - HPV DNA - Cervicovaginal - Dry - Swab - Nylon - NR | - HPV DNA - Cervicovaginal - Dry - Swab - Nylon - NR |
| Accuracy | | | | | |
| Adedimeji 2020 ([80](#_ENREF_80))  (Cross-sectional) | Women living with HIV attending clinics at the out-patient department of an HIV treatment in Limbe Regional Hospital.  (n= 878) | - Cameroon - NR - LMIC - African Region - Sub-Saharan Africa - Middle Africa | - Healthcare facility - Government-owned (public) - Hospital - NR | - GeneXpert HPV DNA - Vaginal; Cervical - Wet - Brush-Viba - NR - NR | - GeneXpert HPV DNA - Cervical - Wet - Brush-Cervex - NR - NR |
| Awua 2020 ([81](#_ENREF_81)) (Cross-sectional) | Women aged 15 to 65 years of age residing within Akuse community, who were not pregnant at the time of study recruitment.  (n= 253) | - Ghana - NR - LMIC - African Region - Sub-Saharan Africa - Western Africa | - Healthcare facility; Community-based - Government-owned (public) - Hospital - Community health posts | - HPV DNA - Vaginal; Cervical - Wet - Brush-Viba - NR - NR | - HPV DNA - Cervical - Wet - Cytobrush - NR - NR |
| Bansil 2015 ([82](#_ENREF_82)) (Cross-sectional) | Women between 25 and 60 years  (n= 1321) | - Uganda - NR - LIC - African Region - Sub-Saharan Africa - Eastern Africa | - Healthcare facility - Government-owned (public) - Hospital - NR | - Care HPV DNA - Vaginal; Cervical - NR - NR - NR - NR | - HPV-DNA - Cervical - NR - NR - NR - NR |
| Belinson 2003  ([83](#_ENREF_83)) (Cross-sectional) | Women aged 35 to 50 years  (n= 9183) | - China - NR - UMIC - Western Pacific Region - Eastern Asia - Unclassified | - Healthcare facility - Government-owned (public) - Hospital - NR | - Hybrid Capture II HPV DNA - Vaginal - Wet - Brush - NR - Digene Corp. Gaithersburg, MD, USA | - Hybrid Capture II HPV DNA - Endocervical - Wet - Brush - NR - NR |
| Bhatla 2009 ([84](#_ENREF_84))  (Cross-sectional) | Women presenting with complaints of persistent vaginal discharge, irregular menstrual bleeding, post-coital bleeding, or those found on examination to have an unhealthy cervix  (n= 546) | - India - NR - LMIC - South-East Asia Region - Southern Asia - Unclassified | - Healthcare facility - NR - Hospital - NR | - HPV DNA - Cervical - Wet - Brush - NR - Qiagen, Gaithersburg, Maryland, USA | - HPV DNA - Cervical - Wet - Brush - NR - Qiagen, Gaithersburg, Maryland, USA |
| Bogale 2022 ([85](#_ENREF_85))  (Cross-sectional) | All human immunodeficiency virus (HIV) positive women that had antiretroviral therapy (ART) follow-up and age older than 24 years, who volunteered to participate in the study, and signed consent  (n= 497) | - Ethiopia - NR - LIC - African Region - Sub-Saharan Africa - Eastern Africa | - Healthcare facility - Government-owned (public) - Hospital - NR | - Abbott HPV DNA - Cervicovaginal - Wet - Brush - NR - Abbott laboratories, USA | - Abbott HPV DNA - Cervicovaginal - Wet - Speculum - NR - Abbott laboratories, USA |
| Boggan 2015  ([86](#_ENREF_86)) (Cross-sectional) | Women between the ages of 25 and 65 years who had engaged in vaginal intercourse at least once during their lifetimes  (n= 1845) | - Haiti - NR - LMIC - Region of the Americas - Latin America and the Caribbean - Caribbean | - Healthcare facility - NR - Health Centre - NR | - HPV DNA - Vaginal - NR - Brush - Dacron - Qiagen, Gaithersburg, Maryland, USA | - HPV DNA - Cervical - NR - Brush - NR - Qiagen, Gaithersburg, Maryland, USA |
| Chen 2014  ([87](#_ENREF_87)) (Cross-sectional) | Women aged 25 to 65 years  (n= 7500) | - China - Rural - UMIC - Western Pacific Region - Eastern Asia - Unclassified | - Healthcare facility - Government-owned (public) - Hospital - NR | - Hybrid Capture II HPV DNA - Vaginal - NR - NR - NR - NR | - Care HPV DNA - Cervical - Wet - NR - NR - NR |
| Chen 2016 ([88](#_ENREF_88)) (Cross-sectional) | Women aged 18 years and over who visited the gynaecological outpatient clinics at Shanghai Tongji Hospital, China, with both negative cytology and histopathology  (n= 202) | - China - NR - UMIC - Western Pacific Region - Eastern Asia - Unclassified | - Healthcare facility - Government-owned - Hospital - NR | - Abbott HPV DNA - Cervicovaginal - Dry - Brush-Evalyn Brush - NR - NR | - Abott HPV DNA - Cervical - Dry - Swab - NR - NR |
| Duan 2021 ([89](#_ENREF_89)) (Cross-sectional) | Women living with HIV aged 18 years and older were recruited from HIV/AIDS treatment clinic in Yunnan, China from 2019 to 2020.  (n= 409) | - China - Rural - UMIC - Western Pacific Region - Eastern Asia - Unclassified | - Healthcare facility - Government-owned - Health Centre - NR | - Sansure; Cobas 4800 HPV DNA - Vaginal; Cervical - Wet - Brush - NR - ThinPrep, Hologic, USA | - Sansure; Cobas 4800; Hybrid Capture II HPV DNA - Cervical - Wet - Brush - NR - ThinPrep, Hologic, USA |
| Elliott 2019  ([90](#_ENREF_90)) (Cross-sectional) | Women living with HIV (WLWH) aged 25 or over presenting for routine appointments at the IDCC between March and April 2017  (n= 103) | - Botswana - NR - UMIC - African Region - Sub-Saharan Africa - Southern Africa | - Healthcare facility - Government-owned (public) - Hospital - NR | - GeneXpert HPV DNA - Vaginal - Wet - Swab - Nylon - NR | - GeneXpert HPV DNA - Cervical - Wet - Speculum & Brush - NR - NR |
| Feng 2022  ([91](#_ENREF_91)) (Cross-sectional) | Sexually active women at the nimrs antiretroviral therapy (ART) clinic and outpatient clinic in Lagos, Nigeria  (n= 213) | - Nigeria - NR - LMIC - African Region - Sub-Saharan Africa - Western Africa | - Healthcare facility - Government-owned - Health Centre - NR | - HPV DNA - NR - NR - NR - NR - NR | - HPV DNA - NR - NR - NR - NR - NR |
| Garcia 2003  ([92](#_ENREF_92)) (Cross-sectional) | All 18 years or older, scheduled for colposcopy examination, and able to provide written informed consent. Women with a history of hysterectomy, current pregnancy, or a history of vaginal trauma or laceration were excluded.  (n= 343) | - Mexico; Peru - NR - UMIC - Region of the Americas - Latin America and the Caribbean - Central America; South America | - Healthcare facility - Government-owned - Hospital - NR | - GeneAmp PCR System 9700 HPV DNA - Cervical - Wet - Brush - NR - Medscand, Malmo, Sweden | - GeneAmp PCR System 9700 HPV DNA - Cervical - Wet - Brush - NR - Medscand, Malmo, Sweden |
| Holanda 2006  ([93](#_ENREF_93)) (Cross-sectional) | Sexually active women, aged 15 to 70 and living in rural districts were identified by the the regional nurse supervisor and the chas. Pregnant women were excluded, as were those having had hysterectomies  (n= 878) | - Brazil - Rural - UMIC - Region of the Americas - Latin America and the Caribbean - South America | - Home-based - Not applicable - Not applicable - Home | - HPV DNA - Vaginal - Wet - Brush - NR - Digene Corporation, Gaithersburg, MD, USA | - NR - Ectocervical - Wet - Brush - NR - NR |
| Jeronimo 2014 ([94](#_ENREF_94)) (Cross-sectional) | Married women aged 25 to 60 years without a history of CIN or cervical cancer, a hysterectomy that removed the cervix, and any condition that would pose a health risk to the participant or interfere with the evaluation of the study objectives such as a history of bleeding or clotting disorders, debilitating physical and/or mental illness, and inability to provide informed consent. Pregnancy was considered a temporary exclusion until 3 months’ post delivery  (n= 19349) | - India; Nicaragua; Uganda - Urban; Peri-urban; Rural - LIC; LMIC - South-East Asia Region; Region of the Americas; African Region - Southern Asia; Latin America and the Caribbean; Sub-Saharan Africa - Unclassified; Central America; Eastern Africa | - Healthcare facility; Community-based - Government-owned (public) - Health Centre - Community health posts | - Care HPV DNA - Vaginal; Cervical - Wet - Swab - Cotton - NR | - Care HPV DNA - Cervical - Wet - Brush - NR - NR |
| Jones 2007 ([95](#_ENREF_95)) (cross-sectional) | Women aged 18 years or older, sexually active, self-reportedly not pregnant, and willing to comply with the protocol and gave written informed consent  (n= 450) | - South Africa - NR - UMIC - African Region - Sub-Saharan Africa - Southern Africa | - Healthcare facility - Government-owned - Health Centre - NR | - Hybrid Capture 2 (HC2) HPV DNA - Vaginal - Wet - Tampon or swab - NR - NR | - Hybrid Capture 2 (HC2) High risk HPV DNA - Cervix - NR - Brush-endocervical - NR - NR |
| Joseph 2021 ([96](#_ENREF_96)) (Cross-sectional) | HIV-positive women aged 30 to 49 years attending the pilot facilities for routine appointments. Women were excluded from the study if they were pregnant or less than 3 months postpartum, currently menstruating, previously diagnosed with cervical cancer, or had a hysterectomy  (n= 280) | - Zimbabwe - Urban - LMIC - African Region - Sub-Saharan Africa - Eastern Africa | - Healthcare facility - Government-owned (public) - Hospital - NR | - Aptima HPV mRNA - Vaginal; Cervical - Wet - Swab-Flock - Nylon - COPAN Diagnostics Inc, Murrieta, CA | - Aptima HPV mRNA - Cervical - Wet - Brush-cytobrush - NR - NR |
| Kamal 2014  ([97](#_ENREF_97)) (Cross-sectional) | Sexually active women 25 to 65 years old, not pregnant, with an intact uterus, and had no history of cervical intraepithelial neoplasia grade 2 or more severe (CIN2?) Disease or pelvic radiation  (n= 1601) | - Egypt, Arab Rep. - NR - LMIC - African Region - Northern Africa - Unclassified | - Healthcare facility - NR - Health Centre - NR | - HPV DNA - Cervical - Wet - Swab - Cotton - Digene Corporation, Gaithersburg, MD, USA | - HPV DNA - Cervical - Wet - Brush - NR - Digene Corporation, Gaithersburg, MD, USA |
| Longatto-Filho 2008  ([98](#_ENREF_98)) (Cohort) | In our ongoing multi-centre study in Latin America, a cohort of over 12,000 women have been examined using eight different diagnostic tests as potential screening tools in low-resource settings  (n= 1081) | - Argentina; Brazil - NR - UMIC - Region of the Americas - Latin America and the Caribbean - South America | - Healthcare facility - NR - Hospital - NR | - Hybrid Capture II HPV DNA - NR - NR - NR - NR - NR | - Hybrid Capture II HPV DNA - NR - NR - NR - NR - NR |
| Longatto-Filho 2012  ([99](#_ENREF_99)) (Cohort) | Consecutive women from the cities of Campinas (Brazil), So Paulo (Brazil), Porto Alegre (Brazil), and Buenos Aires (Argentina) were invited to gynecological consultations and tests examination  (n= 4725) | - Argentina; Brazil - NR - UMIC - Region of the Americas - Latin America and the Caribbean - South America | - Healthcare facility - NR - Health Centre - NR | - NR - HPV DNA - Cervical - NR - Tampon - NR - NR | - Provider-sampling - NR - HPV DNA - Cervical - NR - Swab - NR - NR |
| Lorenzato 2002 ([100](#_ENREF_100)) (Cross-sectional) | Any woman who came for screening at the Institute of Mother and Child Health in Pernambuco and the Cancer Hospital in Pernambuco, including pregnant women, without age restriction, who were randomly selected to participate. The exclusion criteria were women without a cervix and women who were too ill or unwilling to participate in the study  (n= 253) | - Brazil - NR - UMIC - Region of the Americas - Latin America and the Caribbean - South America | - Healthcare facility - Government-owned - Hospital - NR | - PGMY09/11 L1 consensus PCR HPV DNA - Vaginal; Cervical - Wet - Swab - Cotton - NR | - PGMY09/11 L1 consensus PCR HPV DNA - Cervical - Wet - Cytobrush - NR - Medscand |
| Nilyanimit 2014 ([101](#_ENREF_101))  (Cross-sectional) | Participations were voluntary and were solicited during colposcopy clinic and routine clinic. Both methods to collect the specimen were performed during the same visit for all participants  (n= 101) | - Thailand - NR - UMIC - South-East Asia Region - South-Eastern Asia - Unclassified | - Healthcare facility - Government-owned - Hospital - NR | - HPV DNA - Vaginal - Dry - Swab - Nylon - Copan Diagnostics, Murrieta, CA | - HPV DNA - Cervical - Dry - swab - NR - Copan Diagnostics, Murrieta, CA |
| Qin 2016 ([102](#_ENREF_102)) (Cross-sectional) | Aged over 18 years; not pregnant; did not have a history of diagnosed CIN, cervical cancer, or hysterectomy, could accept a pelvic exam mentally and physically  (n= 291) | - China - NR - UMIC - Western Pacific Region - Eastern Asia - Unclassified | - Healthcare facility - Government-owned - Hospital - NR | - Abbott HPV DNA - Cervicovaginal - dry - Brush - NR - Qiagen, Gaithersburg, USA | - Abbott HPV DNA - Cervicovaginal - Wet - brush - NR - NR |
| Quincy 2012  ([103](#_ENREF_103)) (Cross-sectional) | Non-pregnant, 25 to 60-year-old women with intact uteri, from Leon, Nicaragua  (n= 245) | - Nicaragua - NR - LMIC - Region of the Americas - Latin America and the Caribbean - Central America | - Healthcare facility; Community-based - Government-owned (public) - Health Centre - Community health posts | - Hybrid Capture II HPV DNA - vaginal - NR - swab - NR - NR | - Hybrid Capture 2 HPV DNA - cervical - wet - brush - NR - NR |
| Safaeian 2007 ([104](#_ENREF_104)) (Cross-sectional) | Two thousand five hundred women who had provided self-collected swabs during the follow-up visits were identified and targeted for this sub-study  (n= 606) | - Uganda - Rural - Low -income country - African Region - Sub-Saharan Africa - Eastern Africa | - Healthcare facility - Government-owned - Health Centre - NR | - Hybrid capture II HPV DNA - Vaginal - Wet - Swab - NR - Digene Sampler Kit - (Digene Corp., Gaithersburg, MD) | - Hybrid capture II HPV DNA - Cervical - NR - NR - NR - NR |
| Salmeron 2003  ([105](#_ENREF_105)) (Cross-sectional) | All women attending CC screening services at any one of the 23 health units that make up the Morelos CCSP.  (n= 7868) | - Mexico - Urban; Peri-urban - UMIC - Region of the Americas - Latin America and the Caribbean - Central America | - Healthcare facility - Government-owned - Health Centre - NR | - Hybrid Capture II HPV DNA - Vaginal - Dry - Swab - Cotton - NR | - Hybrid Capture 2 (HC2) HPV DNA - Cervical - Dry - Brush - NR - Digene, Gaithersburg, MD |
| Senkomago 2018 ([106](#_ENREF_106)) (Cohort) | The study participants were aged at least 18 years, were not in the second or third trimester of pregnancy, and had an intact cervix  (n= 344) | - Kenya - NR - LMIC - African Region - Sub-Saharan Africa - Eastern Africa | - NR - NR - NR - NR | - Aptima - HPV DNA - Cervicovaginal - NR - Brush - NR - Hologic, Marlborough, MA, USA | - Aptima HPV mRNA - Cervical - NR - NR - NR - NR |
| Sowjanya 2009  ([107](#_ENREF_107)) (Cross-sectional) | Women 25 years or older, had an intact uterus, were mentally competent, and were able and willing to provide informed consent  (n= 432) | - India - NR - LMIC - South-East Asia Region - Southern Asia - Unclassified | - Community-based - Not applicable - Not applicable - Community health posts | - Hybrid Capture II HPV DNA - Vaginal - Wet - Swab - NR - NR | - Hybrid Capture II HPV DNA - Cervical - NR - Swab - NR - NR |
| Tiiti 2021 ([108](#_ENREF_108)) (cross-sectional) | Women who were 18 years and older attending gynaecology clinics at a tertiary hospital in Pretoria, South Africa  (n= 527) | - South Africa - NR - UMIC - African Region - Sub-Saharan Africa - Southern Africa | - Healthcare facility - Government-owned (public) - Hospital - NR | - Aptima; Abbott HPV DNA - Vaginal - Wet - Tampon - Cotton - Hologic Gen-Probe, Inc., San Diego, Canada) | - Abott HPV DNA - Cervical - Wet - Brush - Hologic Inc., Marlborough, USA |
| Toliman 2016  ([109](#_ENREF_109)) (Cross-sectional) | Women aged 30 to 59 years attending clinics for routine cervical screening were provided information about the study while waiting to be seen  (n= 1005) | - Papua New Guinea - NR - LMIC - Western Pacific Region - Melanesia - Unclassified | - Healthcare facility - Government-owned (public) - Health Centre - NR | - GeneXpert HPV DNA - Vaginal - Wet - Brush - NR - NR | - GeneXpert HPV DNA - Cervical - Wet - brush - NR - NR |
| Toliman 2019 ([110](#_ENREF_110))  (Cross-sectional) | Women aged 30 to 54 years (the target age group for cervical screening in Papua New Guinea)  (n= 1005) | - Papua New Guinea - NR - LMIC - Western Pacific Region - Melanesia - Unclassified | - Healthcare facility - NR - Health Centre - NR | - GeneXpert HPV DNA - Vaginal - Wet - Brush - NR - NR | - GeneXpert HPV DNA - cervical - Wet - brush - NR - NR |
| VegaCrespo 2022  ([111](#_ENREF_111)) (Cross-sectional) | Sexually active women aged 18-70 years old; not having undergone an excision or destructive treatment of the cervical intraepithelial neoplasm; not having used vaginal medication at least a week before the examination; not having had sexual intercourse for at least 48 h previous to the examination; not being pregnant; and the absence of menstrual bleeding at the time of examination  (n= 120) | - Ecuador - Rural - UMIC - Region of the Americas - Latin America and the Caribbean - South America | - Healthcare facility - NR - Health Centre - NR | - HPV DNA - Vaginal - NR - Brush - NR - Rovers Medical Devices, Oss, The Netherlands | - HPV DNA - cervical - NR - brush - NR - Rovers Medical Devices |
| Viviano 2018  ([112](#_ENREF_112)) (Cross-sectional) | All women aged 30 to 49 years were recruited in a CC screening campaign  (n= 188) | - Cameroon - NR - LMIC - African Region - Sub-Saharan Africa - Middle Africa | - Healthcare facility - NR - Hospital - NR | - GeneXpert HPV DNA - Vaginal - Dry - Swab - NR - NR | - GeneXpert HPV DNA - cervical - wet - brush - NR - Rovers, Oss, the Netherlands |
| Wright 2000  ([113](#_ENREF_113)) (Cross-sectional) | Women aged 35 to 65 years  (n= 2830) | - South Africa - Peri-urban - UMIC - African Region - Sub-Saharan Africa - Southern Africa | - Healthcare facility - Government-owned (public) - Health Centre - NR | - Hybrid capture II HPV DNA - Vaginal - NR - Swab - Dacron - NR | - Hybrid Capture II HPV DNA - Cervical - dry - Brush - NR - NR |
| Lack 2005  ([114](#_ENREF_114)) (Cross-sectional) | Women who had previously been screened for HPV in a reproductive morbidity survey (RMS) were recruited from a rural area of the Gambia  (n= 377) | - Gambia, The - Rural - LIC - African Region - Sub-Saharan Africa - Western Africa | - NR - NR - NR - NR | - HPV DNA - Vaginal - Wet - Swab - Dacron - NR | - HPV DNA - Cervical - Wet - Brush - NR - Qiagen Ltd, Crawley, UK |
| Costing | | | | | |
| Campos 2017  ([115](#_ENREF_115)) (Modeling) | Women aged 30 to 49 years  (n= 130) | - Uganda - NR - LIC - African Region - Sub-Saharan Africa - Eastern Africa | - Healthcare facility; Community-based - Government-owned (public) - Health Centre - Community health posts | - HPV DNA - NR - NR - NR - NR - NR | - NR - NR - NR - NR - NR - NR |
| Campos 2019  ([116](#_ENREF_116)) (Modeling) | Data from phase 2 of the CAPE demonstration were used to inform a mathematical model of HPV infection and cervical cancer  (n= 8000) | - El Salvador - NR - LMIC - Region of the Americas - Latin America and the Caribbean - Central America | - NR - NR - NR - NR | - HPV DNA - NR - NR - NR - NR - NR | - NR - NR - NR - NR - NR - NR |
| Flores 2011  ([117](#_ENREF_117)) (Modeling) | Female clients from the Mexican Institute of Social Security, between the ages of 20 to 80, in the state of Morelos  (n= NR) | - Mexico - NR - UMIC - Region of the Americas - Latin America and the Caribbean - Central America | - NR - NR - NR - NR | - HPV DNA - NR - NR - NR - NR - NR | - NR/ NR - NR - NR - NR - NR - NR |
| Olwanda 2020  ([118](#_ENREF_118)) (Microcosting) | Women aged between 25 and 65 years who did not screen at the chcs were offered home-based screening in November 2018  (n= 3299) | - Kenya - Rural - LMIC - African Region - Sub-Saharan Africa - Eastern Africa | - Community-based - Not applicable - Not applicable - Community health posts | - Care HPV DNA - Vaginal - Wet - brush - NR - NR | - Care HPV DNA - vaginal - Wet - Brush - NR - NR |
| Shi 2012  ([119](#_ENREF_119)) (Cross-sectional) | Direct medical and non-medical costs related to cervical screening, diagnosis and treatment of cervical high grade lesions and cervical cancer were collected in this study  (n= NR) | - China - Rural - UMIC - Western Pacific Region - Eastern Asia - Unclassified | - Healthcare facility - Government-owned (public) - Hospital - NR | - HPV DNA - NR - NR - NR - NR - NR | - NR - NR - NR - NR - NR - NR |
| Zhao 2023  ([120](#_ENREF_120)) (Modeling) | Women aged 30 to 65 years in urban and rural China, with a 5-year screening interval  (n= NR) | - China - Urban; Rural - UMIC - Western Pacific Region - Eastern Asia - Unclassified | - NR - NR - NR - NR | - HPV DNA - NR - NR - NR - NR - NR | - NR - NR - NR - NR - NR - NR |
| Uptake | | | | | |
| Arrossi 2015  ([121](#_ENREF_121)) (Cluster randomized controlled trial) | Women 30 years or older and living in a household visited by community health workers  (n= 6013) | - Argentina - Rural - UMIC - Region of the Americas - Latin America and the Caribbean - South America | - Healthcare facility; Community-based - Government-owned (public) - Health Centre - Community health posts | - HPV DNA - Vaginal - Wet - Brush - NR - Qiagen, Gaithersburg, Maryland, USA | - NR - NR - NR - NR - NR - NR |
| Arrossi 2017  ([122](#_ENREF_122)) (Cross-sectional) | Women aged 30 years and above  (n= 5657) | - Argentina - NR - UMIC - Region of the Americas - Latin America and the Caribbean - South America | - Home-based - Not applicable - Not applicable - Home | - HPV DNA - NR - NR - Brush - NR - Qiagen, Gaithersburg, Maryland, USA | - NR - NR - NR - NR - NR - NR |
| Gizaw 2019  ([123](#_ENREF_123)) (Cluster randomized controlled trial) | All women included in this study had never been screened before. We used a total of 22 clusters, each comprising 80 women as a minimum required sample. The clusters were divided equally between two arms: self-collected HPV testing and VIA. Women were excluded if they were pregnant, actively bleeding, had a previous hysterectomy, and refused to give consent before the screening.  (n= 1299) | - Ethiopia - Urban; Rural - LIC - African Region - Sub-Saharan Africa - Eastern Africa | - Healthcare facility - Government-owned (public) - Hospital - NR | - HPV DNA - Cervical - Dry - Brush - Polyethylene - Rovers Medical Devices, Netherlands | - NR - Cervical - NR - NR - NR - NR |
| Moses 2015  ([124](#_ENREF_124)) (Individual randomized controlled trial) | Women aged 30 to 65 years of age, lived or worked in Kisenyi, and had access to a mobile telephone  (n= 500) | - Uganda - NR - LIC - African Region - Sub-Saharan Africa - Eastern Africa | - Healthcare facility; Community-based - Government-owned (public) - Hospital; Health Centre - Community health posts | - HPV DNA - Vaginal - Dry - Swab - Nylon - NR | - NR - NR - NR - NR - NR - NR |

LIC: low-income country, LMIC: lower-middle-income country, UMIC: upper-middle-income country, HPV DNA/mRNA: human papillomavirus deoxyribonucleic acid test/ ribonucleic acid assay, NR: not reported.

# **References**

1. Afzal O., Lieb W., Lieber M., Chowdhury S., Beddoe A.M. Cervical cancer risk factors and screening preferences among Muslim women in Monrovia, Liberia. Afr J Reprod Health. 2020;24(3):101-7 doi:<https://dx.doi.org/10.29063/ajrh2020/v24i3.11>.

2. Ahmad Z.A., Daud S., Abdullah N.N., Omar N.S. Perception and knowledge of human papillomavirus (HPV) and HPV DNA self-sampling amongst women in west Malaysia. Brunei International Medical Journal (BIMJ). 2021;17:79-85.

3. Allende G., Surriabre P., Caceres L., Bellot D., Ovando N., Torrico A., et al. Evaluation of the self-sampling for cervical cancer screening in Bolivia. BMC Public Health. 2019;19(1):80 doi:<https://dx.doi.org/10.1186/s12889-019-6401-5>.

4. Allen-Leigh B., Uribe-Zuniga P., Leon-Maldonado L., Brown B.J., Lorincz A., Salmeron J., et al. Barriers to HPV self-sampling and cytology among low-income indigenous women in rural areas of a middle-income setting: a qualitative study. BMC Cancer. 2017;17(1):734 doi:<https://dx.doi.org/10.1186/s12885-017-3723-5>.

5. Arrossi S., Ramos S., Straw C., Thouyaret L., Orellana L. HPV testing: a mixed-method approach to understand why women prefer self-collection in a middle-income country. BMC Public Health. 2016;16:832 doi:<https://dx.doi.org/10.1186/s12889-016-3474-2>.

6. Awua A.K., Wiredu E.K., Afari E.A., Tijani A.S., Djanmah G., Adanu R.M.K. A tailored within-community specimen collection strategy increased uptake of cervical cancer screening in a cross-sectional study in Ghana. BMC Public Health. 2017;18(1):80 doi:<https://dx.doi.org/10.1186/s12889-017-4631-y>.

7. Bakiewicz A., Rasch V., Mwaiselage J., Linde D.S. "The best thing is that you are doing it for yourself" - perspectives on acceptability and feasibility of HPV self-sampling among cervical cancer screening clients in Tanzania: a qualitative pilot study. BMC Women's Health. 2020;20(1):1-9 doi:10.1186/s12905-020-00917-7.

8. Berner A., Hassel S.B., Tebeu P.M., Untiet S., Kengne-Fosso G., Navarria I., et al. Human papillomavirus self-sampling in Cameroon: women's uncertainties over the reliability of the method are barriers to acceptance. J. 2013;17(3):235-41 doi:<https://dx.doi.org/10.1097/LGT.0b013e31826b7b51>.

9. Brandt T., Wubneh S.B., Handebo S., Debalkie G., Ayanaw Y., Alemu K., et al. Genital self-sampling for HPV-based cervical cancer screening: a qualitative study of preferences and barriers in rural Ethiopia. BMC Public Health. 2019;19(1):N.PAG-N.PAG doi:10.1186/s12889-019-7354-4.

10. Broquet C., Triboullier D., Untiet S., Schafer S., Petignat P., Vassilakos P. Acceptability of self-collected vaginal samples for HPV testing in an urban and rural population of Madagascar. Afr Health Sci. 2015;15(3):755-61 doi:<https://dx.doi.org/10.4314/ahs.v15i3.8>.

11. Castle P.E., Silva V.R.S., Consolaro M.E.L., Kienen N., Bittencourt L., Pelloso S.M., et al. Participation in Cervical Screening by Self-collection, Pap, or a Choice of Either in Brazil. Cancer Prev Res (Phila Pa). 2019;12(3):159-70 doi:<https://dx.doi.org/10.1158/1940-6207.CAPR-18-0419>.

12. Dareng E.O., Jedy-Agba E., Bamisaye P., Modibbo F.I., Oyeneyin L.O., Adewole A.S., et al. Influence of Spirituality and Modesty on Acceptance of Self-Sampling for Cervical Cancer Screening. PLoS One. 2015;10(11):12 doi:10.1371/journal.pone.0141679.

13. Dzuba I.G., Turnbull B., Avila M.H., Salmeron J., Diaz E.Y., Allen B., et al. The acceptibility of self-collected samples for HPV testing vs. the pap test as alternatives in cervical cancer screening. Journal of Women's Health and Gender-Based Medicine. 2002;11(3):265-75 doi:<https://dx.doi.org/10.1089/152460902753668466>.

14. Eche M.T., Vermaak K. Knowledge, attitude and practice of female university students regarding human papillomavirus and self-sampling in KwaZulu-Natal, South Africa: a cross-sectional survey. BMC Womens Health. 2022;22(1):58 doi:<https://dx.doi.org/10.1186/s12905-022-01634-z>.

15. Esber A., McRee A.L., Norris Turner A., Phuka J., Norris A. Factors influencing Malawian women's willingness to self-collect samples for human papillomavirus testing. J Fam Plann Reprod Health Care. 2017;43(2):135-41 doi:<https://dx.doi.org/10.1136/jfprhc-2015-101305>.

16. Flores Y., Bishai D., Lazcano E., Shah K., Lorincz A., Hernandez M., et al. Improving cervical cancer screening in Mexico: results from the Morelos HPV Study. Salud Publica Mex. 2003;45 Suppl 3:S388-98.

17. Goldstein A., Plafker B., Stamper S., Goldstein L., Lipson R., Bedell S., et al. Patient Satisfaction With Human Papillomavirus Self-Sampling in a Cohort of Ethnically Diverse and Rural Women in Yunnan Province, China. Journal of Lower Genital Tract Disease. 2020;24(4):349-52 doi:10.1097/LGT.0000000000000560.

18. Gottschlich A., Rivera-Andrade A., Grajeda E., Alvarez C., Mendoza Montano C., Meza R. Acceptability of Human Papillomavirus Self-Sampling for Cervical Cancer Screening in an Indigenous Community in Guatemala. J. 2017;3(5):444-54 doi:<https://dx.doi.org/10.1200/JGO.2016.005629>.

19. Gottschlich A., Nuntadusit T., Zarins K.R., Hada M., Chooson N., Bilheem S., et al. Barriers to cervical cancer screening and acceptability of HPV self-testing: a cross-sectional comparison between ethnic groups in Southern Thailand. BMJ Open. 2019;9(11):e031957 doi:<https://dx.doi.org/10.1136/bmjopen-2019-031957>.

20. Guan Y., Castle P.E., Wang S., Li B., Feng C., Ci P., et al. A cross-sectional study on the acceptability of self-collection for HPV testing among women in rural China. Sex Transm Infect. 2012;88(7):490-4 doi:<https://dx.doi.org/10.1136/sextrans-2012-050477>.

21. He L., He J. Attitudes towards HPV self-sampling among women in Chengdu, China: A cross-sectional survey. Journal of Medical Screening. 2020;27(4):201-6 doi:10.1177/0969141319895543.

22. Hood R.B., Turner A.N., Huber-Krum S., Lancaster K.E., Mwapasa V., Poindexter T., et al. For Human Papillomavirus Self-Sampling, Stated Willingness Does Not Correspond With Subsequent Uptake by Rural Malawian Women. Sex Transm Dis. 2020;47(4):275-9 doi:<https://dx.doi.org/10.1097/OLQ.0000000000001119>.

23. Huchko M.J., Ibrahim S., Blat C., Cohen C.R., Smith J.S., Hiatt R.A., et al. Cervical cancer screening through human papillomavirus testing in community health campaigns versus health facilities in rural western Kenya. Int J Gynaecol Obstet. 2018;141(1):63-9 doi:<https://dx.doi.org/10.1002/ijgo.12415>.

24. Khoo S.P., Lim W.T., Rajasuriar R., Nasir N.H., Gravitt P., Woo Y.L. The Acceptability and Preference of Vaginal Self-sampling for Human Papillomavirus (HPV) Testing among a Multi-ethnic Asian Female Population. Cancer Prev Res (Phila Pa). 2021;14(1):105-12 doi:<https://dx.doi.org/10.1158/1940-6207.CAPR-20-0280>.

25. Kohler R.E., Elliott T., Monare B., Moshashane N., Ramontshonyana K., Chatterjee P., et al. HPV self-sampling acceptability and preferences among women living with HIV in Botswana. Int J Gynaecol Obstet. 2019;147(3):332-8 doi:<https://dx.doi.org/10.1002/ijgo.12963>.

26. Laskow B., Figueroa R., Alfaro K.M., Scarinci I.C., Conlisk E., Maza M., et al. A pilot study of community-based self-sampling for HPV testing among non-attenders of cervical cancer screening programs in El Salvador. Int J Gynaecol Obstet. 2017;138(2):194-200 doi:<https://dx.doi.org/10.1002/ijgo.12204>.

27. Li J., Wu R., Qu X., Huang X., Li L., Lin Z., et al. Effectiveness and feasibility of self-sampling for human papillomavirus testing for internet-based cervical cancer screening. Front. 2022;10:938272 doi:<https://dx.doi.org/10.3389/fpubh.2022.938272>.

28. Mahande M.J., Oneko O., Amour C., Pollie M., Smith C., Mboya I.B., et al. Feasibility and acceptability of human papillomavirus self-sampling in a semi-urban area in northern Tanzania. Int J Gynaecol Obstet. 2021;154(1):113-8 doi:<https://dx.doi.org/10.1002/ijgo.13579>.

29. Mahomed K., Evans D., Sauls C., Richter K., Smith J., Firnhaber C. Human papillomavirus (HPV) testing on self-collected specimens: perceptions among HIV positive women attending rural and urban clinics in South Africa. Pan Afr Med J. 2014;17:189 doi:<https://dx.doi.org/10.11604/pamj.2014.17.189.3454>.

30. Mandigo M., Frett B., Laurent J.R., Bishop I., Raymondville M., Marsh S., et al. Pairing community health workers with HPV self-sampling for cervical cancer prevention in rural Haiti. Int J Gynaecol Obstet. 2015;128(3):206-10 doi:<https://dx.doi.org/10.1016/j.ijgo.2014.09.016>.

31. Manguro G.O., Masese L.N., Mandaliya K., Graham S.M., McClelland R.S., Smith J.S. Preference of specimen collection methods for human papillomavirus detection for cervical cancer screening: a cross-sectional study of high-risk women in Mombasa, Kenya. Reprod Health. 2018;15:8 doi:10.1186/s12978-018-0651-z.

32. Maza M., Melendez M., Masch R., Alfaro K., Chacon A., Gonzalez E., et al. Acceptability of self-sampling and human papillomavirus testing among non-attenders of cervical cancer screening programs in El Salvador. Prev Med. 2018;114:149-55 doi:<https://dx.doi.org/10.1016/j.ypmed.2018.06.017>.

33. Maza M., Melendez M., Herrera A., Hernandez X., Rodriguez B., Soler M., et al. Cervical Cancer Screening with Human Papillomavirus Self-Sampling Among Transgender Men in El Salvador. LGBT health. 2020;7(4):174-81 doi:<https://dx.doi.org/10.1089/lgbt.2019.0202>.

34. McFarlane S.J., Morgan S.E. Evaluating Culturally-targeted Fear Appeal Messages for HPV Self-Sampling among Jamaican Women: A Qualitative Formative Research Study. Health Communication. 2021;36(7):877-90 doi:10.1080/10410236.2020.1723047.

35. Megersa B.S., Bussmann H., Barnighausen T., Muche A.A., Alemu K., Deckert A. Community cervical cancer screening: Barriers to successful home-based HPV self-sampling in Dabat district, North Gondar, Ethiopia. A qualitative study. PLoS ONE. 2020;15(12):e0243036 doi:<https://dx.doi.org/10.1371/journal.pone.0243036>.

36. Mitchell S., Ogilvie G., Steinberg M., Sekikubo M., Biryabarema C., Money D. Assessing women's willingness to collect their own cervical samples for HPV testing as part of the ASPIRE cervical cancer screening project in Uganda. Int J Gynaecol Obstet. 2011;114(2):111-5 doi:<https://dx.doi.org/10.1016/j.ijgo.2011.01.028>.

37. Mitchell S.M., Pedersen H.N., Eng Stime E., Sekikubo M., Moses E., Mwesigwa D., et al. Self-collection based HPV testing for cervical cancer screening among women living with HIV in Uganda: a descriptive analysis of knowledge, intentions to screen and factors associated with HPV positivity. BMC Womens Health. 2017;17(1):4 doi:<https://dx.doi.org/10.1186/s12905-016-0360-0>.

38. Mremi A., Linde D.S., McHome B., Mlay J., Schledermann D., Blaakaer J., et al. Acceptability and feasibility of self-sampling and follow-up attendance after text message delivery of human papillomavirus results: A cross-sectional study nested in a cohort in rural Tanzania. Acta Obstet Gynecol Scand. 2021;100(4):802-10 doi:<https://dx.doi.org/10.1111/aogs.14117>.

39. Murchland A.R., Gottschlich A., Bevilacqua K., Pineda A., Sandoval-Ramirez B.A., Alvarez C.S., et al. HPV self-sampling acceptability in rural and indigenous communities in Guatemala: a cross-sectional study. BMJ Open. 2019;9(10):e029158 doi:<https://dx.doi.org/10.1136/bmjopen-2019-029158>.

40. Nyabigambo A., Mayega R.W., Hlongwana K., Ginindza T.G. Facilitators and Barriers to HPV Self-Sampling as a Cervical Cancer Screening Option among Women Living with HIV in Rural Uganda. Int J Environ Res Public Health. 2023;20(11):30 doi:<https://dx.doi.org/10.3390/ijerph20116004>.

41. Oberlin A., Pasipamire T., Chibwesha C.J. Exploring women's preferences for HPV-based cervical cancer screening using a discrete choice experiment. American Journal of Obstetrics and Gynecology. 2018;219(6):635-6 doi:<https://dx.doi.org/10.1016/j.ajog.2018.10.070>.

42. Oketch S.Y., Kwena Z., Choi Y., Adewumi K., Moghadassi M., Bukusi E.A., et al. Perspectives of women participating in a cervical cancer screening campaign with community-based HPV self-sampling in rural western Kenya: a qualitative study. BMC Women's Health. 2019;19(1):N.PAG-N.PAG doi:10.1186/s12905-019-0778-2.

43. Oneko O., Mahande M.J., Amour C., Pollie M., Smith C., Mboya I.B., et al. Willingness to HPV self-sampling for cervical cancer screening and its predictors among women attending outpatient clinics in Meru District, Arusha Region, Northern Tanzania. Afr Health Sci. 2022;22(2):97-106 doi:<https://dx.doi.org/10.4314/ahs.v22i2.12>.

44. Oranratanaphan S., Termrungruanglert W., Khemapech N. Acceptability of self-sampling HPV testing among Thai women for cervical cancer screening. Asian Pac J Cancer Prev. 2014;15(17):7437-41.

45. Peedicayil A., Abraham P., Prasad J., Abraham S., Jeyaseelan L., Gnanamony M., et al. The community prevalence of human papillomavirus (HPV) in India and the feasibility of self-collected vaginal swabs. Gynecologic Oncology. 2014;1):15 doi:<https://dx.doi.org/10.1016/j.ygyno.2014.03.058>.

46. Phoolcharoen N., Kantathavorn N., Krisorakun W., Taepisitpong C., Krongthong W., Saeloo S. Acceptability of Self-Sample Human Papillomavirus Testing Among Thai Women Visiting a Colposcopy Clinic. J Community Health. 2018;43(3):611-5 doi:<https://dx.doi.org/10.1007/s10900-017-0460-2>.

47. Possati-Resende J.C., Vazquez F.D., Pantano N.D., Fregnani J., Mauad E.C., Longatto A. Implementation of a Cervical Cancer Screening Strategy Using HPV Self-Sampling for Women Living in Rural Areas. Acta Cytol. 2020;64(1-2):7-15 doi:10.1159/000493333.

48. Qu S.X., Ni Y.H., Qin J.F., Chen X.Y., Wu W.L., Zhang W.C. Experience and acceptability for HPV self-sampling among women in Jiangsu province, China: a cross-sectional survey. J Obstet Gynaecol. 2023;43(1):6 doi:10.1080/01443615.2023.2204942.

49. Quincy B.L., Turbow D.J., Dabinett L.N. Acceptability of self-collected human papillomavirus specimens as a primary screen for cervical cancer. J Obstet Gynaecol. 2012;32(1):87-91 doi:<https://dx.doi.org/10.3109/01443615.2011.625456>.

50. Rawat A., Sanders C., Mithani N., Amuge C., Pedersen H., Namugosa R., et al. Acceptability and preferences for self-collected screening for cervical cancer within health systems in rural Uganda: A mixed-methods approach. Int J Gynecol Obstet. 2021;152(1):103-11 doi:10.1002/ijgo.13454.

51. Rodrigues L.L.S., Morgado M.G., Sahasrabuddhe V.V., De Paula V.S., Oliveira N.S., Chavez-Juan E., et al. Cervico-vaginal self-collection in HIV-infected and uninfected women from Tapajos region, Amazon, Brazil: High acceptability, hrHPV diversity and risk factors. Gynecol Oncol. 2018;151(1):102-10 doi:10.1016/j.ygyno.2018.08.004.

52. Rosenbaum A.J., Gage J.C., Alfaro K.M., Ditzian L.R., Maza M., Scarinci I.C., et al. Acceptability of self-collected versus provider-collected sampling for HPV DNA testing among women in rural El Salvador. International Journal of Gynecology & Obstetrics. 2014;126(2):156-60 doi:10.1016/j.ijgo.2014.02.026.

53. Rositch A.F., Gatuguta A., Choi R.Y., Guthrie B.L., Mackelprang R.D., Bosire R., et al. Knowledge and Acceptability of Pap Smears, Self-Sampling and HPV Vaccination among Adult Women in Kenya. PLoS One. 2012;7(7):9 doi:10.1371/journal.pone.0040766.

54. Saidu R., Moodley J., Tergas A., Momberg M., Boa R., Wright T., et al. South African women's perspectives on self-sampling for cervical cancer screening: A mixed-methods study. SAMJ S Afr Med J. 2019;109(1):47-52 doi:10.7196/SAMJ.2019.v109i1.13278.

55. Sormani J., Kenfack B., Wisniak A., Datchoua A.M., Makajio S.L., Schmidt N.C., et al. Exploring Factors Associated with Patients Who Prefer Clinician-Sampling to HPV Self-Sampling: A Study Conducted in a Low-Resource Setting. Int J Environ Res Public Health. 2022;19(1):11 doi:10.3390/ijerph19010054.

56. Vallely A.J.B., Saville M., Badman S.G., Gabuzzi J., Bolnga J., Mola G.D.L., et al. Point-of-care HPV DNA testing of self-collected specimens and same-day thermal ablation for the early detection and treatment of cervical pre-cancer in women in Papua New Guinea: a prospective, single-arm intervention trial (HPV-STAT). Lancet Glob Health. 2022;10(9):e1336-e46 doi:<https://dx.doi.org/10.1016/S2214-109X(22)00271-6>.

57. Varun N., Hallie D., Marianne V., Maryam A., Barbra M., McKerron S., et al. Understanding the cervical cancer self-collection preferences of women living in urban and rural Rwanda. medRxiv. 2023:2023.06.15.23291471 doi:10.1101/2023.06.15.23291471.

58. Vega Crespo B., Neira V.A., Ortiz S.J., Maldonado-Rengel R., Lopez D., Gomez A., et al. Evaluation of Urine and Vaginal Self-Sampling versus Clinician-Based Sampling for Cervical Cancer Screening: A Field Comparison of the Acceptability of Three Sampling Tests in a Rural Community of Cuenca, Ecuador. Healthcare (Basel). 2022;10(9):25 doi:<https://dx.doi.org/10.3390/healthcare10091614>.

59. Yoshida T., Nishijima Y., Hando K., Vilayvong S., Arounlangsy P., Fukuda T. Primary Study on Providing a Basic System for Uterine Cervical Screening in a Developing Country: Analysis of Acceptability of Self-sampling in Lao PDR. Asian Pac J Cancer Prev. 2013;14(5):3029-35 doi:10.7314/apjcp.2013.14.5.3029.

60. Adamson P.C., Huchko M.J., Moss A.M., Kinkel H.F., Medina-Marino A. Acceptability and accuracy of cervical cancer screening using a self-collected tampon for HPV messenger-RNA testing among HIV infected women in South Africa. PLoS ONE. 2015;10(9) (no pagination)(e0137299) doi:<https://dx.doi.org/10.1371/journal.pone.0137299>.

61. Anand K.V., Mishra G.A., Pimple S.A., Biswas S.K., Kulkarni V.Y., Salunke G. Cross-Sectional Study of HPV Self-Sampling among Indian Women-A Way Forward. Indian Journal of Medical and Paediatric Oncology. 2022;43(1):103-8 doi:<https://dx.doi.org/10.1055/s-0042-1742642>.

62. de Melo Kuil L., Lorenzi A.T., Stein M.D., Resende J.C.P., Antoniazzi M., Longatto-Filho A., et al. The Role of Self-Collection by Vaginal Lavage for the Detection of HPV and High-Grade Intraepithelial Neoplasia. Acta Cytol. 2017;61(6):425-33 doi:<https://dx.doi.org/10.1159/000477331>.

63. Eamratsameekool W., Phumiressunthon K., Sukprasert L., Pukdeesamai P. Comparison of Self- To Provider-Collected Cervical Screening with HPV DNA Test at Roi Et Province, Thailand during COVID-19 Pandemic. Journal of the Medical Association of Thailand. 2023;106(1):8-13 doi:<https://dx.doi.org/10.35755/jmedassocthai.2023.01.13727>.

64. Esber A., Norris A., Jumbe E., Kandodo J., Nampandeni P., Reese P.C., et al. Feasibility, validity and acceptability of self-collected samples for human papillomavirus (HPV) testing in rural Malawi. Malawi Med J. 2018;30(2):61-6 doi:<https://dx.doi.org/10.4314/mmj.v30i2.2>.

65. Aranda Flores C.E., Gomez Gutierrez G., Ortiz Leon J.M., Cruz Rodriguez D., Sørbye S.W. Self-collected versus clinician-collected cervical samples for the detection of HPV infections by 14-type DNA and 7-type mRNA tests. BMC Infect Dis. 2021;21(1):504 doi:10.1186/s12879-021-06189-2.

66. Haile E.L., Cindy S., Ina B., Belay G., Jean-Pierre V.G., Sharon R., et al. HPV testing on vaginal/cervical nurse-assisted self-samples versus clinician-taken specimens and the HPV prevalence, in Adama Town, Ethiopia. Medicine (Baltimore). 2019;98(35):e16970 doi:<https://dx.doi.org/10.1097/MD.0000000000016970>.

67. Islam J.Y., Mutua M.M., Kabare E., Manguro G., Hudgens M.G., Poole C., et al. High-risk Human Papillomavirus Messenger RNA Testing in Wet and Dry Self-collected Specimens for High-grade Cervical Lesion Detection in Mombasa, Kenya. Sex Transm Dis. 2020;47(7):464-72 doi:<https://dx.doi.org/10.1097/OLQ.0000000000001167>.

68. Katanga J.J., Rasch V., Manongi R., Pembe A.B., Mwaiselage J.D., Kjaer S.K. Concordance in hpv detection between self-collected and health provider-collected cervicovaginal samples using carehpv in tanzanian women. JCO Global Oncology. 2021;7:985-91 doi:<https://dx.doi.org/10.1200/GO.20.00598>.

69. Madhivanan P., Nishimura H., Ravi K., Pope B., Coudray M., Arun A., et al. Acceptability and Concordance of Self- Versus Clinician- Sampling for HPV Testing among Rural South Indian Women. Asian Pac J Cancer Prev. 2021;22(3):971-6 doi:<https://dx.doi.org/10.31557/APJCP.2021.22.3.971>.

70. Obiri-Yeboah D., Adu-Sarkodie Y., Djigma F., Hayfron-Benjamin A., Abdul L., Simpore J., et al. Self-collected vaginal sampling for the detection of genital human papillomavirus (HPV) using careHPV among Ghanaian women. BMC Womens Health. 2017;17(1):86 doi:<https://dx.doi.org/10.1186/s12905-017-0448-1>.

71. Saidu R., Kuhn L., Tergas A., Boa R., Moodley J., Svanholm-Barrie C., et al. Performance of Xpert HPV on Self-collected Vaginal Samples for Cervical Cancer Screening among Women in South Africa. Journal of Lower Genital Tract Disease. 2021;25(1):15-21 doi:<https://dx.doi.org/10.1097/LGT.0000000000000575>.

72. Taku O., Meiring T.L., Gustavsson I., Phohlo K., Garcia-Jardon M., Mbulawa Z.Z.A., et al. Acceptability of self- collection for human papillomavirus detection in the Eastern Cape, South Africa. PLoS ONE. 2020;15(11):e0241781 doi:<https://dx.doi.org/10.1371/journal.pone.0241781>.

73. Tiiti T.A., Mashishi T.L., Nkwinika V.V., Molefi K.A., Benoy I., Bogers J., et al. Evaluation of ILEX SelfCerv for Detection of High-Risk Human Papillomavirus Infection in Gynecology Clinic Attendees at a Tertiary Hospital in South Africa. J Clin Med. 2021;10(21):12 doi:10.3390/jcm10214817.

74. Van De Wijgert J., Altini L., Jones H., De Kock A., Young T., Williamson A.L., et al. Two methods of self-sampling compared to clinician sampling to detect reproductive tract infections in Gugulethu, South Africa. Sexually Transmitted Diseases. 2006;33(8):516-23 doi:<https://dx.doi.org/10.1097/01.olq.0000204671.62529.1f>.

75. Wong E.L.Y., Cheung A.W.L., Fenwei H., Chor J.S.Y. Can Human Papillomavirus DNA Self-sampling be an Acceptable and Reliable Option for Cervical Cancer Screening in Female Sex Workers? Cancer Nursing. 2018;41(1):45-52 doi:10.1097/NCC.0000000000000462.

76. Abdullah N.N., Daud S., Wang S.M., Mahmud Z., Mohd Kornain N.K., Al-Kubaisy W. Human Papilloma Virus (HPV) self-sampling: do women accept it? J Obstet Gynaecol. 2018;38(3):402-7 doi:<https://dx.doi.org/10.1080/01443615.2017.1379061>.

77. Bansil P., Wittet S., Lim J.L., Winkler J.L., Paul P., Jeronimo J. Acceptability of self-collection sampling for HPV-DNA testing in low-resource settings: a mixed methods approach. BMC Public Health. 2014;14(1):596- doi:10.1186/1471-2458-14-596.

78. Behnke A.-L., Krings A., Wormenor C.M., Dunyo P., Kaufmann A.M., Amuah J.E. Female health-care providers' advocacy of self-sampling after participating in a workplace program for cervical cancer screening in Ghana: a mixed-methods study. Global Health Action. 2020;13(1):1-13 doi:10.1080/16549716.2020.1838240.

79. Modibbo F., Iregbu K.C., Okuma J., Leeman A., Kasius A., de Koning M., et al. Randomized trial evaluating self-sampling for HPV DNA based tests for cervical cancer screening in Nigeria. Infectious Agents & Cancer. 2017;12:1-9 doi:10.1186/s13027-017-0123-z.

80. Adedimeji A., Ajeh R., Dzudie A., Kendowo E., Fuhngwa N., Nsame D., et al. Cervical human papillomavirus DNA detection in women living with HIV and HIV-uninfected women living in Limbe, Cameroon. J Clin Virol. 2020;128:104445 doi:<https://dx.doi.org/10.1016/j.jcv.2020.104445>.

81. Awua A.K., Severini A., Wiredu E.K., Afari E.A., Zubach V.A., Adanu R.M.K. Self-Collected Specimens Revealed a Higher Vaccine- and Non-Vaccine-Type Human Papillomavirus Prevalences in a Cross-Sectional Study in Akuse. Advances in Preventive Medicine. 2020;2020:8343169 doi:10.1155/2020/8343169.

82. Bansil P., Lim J., Byamugisha J., Kumakech E., Nakisige C., Jeronimo J.A. Performance of Cervical Cancer Screening Techniques in HIV-Infected Women in Uganda. Journal of Lower Genital Tract Disease. 2015;19(3).

83. Belinson J.L., Qiao Y.L., Pretorius R.G., Zhang W.H., Rong S.D., Huang M.N., et al. Shanxi Province cervical cancer screening study II: self-sampling for high-risk human papillomavirus compared to direct sampling for human papillomavirus and liquid based cervical cytology. Int J Gynecol Cancer. 2003;13(6):819-26.

84. Bhatla N., Dar L., Patro A.R., Kumar P., Kriplani A., Gulati A., et al. Can human papillomavirus DNA testing of self-collected vaginal samples compare with physician-collected cervical samples and cytology for cervical cancer screening in developing countries? Cancer epidemiol. 2009;33(6):446-50 doi:<https://dx.doi.org/10.1016/j.canep.2009.10.013>.

85. Bogale A.L., Teklehaymanot T., Ali J.H., Kassie G.M., Medhin G., Baye A.Y., et al. Comparison of self-collected versus clinician collected cervicovaginal specimens for detection of high risk human papillomavirus among HIV infected women in Ethiopia. BMC Womens Health. 2022;22(1):360 doi:<https://dx.doi.org/10.1186/s12905-022-01944-2>.

86. Boggan J.C., Walmer D.K., Henderson G., Chakhtoura N., McCarthy S.H., Beauvais H.J., et al. Vaginal Self-Sampling for Human Papillomavirus Infection as a Primary Cervical Cancer Screening Tool in a Haitian Population. Sex Transm Dis. 2015;42(11):655-9 doi:<https://dx.doi.org/10.1097/OLQ.0000000000000345>.

87. Chen W., Jeronimo J., Zhao F.H., Qiao Y.L., Valdez M., Zhang X., et al. The concordance of HPV DNA detection by Hybrid Capture 2 and careHPV on clinician- and self-collected specimens. J Clin Virol. 2014;61(4):553-7 doi:<https://dx.doi.org/10.1016/j.jcv.2014.09.018>.

88. Chen K., Ouyang Y., Hillemanns P., Jentschke M. Excellent analytical and clinical performance of a dry self-sampling device for human papillomavirus detection in an urban Chinese referral population. J Obstet Gynaecol Res. 2016;42(12):1839-45 doi:<https://dx.doi.org/10.1111/jog.13132>.

89. Duan R., Zhao X., Zhang H., Xu X., Huang L., Wu A., et al. Performance of cervical cancer screening and triage strategies among women living with HIV in China. Cancer Med. 2021;10(17):6078-88 doi:10.1002/cam4.4152.

90. Elliott T., Kohler R.E., Monare B., Moshashane N., Ramontshonyana K., Muthoga C., et al. Performance of vaginal self-sampling for human papillomavirus testing among women living with HIV in Botswana. International Journal of STD and AIDS. 2019;30(12):1169-76 doi:<https://dx.doi.org/10.1177/0956462419868618>.

91. Feng N., Ezechi O., Uwandu M., Abimbola B.S., Vincent G.D., Idigbe I., et al. Self-collected versus medic-collected sampling for human papillomavirus testing among women in Lagos, Nigeria: a comparative study. BMC Public Health. 2022;22(1):1922 doi:<https://dx.doi.org/10.1186/s12889-022-14222-5>.

92. Garcia F., Barker B., Santos C., Brown E.M., Nuño T., Giuliano A., et al. Cross-sectional study of patient- and physician-collected cervical cytology and human papillomavirus. Obstetrics & Gynecology. 2003;102(2):266-72 doi:10.1016/s0029-7844(03)00517-9.

93. Holanda F., Jr., Castelo A., Veras T.M., de Almeida F.M., Lins M.Z., Dores G.B. Primary screening for cervical cancer through self sampling. Int J Gynaecol Obstet. 2006;95(2):179-84.

94. Jeronimo J., Bansil P., Lim J., Peck R., Paul P., Amador J.J., et al. A multicountry evaluation of care HPV testing, visual inspection with acetic acid, and Papanicolaou testing for the detection of cervical cancer. International Journal of Gynecological Cancer. 2014;24(3):576-85 doi:<https://dx.doi.org/10.1097/IGC.0000000000000084>.

95. Jones H.E., Allan B.R., van de Wijgert J.H., Altini L., Taylor S.M., de Kock A., et al. Agreement between self- and clinician-collected specimen results for detection and typing of high-risk human papillomavirus in specimens from women in Gugulethu, South Africa. J Clin Microbiol. 2007;45(6):1679-83.

96. Joseph N.T., Namuli A., Kakuhikire B., Baguma C., Juliet M., Ayebare P., et al. Implementing community-based human papillomavirus self-sampling with SMS text follow-up for cervical cancer screening in rural, southwestern Uganda. J. 2021;11:04036 doi:<https://dx.doi.org/10.7189/jogh.11.04036>.

97. Kamal E.M., El Sayed G.A., El Behery M.M., El Shennawy G.A. HPV detection in a self-collected vaginal swab combined with VIA for cervical cancer screening with correlation to histologically confirmed CIN. Arch Gynecol Obstet. 2014;290(6):1207-13 doi:<https://dx.doi.org/10.1007/s00404-014-3321-6>.

98. Longatto-Filho A., Roteli-Martins C., Hammes L., Etlinger D., Pereira S.M., Erzen M., et al. Self-sampling for human papillomavirus (HPV) testing as cervical cancer screening option. Experience from the LAMS study. Eur J Gynaecol Oncol. 2008;29(4):327-32.

99. Longatto-Filho A., Naud P., Derchain S.F., Roteli-Martins C., Tatti S., Hammes L.S., et al. Performance characteristics of Pap test, VIA, VILI, HR-HPV testing, cervicography, and colposcopy in diagnosis of significant cervical pathology. Virchows Arch. 2012;460(6):577-85 doi:<https://dx.doi.org/10.1007/s00428-012-1242-y>.

100. Lorenzato F.R., Singer A., Ho L., Santos L.C., Batista R.D., Lubambo T.M., et al. Human papillomavirus detection for cervical cancer prevention with polymerase chain reaction in self-collected samples. Am J Obstet Gynecol. 2002;186(5):962-8 doi:10.1067/mob.2002.122390.

101. Nilyanimit P., Wanlapakorn N., Niruthisard S., Takahashi M., Vongpunsawad S., Poovorawan Y. Comparison of Detection Sensitivity for Human Papillomavirus between Self-collected Vaginal Swabs and Physician-collected Cervical Swabs by Electrochemical DNA Chip. Asian Pac J Cancer Prev. 2014;15(24):10809-12 doi:10.7314/apjcp.2014.15.24.10809.

102. Qin Y., Zhang H.Y., Marlowe N., Fei M.D., Yu J., Lei X.Q., et al. Evaluation of human papillomavirus detection by Abbott m2000 system on samples collected by FTA Elute (TM) Card in a Chinese HIV-1 positive population. J Clin Virol. 2016;85:80-5 doi:10.1016/j.jcv.2016.11.002.

103. Quincy B.L., Turbow D.J., Dabinett L.N., Dillingham R., Monroe S. Diagnostic accuracy of self-collected human papillomavirus specimens as a primary screen for cervical cancer. J Obstet Gynaecol. 2012;32(8):795-9 doi:<https://dx.doi.org/10.3109/01443615.2012.717989>.

104. Safaeian M., Kiddugavu M., Gravitt P.E., Ssekasanvu J., Murokora D., Sklar M., et al. Comparability of self-collected vaginal swabs and physician-collected cervical swabs for detection of human papillomavirus infections in Rakai, Uganda. Sex Transm Dis. 2007;34(7):429-36.

105. Salmeron J., Lazcano-Ponce E., Lorincz A., Hernandez M., Hernandez P., Leyva A., et al. Comparison of HPV-based assays with Papanicolaou smears for cervical cancer screening in Morelos State, Mexico. Cancer Causes Control. 2003;14(6):505-12.

106. Senkomago V., Ting J., Kwatampora J., Gukare H., Mugo N., Kimani J., et al. High-risk HPV-RNA screening of physician- and self-collected specimens for detection of cervical lesions among female sex workers in Nairobi, Kenya. Int J Gynaecol Obstet. 2018;143(2):217-24 doi:<https://dx.doi.org/10.1002/ijgo.12628>.

107. Sowjanya A.P., Paul P., Vedantham H., Ramakrishna G., Vidyadhari D., Vijayaraghavan K., et al. Suitability of self-collected vaginal samples for cervical cancer screening in periurban villages in Andhra Pradesh, India. Cancer Epidemiol Biomarkers Prev. 2009;18(5):1373-8 doi:<https://dx.doi.org/10.1158/1055-9965.EPI-08-1171>.

108. Tiiti T.A., Mashishi T.L., Nkwinika V.V., Benoy I., Selabe S.G., Bogers J., et al. High-risk human papillomavirus detection in self-collected vaginal samples compared with healthcare worker collected cervical samples among women attending gynecology clinics at a tertiary hospital in Pretoria, South Africa. Virol J. 2021;18(1):192 doi:<https://dx.doi.org/10.1186/s12985-021-01662-5>.

109. Toliman P., Badman S.G., Gabuzzi J., Silim S., Forereme L., Kumbia A., et al. Field Evaluation of Xpert HPV Point-of-Care Test for Detection of Human Papillomavirus Infection by Use of Self-Collected Vaginal and Clinician-Collected Cervical Specimens. J Clin Microbiol. 2016;54(7):1734-7 doi:<https://dx.doi.org/10.1128/JCM.00529-16>.

110. Toliman P.J., Kaldor J.M., Badman S.G., Phillips S., Tan G., Brotherton J.M.L., et al. Evaluation of self-collected vaginal specimens for the detection of high-risk human papillomavirus infection and the prediction of high-grade cervical intraepithelial lesions in a high-burden, low-resource setting. Clin Microbiol Infect. 2019;25(4):496-503 doi:<https://dx.doi.org/10.1016/j.cmi.2018.05.025>.

111. Vega Crespo B., Neira V.A., Ortiz Segarra J., Rengel R.M., Lopez D., Orellana M.P., et al. Role of Self-Sampling for Cervical Cancer Screening: Diagnostic Test Properties of Three Tests for the Diagnosis of HPV in Rural Communities of Cuenca, Ecuador. Int J Environ Res Public Health. 2022;19(8):12 doi:<https://dx.doi.org/10.3390/ijerph19084619>.

112. Viviano M., Tran P.L., Kenfack B., Catarino R., Akaaboune M., Temogne L., et al. Self- versus physician-collected samples for the follow-up of human papillomavirus-positive women in sub-Saharan Africa. Int J Women Health. 2018;10:187-94 doi:<https://dx.doi.org/10.2147/IJWH.S154212>.

113. Wright T.C., Jr., Denny L., Kuhn L., Pollack A., Lorincz A. HPV DNA testing of self-collected vaginal samples compared with cytologic screening to detect cervical cancer. Jama. 2000;283(1):81-6.

114. Lack N., West B., Jeffries D., Ekpo G., Morison L., Soutter W.P., et al. Comparison of non-invasive sampling methods for detection of HPV in rural African women. Sex Transm Infect. 2005;81(3):239-41.

115. Campos N.G., Tsu V., Jeronimo J., Njama-Meya D., Mvundura M., Kim J.J. Cost-effectiveness of an HPV self-collection campaign in Uganda: comparing models for delivery of cervical cancer screening in a low-income setting. Health Policy Plan. 2017;32(7):956-68 doi:<https://dx.doi.org/10.1093/heapol/czw182>.

116. Campos N.G., Maza M., Alfaro K., Gage J.C., Castle P.E., Felix J.C., et al. The cost-effectiveness of implementing HPV testing for cervical cancer screening in El Salvador. Int J Gynaecol Obstet. 2019;145(1):40-6 doi:<https://dx.doi.org/10.1002/ijgo.12773>.

117. Flores Y.N., Bishai D.M., Lorincz A., Shah K.V., Lazcano-Ponce E., Hernandez M., et al. HPV testing for cervical cancer screening appears more cost-effective than Papanicolau cytology in Mexico. Cancer Causes Control. 2011;22(2):261-72 doi:<https://dx.doi.org/10.1007/s10552-010-9694-3>.

118. Olwanda E.E., Kahn J.G., Choi Y., Islam J.Y., Huchko M. Comparison of the costs of HPV testing through community health campaigns versus home-based testing in rural Western Kenya: a microcosting study. BMJ Open. 2020;10(10):e033979 doi:<https://dx.doi.org/10.1136/bmjopen-2019-033979>.

119. Shi J.F., Chen J.F., Canfell K., Feng X.X., Ma J.F., Zhang Y.Z., et al. Estimation of the costs of cervical cancer screening, diagnosis and treatment in rural Shanxi Province, China: a micro-costing study. BMC Health Serv Res. 2012;12:14 doi:10.1186/1472-6963-12-123.

120. Zhao X.L., Zhao S., Xia C.F., Hu S.Y., Duan X.Z., Liu Z.H., et al. Cost-effectiveness of the screen-and-treat strategies using HPV test linked to thermal ablation for cervical cancer prevention in China: a modeling study. BMC Med. 2023;21(1):149 doi:<https://dx.doi.org/10.1186/s12916-023-02840-8>.

121. Arrossi S., Thouyaret L., Herrero R., Campanera A., Magdaleno A., Cuberli M., et al. Effect of self-collection of HPV DNA offered by community health workers at home visits on uptake of screening for cervical cancer (the EMA study): a population-based cluster-randomised trial. Lancet Glob Health. 2015;3(2):e85-94 doi:<https://dx.doi.org/10.1016/S2214-109X(14)70354-7>.

122. Arrossi S., Paolino M., Thouyaret L., Laudi R., Campanera A. Evaluation of scaling-up of HPV self-collection offered by community health workers at home visits to increase screening among socially vulnerable under-screened women in Jujuy Province, Argentina. Implement Sci. 2017;12(1):17 doi:<https://dx.doi.org/10.1186/s13012-017-0548-1>.

123. Gizaw M., Teka B., Ruddies F., Abebe T., Kaufmann A.M., Worku A., et al. Uptake of Cervical Cancer Screening in Ethiopia by Self-Sampling HPV DNA Compared to Visual Inspection with Acetic Acid: A Cluster Randomized Trial. Cancer Prev Res (Phila Pa). 2019;12(9):609-16 doi:<https://dx.doi.org/10.1158/1940-6207.CAPR-19-0156>.

124. Moses E., Pedersen H.N., Mitchell S.M., Sekikubo M., Mwesigwa D., Singer J., et al. Uptake of community-based, self-collected HPV testing vs. visual inspection with acetic acid for cervical cancer screening in Kampala, Uganda: preliminary results of a randomised controlled trial. Trop Med Int Health. 2015;20(10):1355-67 doi:<https://dx.doi.org/10.1111/tmi.12549>.
