# Supplementary material for: Human papillomavirus self-sampling versus provider-sampling in low- and middle-income countries: a scoping review of accuracy, acceptability, cost, uptake, and equity
Source: Front Public Health. 2024 Nov 29;12:1439164. doi: 10.3389/fpubh.2024.1439164 (PMC11638174; doi:10.3389/fpubh.2024.1439164)
Supplement: Supplementary file 7 [file Table_7.docx]

# **Annex 7: Qualitative data on acceptability of HPV self-sampling in LMICs**

| Author, Year | Country & Population | Sub-theme | Finding | Sample quote |
| --- | --- | --- | --- | --- |
| Bakiewicz 2020 ([1](#_ENREF_1)) | Tanzania;  Women aged 25 to 60 years in a health care facility | Preference | Most of the women, (15, 70%) preferred self-sampling over a provider-based testing. | “I would prefer this one [pointing at the self- sampling device] [...] because it’s easy and comfortable.” (Participant) |
|  |  | Provider’s role in self-sampling | Majority of women did not believe in their own capabilities to conduct the sampling correctly, and therefore had a strong need for a health professional to be present in order to be comfortable doing it. | “I am uneducated [...] I feel more comfortable [ed. when a nurse is present] because I believe that the nurses are professionals. [...] I felt comfortable doing it with her.” (Participant) |
|  |  | Privacy | All women, except for one, expressed positive attitudes towards self-sampling and found it acceptable even though some also felt scared at first when seeing the device. | “[...] The first time I she saw this [ed. the self-sampling device], I was thinking: This is what?! Do I have to put it inside?! [...] I was afraid.” (Participant) |
| Megersa 2020 ([2](#_ENREF_2)) | Ethiopia;  Women mean age 36+9 who had participated in community wide home- based sampling | Perception | One participant mentioned not having benefited from her participation because she felt providing a self-collected sample breaks cultural obligations to be modest. | "I really felt bad about it. First, I thought it was easy, but the experience was not easy. [. . .] I was embarrassed to take the sample from my body (genitalia). To be honest, I did it for the sake of respecting the girls (sample collectors) who come to my home walking all that long distance. I was not totally happy about it.” (Participant) |
|  |  | Social influence | One participant mentioned having felt really embarrassed for providing the sample due to social stigmatization. | “[. . .] later, some of the residents of our local community insulted me for participating in the screening and everybody was blaming me because I said yes to those girls-sample collectors.” (Participant) |
|  |  | Barriers to acceptance of HPV sampling approaches | Some women did not see the need to provide the sample when they seemed healthy and lacked symptoms. | “I don’t see any benefits of this screening when I’m healthy and don’t have any symptoms of the disease; so, why I need be screened.” (Participant) |
|  |  | Religious influence | Some women felt self-sampling interfered with their religious beliefs and practices. | "There are misconceptions in the community; some women were not willing to participate particularly wives of religious leaders were not involved in the screening programme.” (Provider) |
| McFarlane 2021 ([3](#_ENREF_3)) | Jamaica;  Women aged 30 to 65 years | Preference | Most women preferred self-sampling and felt it provided more privacy in comparison to provider-sampling, which is more public. | “Many women feel self-conscious about a pap test, saying they can’t bother open up in front of the nurse […] It is too public.” (Participant) |
| Nyabigambo 2023 ([4](#_ENREF_4)) | Uganda;  WLHIV aged 25 to 49 years | Privacy | Some participants felt motivated to take self-sampling because of the privacy it offers. | “It is good because it is private, no opening up your legs for another person as they tell us  to open your legs wide when being screened by a midwife at the cervical cancer clinic.” (Participant) |
|  |  | Motivation to provider-sampling | Other participants felt motivated to have the test sample taken by a provider at the clinic because of early diagnosis and treatment. | “The health workers (HWs) are capable and all the instruments available. The health workers are skilled, even if they found you complicated, they know where to refer you very fast to test for whatever they want to know.” (Participants) |
|  |  | Perception | Some women had little knowledge and poor perception about cervical cancer screening. | “I don’t know anything, I just hear about the word cancer of the cervix, I don’t know its signs. (Participant)”  “Women don’t make screening a priority […] that way they will not come for testing, […].” (Provider) |
| Oketch 2019 ([5](#_ENREF_5)) | Kenya;  Women aged 25 to 65 years undergoing | Privacy | Others felt that the privacy allowed with self-sampling afforded them agency. | “If you go to the testing room, you are all alone just like in your own room, so you are just free.” (Participant) |
|  |  | Provider’s role in self-sampling | Few women felt the need for the provider’s presence. | “I would wish for the clinician to be there so that after getting tested and I end up being positive the doctor is able to [perform the] treatment already but not that I look for money and go to [Facility Name].” (Participant) |
|  |  | Preference | Most women felt good to collect the samples all by themselves. | “In relation to the new self-test kits that have emerged, it isn’t a must that the health care provider to be next to you.” (Participant) |
|  |  | Social influence | Some health care workers mentioned that women reported experiences of social stigmatization that comes with cervical cancer screening. | “Generally, people fear others seeing them going for screening because they might think one has the disease.” (Provider) |
| Brandt 2019 ([6](#_ENREF_6)) | Ethiopia;  Sexually active women between ages 20 and 65 in a health care facility | Barriers to acceptance of HPV sampling approaches | Some women felt no need of the screening at home if the treatment is not initiated after the test. | “If there is no treatment after the examination, people will not want to be screened for cervical cancer.” (Provider) |
| Allen-Leigh 2017 ([7](#_ENREF_7)) | Mexico;  Women 20 years and older in health care facility and community- based screening | Preference | Most women described HPV self-sampling as less painful and more comfortable | “We know each part of ourselves to do it, because […] we know how to do it to ourselves better, we know how to do it.” (Participant) |
| Rawat 2021 ([8](#_ENREF_8)) | Uganda  Women 18 years and older in a health facility | Social influence | Some participants felt gender roles and gender-based violence could influence the acceptability of self-sampling. | “I would need an approval from my partner first before providing the sample.” (Participant) |
| Saidu 2019 ([9](#_ENREF_9)) | Malawi; South Africa  Women from general population seeking primary screening and a referral population for colposcopy because of abnormal screening results | Preference | Some women preferred clinician sampling over self-sampling. | “I would rather have a doctor do it [sample]. When she opens down there, she can examine and see other things inside down there, which I would not be able to notice myself.” (Participant) |
|  |  | Barriers to acceptance of HPV sampling approaches | Most women suggested that it will be easier to integrate self-sampling at the clinics compared to sampling at homes. | “It’s waste of time and money coming here and going home and coming back here again whereas I could have done everything here all at once... and I will be worried of contaminating the sample or it [sample] drying up before bringing it back.” (Participant) |
|  |  | Privacy | Some women felt embarrassed to have the physician take the sample and therefore provided the samples themselves. | ‘I would choose to do it [sample] myself because I get embarrassed to expose myself to the doctor.” (Participant) |
| Arrossi 2016 ([10](#_ENREF_10)) | Argentina  Women present at home during cervical cancer and HPV screening visit | Social influence | Some women reported lack of confidentiality in trusted physician and the health care system. | “Everyone at the hospital is a gossip. When the results come out […] everybody reads them” (Participant) |
|  |  | Preference | Most women chose self-sampling over provider sampling because it helps them gain time to solve domestic responsibilities. | “It makes it easy to do it in your house, you don’t have to leave or go anywhere, and the woman can do it whenever she has time, at night when the kids go to sleep. She dedicates a little bit of time to it and does it herself, I think that’s why lots of women accepted the option of doing it in their homes” (Participant) |
| Van De Wijgert 2006 ([11](#_ENREF_11)) | South Africa  Sexually active women aged 18 years or older in health facility | Provider’s role in self-sampling | Most women in the focus group discussions reported not to like the speculum examinations but still chose to have the sample taken by the provider | “I do not trust my own capabilities to take adequate specimens…. I also want to have an opportunity to ask questions, although the speculum is very cold and painful” (Participant) |
|  |  | Privacy | Few women acknowledged the vitality of the clinician to use collect samples using a speculum but still chose self-sampling having feared exposing themselves to the clinician. | “Although it is the only way for a clinician to see the inside of a woman, it is not private….and it also takes long to wait at public clinics.” (Participant) |
| Bansil 2014 ([12](#_ENREF_12)) | India; Nicaragua; Uganda  Female staff members in health facility also for home-based testing | Provider’s role in self-sampling | Providers acknowledged their roles in instructing the patients and providing assurances regarding safety of the collection device. | “To me I think self-sampling is easily accepted as long as women are provided with sufficient information and they are given assurance about the safety of the brush.” (Provider) |
|  |  | Preference | Some women chose self-sampling over clinician’s sampling over privacy concerns. | “I did not have any difficulties inserting the brush. It is painless, better than having a medical person tell you to lie down on a bed, open your legs wide for visual inspection.” |
|  |  | Barriers to acceptance of HPV sampling approaches | Some women were worried of hurting themselves during the self-sampling process. | “To take the sample from that particular area is not visible to me. I may not do it correctly, and may hurt myself. I tried doing but could not do it.” (Participant) |
| Behnke 2020 ([13](#_ENREF_13)) | Ghana  Women aged 30 to 65 years in health facility | Preference | There was a strong preference among respondents for self-sampling over clinician sampling | “I think this one [self-sampling] is better – because the Pap smear, I have to come here and lie down for somebody to take the sample. Because there I think the privacy you are shy, you don’t want anybody to look at your private part or people you know around, your own colleagues, doing it for you. It’s better [if] you are in the comfort of your home and take your sample. So that one is better than the first one.” (Participant) |
|  |  | Provider’s role in self-sampling | A ward assistant indicated how self-experience was necessary to enable her to instruct patients on the self-sampling technique | “When it came, we did it, we have to do it and know how it is before we explain it to other people. If you don’t do it and then you are explaining it, they will not understand. You doing it will let you tell them that it’s easier.” (Provider) |

CC; Cervical Cancer: CIN; Cervical Intraepithelial Neoplasia: HPV; Human Papilloma Virus; WLHIV; Women living with HIV

# References

1. Bakiewicz A., Rasch V., Mwaiselage J., Linde D.S. "The best thing is that you are doing it for yourself" - perspectives on acceptability and feasibility of HPV self-sampling among cervical cancer screening clients in Tanzania: a qualitative pilot study. BMC Women's Health. 2020;20(1):1-9 doi:10.1186/s12905-020-00917-7.

2. Megersa B.S., Bussmann H., Barnighausen T., Muche A.A., Alemu K., Deckert A. Community cervical cancer screening: Barriers to successful home-based HPV self-sampling in Dabat district, North Gondar, Ethiopia. A qualitative study. PLoS ONE. 2020;15(12):e0243036 doi:<https://dx.doi.org/10.1371/journal.pone.0243036>.

3. McFarlane S.J., Morgan S.E. Evaluating Culturally-targeted Fear Appeal Messages for HPV Self-Sampling among Jamaican Women: A Qualitative Formative Research Study. Health Communication. 2021;36(7):877-90 doi:10.1080/10410236.2020.1723047.

4. Nyabigambo A., Mayega R.W., Hlongwana K., Ginindza T.G. Facilitators and Barriers to HPV Self-Sampling as a Cervical Cancer Screening Option among Women Living with HIV in Rural Uganda. Int J Environ Res Public Health. 2023;20(11):30 doi:<https://dx.doi.org/10.3390/ijerph20116004>.

5. Oketch S.Y., Kwena Z., Choi Y., Adewumi K., Moghadassi M., Bukusi E.A., et al. Perspectives of women participating in a cervical cancer screening campaign with community-based HPV self-sampling in rural western Kenya: a qualitative study. BMC Women's Health. 2019;19(1):N.PAG-N.PAG doi:10.1186/s12905-019-0778-2.

6. Brandt T., Wubneh S.B., Handebo S., Debalkie G., Ayanaw Y., Alemu K., et al. Genital self-sampling for HPV-based cervical cancer screening: a qualitative study of preferences and barriers in rural Ethiopia. BMC Public Health. 2019;19(1):N.PAG-N.PAG doi:10.1186/s12889-019-7354-4.

7. Allen-Leigh B., Uribe-Zuniga P., Leon-Maldonado L., Brown B.J., Lorincz A., Salmeron J., et al. Barriers to HPV self-sampling and cytology among low-income indigenous women in rural areas of a middle-income setting: a qualitative study. BMC Cancer. 2017;17(1):734 doi:<https://dx.doi.org/10.1186/s12885-017-3723-5>.

8. Rawat A., Sanders C., Mithani N., Amuge C., Pedersen H., Namugosa R., et al. Acceptability and preferences for self-collected screening for cervical cancer within health systems in rural Uganda: A mixed-methods approach. Int J Gynecol Obstet. 2021;152(1):103-11 doi:10.1002/ijgo.13454.

9. Saidu R., Moodley J., Tergas A., Momberg M., Boa R., Wright T., et al. South African women's perspectives on self-sampling for cervical cancer screening: A mixed-methods study. SAMJ S Afr Med J. 2019;109(1):47-52 doi:10.7196/SAMJ.2019.v109i1.13278.

10. Arrossi S., Ramos S., Straw C., Thouyaret L., Orellana L. HPV testing: a mixed-method approach to understand why women prefer self-collection in a middle-income country. BMC Public Health. 2016;16:832 doi:<https://dx.doi.org/10.1186/s12889-016-3474-2>.

11. Van De Wijgert J., Altini L., Jones H., De Kock A., Young T., Williamson A.L., et al. Two methods of self-sampling compared to clinician sampling to detect reproductive tract infections in Gugulethu, South Africa. Sexually Transmitted Diseases. 2006;33(8):516-23 doi:<https://dx.doi.org/10.1097/01.olq.0000204671.62529.1f>.

12. Bansil P., Wittet S., Lim J.L., Winkler J.L., Paul P., Jeronimo J. Acceptability of self-collection sampling for HPV-DNA testing in low-resource settings: a mixed methods approach. BMC Public Health. 2014;14(1):596- doi:10.1186/1471-2458-14-596.

13. Behnke A.-L., Krings A., Wormenor C.M., Dunyo P., Kaufmann A.M., Amuah J.E. Female health-care providers' advocacy of self-sampling after participating in a workplace program for cervical cancer screening in Ghana: a mixed-methods study. Global Health Action. 2020;13(1):1-13 doi:10.1080/16549716.2020.1838240.
