# Supplementary material for: Human papillomavirus self-sampling versus provider-sampling in low- and middle-income countries: a scoping review of accuracy, acceptability, cost, uptake, and equity
Source: Front Public Health. 2024 Nov 29;12:1439164. doi: 10.3389/fpubh.2024.1439164 (PMC11638174; doi:10.3389/fpubh.2024.1439164)
Supplement: Supplementary file 8 [file Table_8.docx]

# **Annex 8: Summary of the reported equity factors across studies based on the PROGRESS PLUS framework**

| Author, Year | Place of Residence | Race, Ethnicity, Culture/ Language | Occupation | Gender identity | Religion | Education | Socioeconomic status | Social capital | Age | Disability | Comorbidity |
| --- | --- | --- | --- | --- | --- | --- | --- | --- | --- | --- | --- |
| Dareng 2015 ([94](#_ENREF_94)) | Reported | Reported | Reported | Not reported | Reported | Reported | Reported | Not reported | Reported | Not reported | Not reported |
| Phoolcharoen 2018 ([65](#_ENREF_65)) | Reported | Reported | Not reported | Not reported | Reported | Reported | Reported | Not reported | Reported | Not reported | Not reported |
| Oberlin 2018 ([40](#_ENREF_40)) | Reported | Reported | Not reported | Not reported | Not reported | Reported | Reported | Not reported | Reported | Not reported | Reported |
| Moses 2015 ([155](#_ENREF_155)) | Reported | Not reported | Not reported | Not reported | Reported | Reported | Reported | Not reported | Reported | Not reported | Reported |
| Mitchell 2011 ([99](#_ENREF_99)) | Reported | Reported | Not reported | Not reported | Reported | Reported | Not reported | Not reported | Reported | Not reported | Reported |
| Awua 2017 ([93](#_ENREF_93)) | Not reported | Reported | Reported | Not reported | Reported | Reported | Not reported | Not reported | Reported | Reported | Not reported |
| Yoshida 2013 ([78](#_ENREF_78)) | Reported | Reported | Reported | Not reported | Not reported | Reported | Not reported | Not reported | Reported | Not reported | Not reported |
| Sormani 2022 ([68](#_ENREF_68)) | Not reported | Not reported | Reported | Not reported | Not reported | Reported | Reported | Not reported | Reported | Not reported | Reported |
| Rodrigues 2018 ([57](#_ENREF_57)) | Not reported | Reported | Reported | Not reported | Not reported | Reported | Not reported | Not reported | Reported | Not reported | Reported |
| Oketch 2019 ([107](#_ENREF_107)) | Reported | Not reported | Reported | Not reported | Not reported | Reported | Not reported | Not reported | Reported | Not reported | Reported |
| Modibbo 2017 ([61](#_ENREF_61)) | Reported | Not reported | Not reported | Not reported | Reported | Reported | Reported | Not reported | Reported | Not reported | Not reported |
| Mitchell 2017 ([47](#_ENREF_47)) | Reported | Not reported | Not reported | Not reported | Reported | Reported | Not reported | Not reported | Reported | Not reported | Reported |
| Manguro 2018 ([90](#_ENREF_90)) | Reported | Not reported | Reported | Not reported | Reported | Not reported | Not reported | Not reported | Reported | Not reported | Reported |
| Islam 2020 ([86](#_ENREF_86)) | Reported | Not reported | Reported | Not reported | Not reported | Reported | Not reported | Not reported | Reported | Not reported | Reported |
| Duan 2021 ([123](#_ENREF_123)) | Not reported | Reported | Reported | Not reported | Not reported | Reported | Not reported | Not reported | Reported | Not reported | Reported |
| Bakiewicz 2020 ([103](#_ENREF_103)) | Reported | Not reported | Not reported | Not reported | Reported | Reported | Not reported | Not reported | Reported | Not reported | Reported |
| Ahmad 2021 ([60](#_ENREF_60)) | Reported | Reported | Not reported | Not reported | Not reported | Reported | Reported | Not reported | Reported | Not reported | Not reported |
| Adamson 2015 ([96](#_ENREF_96)) | Reported | Not reported | Not reported | Not reported | Not reported | Reported | Reported | Not reported | Reported | Not reported | Reported |
| Abdullah 2018 ([38](#_ENREF_38)) | Reported | Reported | Not reported | Not reported | Not reported | Reported | Reported | Not reported | Reported | Not reported | Not reported |
| Wright 2000 ([145](#_ENREF_145)) | Reported | Not reported | Not reported | Not reported | Not reported | Reported | Not reported | Not reported | Reported | Not reported | Reported |
| Vega Crespo 2022b ([98](#_ENREF_98)) | Not reported | Not reported | Reported | Not reported | Not reported | Reported | Reported | Not reported | Reported | Not reported | Not reported |
| Vega Crespo 2022 ([144](#_ENREF_144))a | Not reported | Not reported | Reported | Not reported | Not reported | Reported | Reported | Not reported | Reported | Not reported | Not reported |
| Taku 2020 ([41](#_ENREF_41)) | Reported | Not reported | Not reported | Not reported | Not reported | Reported | Reported | Not reported | Not reported | Not reported | Reported |
| Sowjanya 2009 ([140](#_ENREF_140)) | Reported | Not reported | Not reported | Not reported | Reported | Reported | Not reported | Not reported | Reported | Not reported | Not reported |
| Saidu 2019 ([88](#_ENREF_88)) | Reported | Not reported | Not reported | Not reported | Not reported | Reported | Not reported | Not reported | Reported | Not reported | Reported |
| Rositch 2012 ([87](#_ENREF_87)) | Not reported | Not reported | Not reported | Not reported | Not reported | Reported | Reported | Not reported | Reported | Not reported | Reported |
| Quincy 2012 ([136](#_ENREF_136))b | Reported | Not reported | Not reported | Not reported | Not reported | Reported | Reported | Not reported | Reported | Not reported | Not reported |
| Qu 2023 ([89](#_ENREF_89)) | Not reported | Not reported | Reported | Not reported | Not reported | Reported | Reported | Not reported | Reported | Not reported | Not reported |
| Oraatanaphan 2014 ([64](#_ENREF_64)) | Reported | Not reported | Reported | Not reported | Not reported | Not reported | Reported | Not reported | Reported | Not reported | Not reported |
| Oneko 2022 ([70](#_ENREF_70)) | Reported | Not reported | Reported | Not reported | Not reported | Reported | Not reported | Not reported | Reported | Not reported | Not reported |
| Nyabigambo 2023 ([106](#_ENREF_106)) | Reported | Not reported | Not reported | Not reported | Reported | Reported | Not reported | Not reported | Not reported | Not reported | Reported |
| Mremi 2021 ([55](#_ENREF_55)) | Reported | Reported | Not reported | Not reported | Not reported | Reported | Not reported | Not reported | Not reported | Not reported | Reported |
| Mahande 2021 ([45](#_ENREF_45)) | Reported | Not reported | Reported | Not reported | Not reported | Reported | Not reported | Not reported | Reported | Not reported | Not reported |
| Katanga 2021 ([100](#_ENREF_100)) | Reported | Not reported | Not reported | Not reported | Not reported | Reported | Not reported | Not reported | Reported | Not reported | Reported |
| Hood 2020 ([74](#_ENREF_74)) | Not reported | Reported | Not reported | Not reported | Not reported | Reported | Reported | Not reported | Reported | Not reported | Not reported |
| Haile 2019 ([97](#_ENREF_97)) | Not reported | Not reported | Reported | Not reported | Not reported | Reported | Not reported | Not reported | Reported | Not reported | Reported |
| Esber 2017 ([72](#_ENREF_72)) | Not reported | Not reported | Not reported | Not reported | Not reported | Reported | Reported | Not reported | Reported | Not reported | Reported |
| Elliott 2019 ([124](#_ENREF_124)) | Not reported | Not reported | Reported | Not reported | Not reported | Reported | Not reported | Not reported | Reported | Not reported | Reported |
| Dzuba 2002 ([75](#_ENREF_75)) | Reported | Not reported | Not reported | Not reported | Not reported | Reported | Reported | Not reported | Reported | Not reported | Not reported |
| Brandt 2019 ([108](#_ENREF_108)) | Reported | Reported | Not reported | Not reported | Not reported | Reported | Not reported | Not reported | Reported | Not reported | Not reported |
| Viviano 2018 ([113](#_ENREF_113)) | Not reported | Not reported | Reported | Not reported | Not reported | Reported | Not reported | Not reported | Reported | Not reported | Not reported |
| Tiiti 2021 ([141](#_ENREF_141)) | Reported | Not reported | Reported | Not reported | Not reported | Not reported | Not reported | Not reported | Reported | Not reported | Not reported |
| Saidu 2021 ([138](#_ENREF_138)) | Not reported | Not reported | Not reported | Not reported | Not reported | Reported | Not reported | Not reported | Reported | Not reported | Reported |
| Safaeian 2007 ([137](#_ENREF_137)) | Reported | Not reported | Not reported | Not reported | Not reported | Not reported | Not reported | Not reported | Reported | Not reported | Reported |
| Rawat 2021 ([58](#_ENREF_58)) | Reported | Reported | Not reported | Not reported | Not reported | Reported | Not reported | Not reported | Not reported | Not reported | Not reported |
| Qin 2016 ([114](#_ENREF_114)) | Not reported | Not reported | Not reported | Not reported | Not reported | Reported | Not reported | Not reported | Reported | Not reported | Reported |
| Murchland 2019 ([39](#_ENREF_39)) | Not reported | Reported | Not reported | Not reported | Not reported | Reported | Not reported | Not reported | Reported | Not reported | Not reported |
| Megersa 2020 ([104](#_ENREF_104)) | Reported | Not reported | Not reported | Not reported | Not reported | Reported | Not reported | Not reported | Reported | Not reported | Not reported |
| Maza 2020 ([52](#_ENREF_52)) | Not reported | Not reported | Not reported | Reported | Not reported | Reported | Not reported | Not reported | Reported | Not reported | Not reported |
| Longatto-Filho 2008 ([133](#_ENREF_133)) | Not reported | Reported | Not reported | Not reported | Not reported | Reported | Not reported | Not reported | Reported | Not reported | Not reported |
| Khoo 2021 ([62](#_ENREF_62)) | Reported | Not reported | Reported | Not reported | Not reported | Not reported | Reported | Not reported | Not reported | Not reported | Not reported |
| Gottschlich 2019 ([35](#_ENREF_35)) | Not reported | Not reported | Not reported | Not reported | Reported | Reported | Not reported | Not reported | Reported | Not reported | Not reported |
| Gottschlich 2017 ([66](#_ENREF_66)) | Not reported | Reported | Not reported | Not reported | Not reported | Reported | Not reported | Not reported | Reported | Not reported | Not reported |
| Gizaw 2019 ([154](#_ENREF_154)) | Not reported | Not reported | Reported | Not reported | Not reported | Reported | Reported | Not reported | Not reported | Not reported | Not reported |
| Esber 2018 ([125](#_ENREF_125)) | Reported | Reported | Not reported | Not reported | Not reported | Not reported | Not reported | Not reported | Reported | Not reported | Not reported |
| Broquet 2015 ([95](#_ENREF_95)) | Reported | Not reported | Not reported | Not reported | Not reported | Reported | Not reported | Not reported | Reported | Not reported | Not reported |
| Bogale 2022 ([18](#_ENREF_18)) | Not reported | Not reported | Not reported | Not reported | Not reported | Reported | Not reported | Not reported | Reported | Not reported | Reported |
| Awua 2020 ([117](#_ENREF_117)) | Not reported | Not reported | Reported | Not reported | Reported | Reported | Not reported | Not reported | Not reported | Not reported | Not reported |
| Afzal 2020 ([73](#_ENREF_73)) | Reported | Not reported | Not reported | Not reported | Reported | Reported | Not reported | Not reported | Not reported | Not reported | Not reported |
| Wong 2018 ([76](#_ENREF_76)) | Not reported | Not reported | Not reported | Not reported | Not reported | Reported | Not reported | Not reported | Reported | Not reported | Not reported |
| Varun 2023 ([36](#_ENREF_36)) | Reported | Reported | Not reported | Not reported | Not reported | Not reported | Not reported | Not reported | Not reported | Not reported | Not reported |
| Van De Wijgert 2006 ([77](#_ENREF_77)) | Reported | Not reported | Not reported | Not reported | Not reported | Not reported | Not reported | Not reported | Reported | Not reported | Not reported |
| Tiiti 2021 ([56](#_ENREF_56)) | Reported | Not reported | Not reported | Not reported | Not reported | Not reported | Not reported | Not reported | Reported | Not reported | Not reported |
| Senkomago 2018 | Not reported | Not reported | Reported | Not reported | Not reported | Not reported | Not reported | Not reported | Reported | Not reported | Not reported |
| Salmeron 2003 ([112](#_ENREF_112)) | Reported | Not reported | Not reported | Not reported | Not reported | Not reported | Not reported | Not reported | Reported | Not reported | Not reported |
| Peedicayil 2014 ([69](#_ENREF_69)) | Not reported | Reported | Not reported | Not reported | Not reported | Not reported | Not reported | Not reported | Reported | Not reported | Not reported |
| Obiri-Yeboah 2017 ([82](#_ENREF_82)) | Not reported | Not reported | Not reported | Not reported | Not reported | Not reported | Not reported | Not reported | Reported | Not reported | Reported |
| Maza 2018 ([48](#_ENREF_48)) | Not reported | Not reported | Not reported | Not reported | Not reported | Reported | Not reported | Not reported | Reported | Not reported | Not reported |
| Mahomed 2014 ([42](#_ENREF_42)) | Reported | Not reported | Not reported | Not reported | Not reported | Not reported | Reported | Not reported | Not reported | Not reported | Not reported |
| Lorenzato 2002 ([135](#_ENREF_135)) | Not reported | Not reported | Not reported | Not reported | Not reported | Reported | Not reported | Not reported | Reported | Not reported | Not reported |
| Laskow 2017 ([71](#_ENREF_71)) | Not reported | Not reported | Not reported | Not reported | Not reported | Reported | Not reported | Not reported | Reported | Not reported | Not reported |
| Kamal 2014 ([131](#_ENREF_131)) | Not reported | Not reported | Reported | Not reported | Not reported | Reported | Not reported | Not reported | Not reported | Not reported | Not reported |
| Jones 2007 ([129](#_ENREF_129)) | Reported | Not reported | Not reported | Not reported | Not reported | Not reported | Reported | Not reported | Not reported | Not reported | Not reported |
| Jeronimo 2014 ([128](#_ENREF_128)) | Reported | Not reported | Not reported | Not reported | Not reported | Not reported | Not reported | Not reported | Reported | Not reported | Not reported |
| Flores 2021 ([19](#_ENREF_19)) | Reported | Not reported | Not reported | Not reported | Not reported | Not reported | Not reported | Not reported | Reported | Not reported | Not reported |
| Eche 2022 ([81](#_ENREF_81)) | Reported | Reported | Not reported | Not reported | Not reported | Not reported | Not reported | Not reported | Not reported | Not reported | Not reported |
| Eamratsameekool 2023 ([54](#_ENREF_54)) | Reported | Not reported | Reported | Not reported | Not reported | Not reported | Not reported | Not reported | Not reported | Not reported | Not reported |
| Bhatla 2009 ([120](#_ENREF_120)) | Reported | Not reported | Not reported | Not reported | Not reported | Not reported | Not reported | Not reported | Reported | Not reported | Not reported |
| Berner 2013 ([91](#_ENREF_91)) | Not reported | Not reported | Not reported | Not reported | Not reported | Reported | Not reported | Not reported | Reported | Not reported | Not reported |
| Behnke 2020 ([49](#_ENREF_49)) | Reported | Not reported | Reported | Not reported | Not reported | Not reported | Not reported | Not reported | Not reported | Not reported | Not reported |
| Bansil 2014 ([67](#_ENREF_67)) | Reported | Not reported | Not reported | Not reported | Not reported | Reported | Not reported | Not reported | Not reported | Not reported | Not reported |
| Arrossi 2016 ([59](#_ENREF_59)) | Reported | Not reported | Not reported | Not reported | Not reported | Reported | Not reported | Not reported | Not reported | Not reported | Not reported |
| Arrossi 2015 ([152](#_ENREF_152)) | Not reported | Not reported | Not reported | Not reported | Not reported | Reported | Not reported | Not reported | Not reported | Reported | Not reported |
| Allende 2019 ([102](#_ENREF_102)) | Not reported | Reported | Not reported | Not reported | Not reported | Reported | Not reported | Not reported | Not reported | Not reported | Not reported |
| Zhao 2023 ([150](#_ENREF_150)) | Reported | Not reported | Not reported | Not reported | Not reported | Not reported | Not reported | Not reported | Not reported | Not reported | Not reported |
| Rosenbaum 2014 ([85](#_ENREF_85)) | Not reported | Not reported | Not reported | Not reported | Not reported | Reported | Not reported | Not reported | Not reported | Not reported | Not reported |
| Possati-Resende 2020 ([53](#_ENREF_53)) | Not reported | Not reported | Not reported | Not reported | Not reported | Not reported | Not reported | Not reported | Reported | Not reported | Not reported |
| Olwanda 2020 ([148](#_ENREF_148)) | Reported | Not reported | Not reported | Not reported | Not reported | Not reported | Not reported | Not reported | Not reported | Not reported | Not reported |
| McFarlane 2021 ([105](#_ENREF_105)) | Reported | Not reported | Not reported | Not reported | Not reported | Not reported | Not reported | Not reported | Not reported | Not reported | Not reported |
| Mandigo 2015 ([51](#_ENREF_51)) | Not reported | Not reported | Not reported | Not reported | Not reported | Reported | Not reported | Not reported | Not reported | Not reported | Not reported |
| Madhivanan 2021 ([79](#_ENREF_79)) | Reported | Not reported | Not reported | Not reported | Not reported | Not reported | Not reported | Not reported | Not reported | Not reported | Not reported |
| Longatto-Filho 2012 ([134](#_ENREF_134)) | Not reported | Not reported | Not reported | Not reported | Not reported | Not reported | Not reported | Not reported | Reported | Not reported | Not reported |
| Lack 2005 ([132](#_ENREF_132)) | Reported | Not reported | Not reported | Not reported | Not reported | Not reported | Not reported | Not reported | Not reported | Not reported | Not reported |
| Kohler 2019 ([24](#_ENREF_24)) | Not reported | Not reported | Not reported | Not reported | Not reported | Reported | Not reported | Not reported | Not reported | Not reported | Not reported |
| Joseph 2021 ([130](#_ENREF_130)) | Not reported | Not reported | Not reported | Not reported | Not reported | Not reported | Not reported | Not reported | Not reported | Not reported | Reported |
| Huchko 2018 ([46](#_ENREF_46)) | Not reported | Not reported | Reported | Not reported | Not reported | Not reported | Not reported | Not reported | Not reported | Not reported | Not reported |
| He 2020 ([83](#_ENREF_83)) | Not reported | Not reported | Not reported | Not reported | Not reported | Reported | Not reported | Not reported | Not reported | Not reported | Not reported |
| Guan 2012 ([92](#_ENREF_92)) | Not reported | Not reported | Not reported | Not reported | Not reported | Reported | Not reported | Not reported | Not reported | Not reported | Not reported |
| Goldstein 2020 ([84](#_ENREF_84)) | Not reported | Not reported | Not reported | Not reported | Not reported | Not reported | Not reported | Not reported | Not reported | Not reported | Not reported |
| Chen 2016 ([110](#_ENREF_110)) | Not reported | Reported | Not reported | Not reported | Not reported | Not reported | Not reported | Not reported | Not reported | Not reported | Not reported |
| Chen 2014 ([122](#_ENREF_122)) | Reported | Not reported | Not reported | Not reported | Not reported | Not reported | Not reported | Not reported | Not reported | Not reported | Not reported |
| Boggan 2015 ([121](#_ENREF_121)) | Not reported | Not reported | Not reported | Not reported | Not reported | Not reported | Not reported | Not reported | Reported | Not reported | Not reported |
| Belinson 2003 ([119](#_ENREF_119)) | Not reported | Not reported | Not reported | Not reported | Not reported | Not reported | Not reported | Not reported | Reported | Not reported | Not reported |
| Bansil 2015 ([118](#_ENREF_118)) | Not reported | Not reported | Not reported | Not reported | Not reported | Not reported | Not reported | Not reported | Not reported | Not reported | Reported |
| Anand 2022 ([50](#_ENREF_50)) | Reported | Not reported | Not reported | Not reported | Not reported | Not reported | Not reported | Not reported | Not reported | Not reported | Not reported |
| Allen-Leigh 2017 ([109](#_ENREF_109)) | Reported | Not reported | Not reported | Not reported | Not reported | Not reported | Not reported | Not reported | Not reported | Not reported | Not reported |
| Number of studies repoting per each equity factor | 59 | 23 | 27 | 1 | 14 | 68 | 23 | 0 | 69 | 2 | 29 |
